# A Multicenter, Randomized, Double-Blinded, Clinical Trial Comparing Cattell-Warren and Blumgart Anastomoses Following Partial Pancreatoduodenectomy: PANasta Trial

**DOI:** 10.1097/AS9.0000000000000198

**Published:** 2022-09-15

**Authors:** Christopher M. Halloran, John P. Neoptolemos, Richard Jackson, Kellie Platt, Eftychia-Eirini Psarelli, Srikanth Reddy, Dhanwant Gomez, Derek A. O’Reilly, Andrew Smith, Thomas M. Pausch, Andreas Prachalias, Brian Davidson, Paula Ghaneh

**Affiliations:** From the * University of Liverpool, Liverpool, UK; † Liverpool University Hospitals Foundation Trust, Liverpool, UK; ‡ University of Heidelberg, Heidelberg, Germany; § Oxford University Hospitals, Oxford, UK; ∥ Queens Medical Centre, Nottingham, UK; ¶ Manchester Royal Infirmary, Manchester, UK; # Beijing United Family Hospital and Clinics, Beijing, China; ** St James’s University Hospital, Leeds, UK; †† Kings College Hospital, London, UK; ‡‡ University College London, UK; §§ Royal Free Hospital, London, UK.

**Keywords:** pancreatico-jejunostomy, blumgart anastomosis, cattell-warren anastomosis, post operative pancreatic fistula, complications, survival, pancreatic adenocarcinoma

## Abstract

**Objective:**

Whether a Blumgart anastomosis (BA) is superior to Cattell-Warren anastomosis (CWA) in terms of postoperative pancreatic fistula (POPF) following pancreatoduodenectomy.

**Importance:**

Complications driven by POPF following pancreatic cancer resection may hinder adjuvant therapy, shortening survival. BA may reduce complications compared to CWA, improving the use of adjuvant therapy and prolonging survival.

**Methods:**

A multicenter double-blind, controlled trial of patients undergoing resection for suspected pancreatic head cancer, randomized during surgery to a BA or CWA, stratified by pancreatic consistency and duct diameter. The primary end point was POPF, and secondary outcome measures were adjuvant therapy use, specified surgical complications, quality of life, and survival from the date of randomization. For a 10% POPF reduction, 416 patients were required, 208 per arm (two-sided α = 0·05; power = 80%).

**Results:**

Z-score at planned interim analysis was 0.474 so recruitment was held to 238 patients; 236 patients were analyzed (112 BA and 124 CWA). No significant differences in POPF were observed between BA and CWA, odds ratio (95% confidence interval [CI]) 1·04 (0.58–1.88), *P* = 0.887, nor in serious adverse events. Adjuvant therapy was delivered to 98 (62%) of 159 eligible patients with any malignancy; statistically unrelated to arm or postoperative complications. Twelve-month overall survival, hazard ratio (95% CI), did not differ between anastomoses; BA 0.787 (0.713–0.868) and CWA 0.854 (0.792–0.921), *P* = 0.266, nor for the 58 patients with complications, median (IQR), 0.83 (0.74–0.91) compared to 101 patients without complications 0.82 (0.76–0.89) (*P* = 0.977).

**Conclusions:**

PANasta represents the most robust analysis of BA versus CWA to date.

## Abstract 2

Mini Abstract: Postoperative pancreatic fistulae were not dissimilar between Blumgart and Cattell-Warren anastomoses. Although POPF drive postoperative complications they do not affect the rate of adjuvant treatment administration nor affect overall patient survival. As efficacy is similar between BA and CWA, the choice of anastomosis can be left to surgical discretion.

Supplemental Digital Content is available in the text.

## INTRODUCTION

Pancreatic ductal adenocarcinoma (PDAC) is a major surgical and oncological challenge^1–3^ with only 20% of patients presenting with localized disease and without metastases undergoing surgical resection.^1^ The 5-year survival rates increase from an estimated 8% with surgery alone to 30%–50% with adjuvant combination cytotoxic regimens.^4–6^ There is some evidence to support the use of preoperative neo-adjuvant therapy in locally advanced disease^1,7^ aiming to improve resectabilty and/or overall survival rates.^8–11^ However, the proportion of patients who overcome surgical complications well enough in the first instance to commence adjuvant therapy is uncertain.^5,6,12,13^

The driver of serious postoperative complications following pancreatic head resection is postoperative pancreatic fistula (POPF) arising from failure of the pancreatic remnant anastomosis.^14,15^ Most POPF are harmless biochemical rises (type A). However, CR-POPF (type B or C) changes clinical care and may initiate systemic complications and can lead to death. ^13,16,17^

The International Study group of Pancreatic Surgery (ISGPS) recommends the use of a pancreatojejunostomy,^18^ following pancreatoduodenectomy. This is typified by the Cattell-Warren anastomosis (CWA) with an inner pancreatic duct-to-jejunum mucosal anastomosis, and a second outer layer between the anterior and posterior cut edges of the pancreatic remnant to the seromuscular layer of the jejunum.^19^ This type of reconstruction is associated with a high rate of CR-POPF, in the region of 23%, when undertaken in patients with a soft pancreatic texture and a main pancreatic duct maximum diameter ≤3 mm.^20^ An alternative pancreatojejunal reconstruction is the Blumgart anastomosis (BA) which also involves a similar inner duct-to-mucosa anastomosis, but the outer layer is a full-thickness jejunal wrap-around of the pancreatic stump.^21^ Nonrandomized comparative studies suggest the BA substantially reduces the rate of CR-POPF compared to the CWA.^22–24^ The PANasta trial was designed to be the first multicenter, masked randomized trial to compare these 2 anastomotic techniques with the rate of POPF (any grade) as the primary outcome measures and key secondary outcome measures included other specified postpancreatectomy complications, the proportion of patients commencing adjuvant therapy, and overall survival from the date of randomization before resection.

## METHODS

This blinded multicenter two-arm randomized controlled trial was conducted at 7 UK specialist pancreas centers, coordinated from the Cancer Research UK (CRUK) Liverpool Cancer Trials Unit.

### Patient Selection

Patients with suspected peri-pancreatic head malignancy underwent standard evaluation^25^ before local multidisciplinary team (MDT) discussion. Histological diagnosis of malignancy before surgery, was not necessary, provided that the MDT outcome was to proceed with pancreas head resection.^26–29^ Patients were eligible if they were due to undergo an elective pancreatoduodenectomy for presumed malignancy, understood the nature or consequences of the trial, were able to provide written informed consent, and be aged 18 years or older. Patients were excluded if they were due to undergo extended partial pancreatoduodenectomy; left, central, or total pancreatectomy; arterial resection or multivisceral resection, previous pancreatic resection, surgery for known chronic pancreatitis, recruitment to any other pancreatic resection trial; women of childbearing potential or were unable or unwilling to use adequate contraception from the time of consent up to the day of surgery (this latter point was stipulated under the terms of the sponsor, the University of Liverpool, and regulatory requirements of the Liverpool Clinical Trials Unit). Patients who had undergone neo-adjuvant chemotherapy with or without radiotherapy, were excluded as manifestations of POPF in such a case, would be more likely related to that of systemic treatments rather than the anastomosis construction itself.

### Randomization and Blinding

Eligible patients were randomized using a 1:1 allocation ratio using randomly permuted blocks including pancreatic texture (soft vs hard), pancreatic duct diameter (≤3 vs >3 mm), and research site as stratification factors. Randomization was undertaken intraoperatively by the operating surgeon, following pancreatic head excision and before reconstruction, via a bespoke password-controlled web-based tool called the Treatment Allocation Randomization System (TARDIS), allocations were time-stamped. Patients and site staff were blinded to the treatment allocation, with the surgeon stating in the operation notes “pancreatic anastomosis was constructed according to trial protocol.”

### Procedures

The index procedure was pancreatoduodenectomy undertaken either as pylorus-preserving partial pancreatoduodenectomy (PPPD) or a Kausch-Whipple partial pancreatoduodenectomy with distal partial gastrectomy (KW-PD), dependent upon the clinical requirements. A single jejunal limb was brought up to the pancreas for the pancreatic anastomosis, either a CWA or a BA, as detailed in Halloran et al.^25^ The same jejunal loop was then used to anastomose the bile duct and either the first part of the duodenum or gastric remnant stomach. The placement of an internal pancreatic duct stent across the duct-mucosal anastomosis was mandatory for all patients. Surgical drains were positioned in proximity to the pancreatic, biliary, and gastric anastomoses. 100 μg of octreotide was administered subcutaneously on the evening before surgery and 100 μg three times a day subcutaneously on the day of surgery (day 0) and on postoperative days 1 to 6 to all patients.

### Standardization and Quality Assessment

The standardization of the operative techniques was ensured by using modified methods developed with the MRC ConDuCT-II Trials Methodology Hub (Supplemental Material A and B, http://links.lww.com/AOSO/A163).^25,30,31^ Notably:

1. Consensus meetings: All center leads agreed the essentials of each anastomosis and the likely key steps, the postoperative management of drains, pancreatic duct stents, the use of octreotide, and the timing of operative photographs. This information was developed into an operative manual.
2. Operative manual: A finalized operative manual for each anastomosis contained steps that were (a) mandatory to the construction of a safe anastomosis; (b) prohibited for the construction of a safe anastomosis, and (c) flexible steps where the operating surgeon can choose a method.
3. Operative photographs: Digital operative photographs detailing procedures in a step-by-step method and showing the mandated photographic documentation of the 3 elements of reconstruction: adequate pancreatic neck mobilization, insertion of the parenchymal sutures, and in detail the sutures to the main pancreatic duct prior to tying, and finally of the completed anastomosis. Photographs were centrally reviewed to assess quality of the procedure and ensure consistency. Immediately following the end of surgery, the pictures were uploaded to a secure portal area of the trial (Supplemental Material A and C, http://links.lww.com/AOSO/A163). Photographs for each case were examined by two reviewers (C.M.H. and D.G.) to determine the nature and quality of the procedure. In cases where there was no agreement, a third reviewer (D.A.O.) was involved to reach a consensus. All reviewers were blinded to the patient allocation.

### Follow-Up

Each patient had 6 trial visits: An enrollment visit, a visit on the day of surgery, and follow-up visits to assess outcomes on the day of discharge, and at 3, 6, and 12 months after surgery. The detailed schedule is provided in the protocol (Supplemental Material B, http://links.lww.com/AOSO/A163).

### Outcomes

The primary outcome POPF (any grade).^14^ Secondary outcome measures were administration of adjuvant therapy or entry into clinical trials of adjuvant therapy; operation time; delayed gastric emptying; rates of wound infection, pulmonary infection, postoperative fluid collection, intra and postoperative bleeding, reoperation and venous thromboembolism; hospital stay; generic quality of life (EQ5D) and the European Organization for Research and Treatment of Cancer (EORTC) cancer specific questionnaire (QLQ-C30); health economic assessments; and survival from the date of randomization until death by any cause. Patients underwent adjuvant chemotherapy if eligible: Strong indication had PDAC^4,6^; relative indication ampullary adenocarcinoma,^27^ intrapancreatic bile duct adenocarcinoma,^28^ or periampullary duodenal adenocarcinoma.^29^ Specific complications and severity were defined by those of the ISGPS and the Dindo and Clavien classification for all other major complications.^14,16–18,32,33^ All adverse and serious adverse events (SAEs) were recorded from the time of surgery up until commencement of adjuvant chemotherapy. Expected events for the trial were exempt from SAE reporting unless they were classified as life- threatening or resulted in death namely grades 4 and 5. Trial follow-up ceased 12 months following randomization. Updated protocol to version 6.0 on March 22, 2016.

### Statistical Analysis

#### Sample Size

POPF was measured as a binary outcome assuming a rate of 20% in the CWA standard treatment arm and an assumed decrease down to 10% or less in the BA test arm.^15,19,21^ Using a two-sided α level of 0.05 and a power of 80%, 416 patients were required with 208 per arm. The sample size was estimated inclusive of a single interim analysis when 50% of the final information was available. Sample sizes were inflated to account for both noncompliance estimated at 15%, and a 3% loss-to-follow-up, equating to a final sample size of 506 patients with 253 per arm. Stopping rules for the interim analysis were based on the standardized Z-score based on an O’Brien Fleming^34^ 2 stage design using the SAS PROC SEQDESIGN version 9.3. The study would stop for futility if the Z-score was in the interval −0.698 to 0.698 and would stop for efficacy if the Z score was outside the interval −2.736 to 2.736.

#### Analysis Method

Continuous data are summarized as median (IQR) and categorical data are summarized as frequencies of counts with associated percentages with tests across treatment or other patients sub-groups performed using the Wilcox test for continuous covariates and fisher test/Chi-square test for categorical data. Analysis is performed on an intention to treat (ITT) retaining all patients in their randomized groups irrespective of any protocol deviations. No adjustment for missing data was planned and analyses were performed on a complete case basis. A *P* value of 0.05 was used to determine statistical significance with estimated effects presented alongside 95% confidence intervals (CIs). Efficacy of the primary outcome was measured using an odds ratio and the comparison of fistula between treatment groups was performed using a stratified Cochran-Mantel-Haenszel test. Comparisons of all binary secondary endpoints followed the same methodology. The time to start of adjuvant therapy was analyzed as a time-to-event endpoint, estimates obtained through the Kaplan–Meier^35^ approach and comparisons between groups performed using a log-rank test.^36^ Continuous secondary outcomes were measured between groups using mean differences and compared using a t-test. The accuracy of a Fistula Rate Score (FRS)^37^ was tested using calculation of area under the receiver operating curve (AUC).^38^

## RESULTS

The first patent was enrolled on May 15, 2015. Following the planned interim analysis of the first 208 evaluable patients, a Z-score comparing the 2 treatment groups of 0.474 was observed. Both the Independent Data and Safety Monitoring Committee and the Trial Steering Committee recommended closure and the last patient was recruited on August 7, 2017. Following the minimum follow-up for all recruited patients, a data lock was implemented on March 1, 2019 and the final analyses were performed.

### Patient Demographics

**FIGURE 1.**
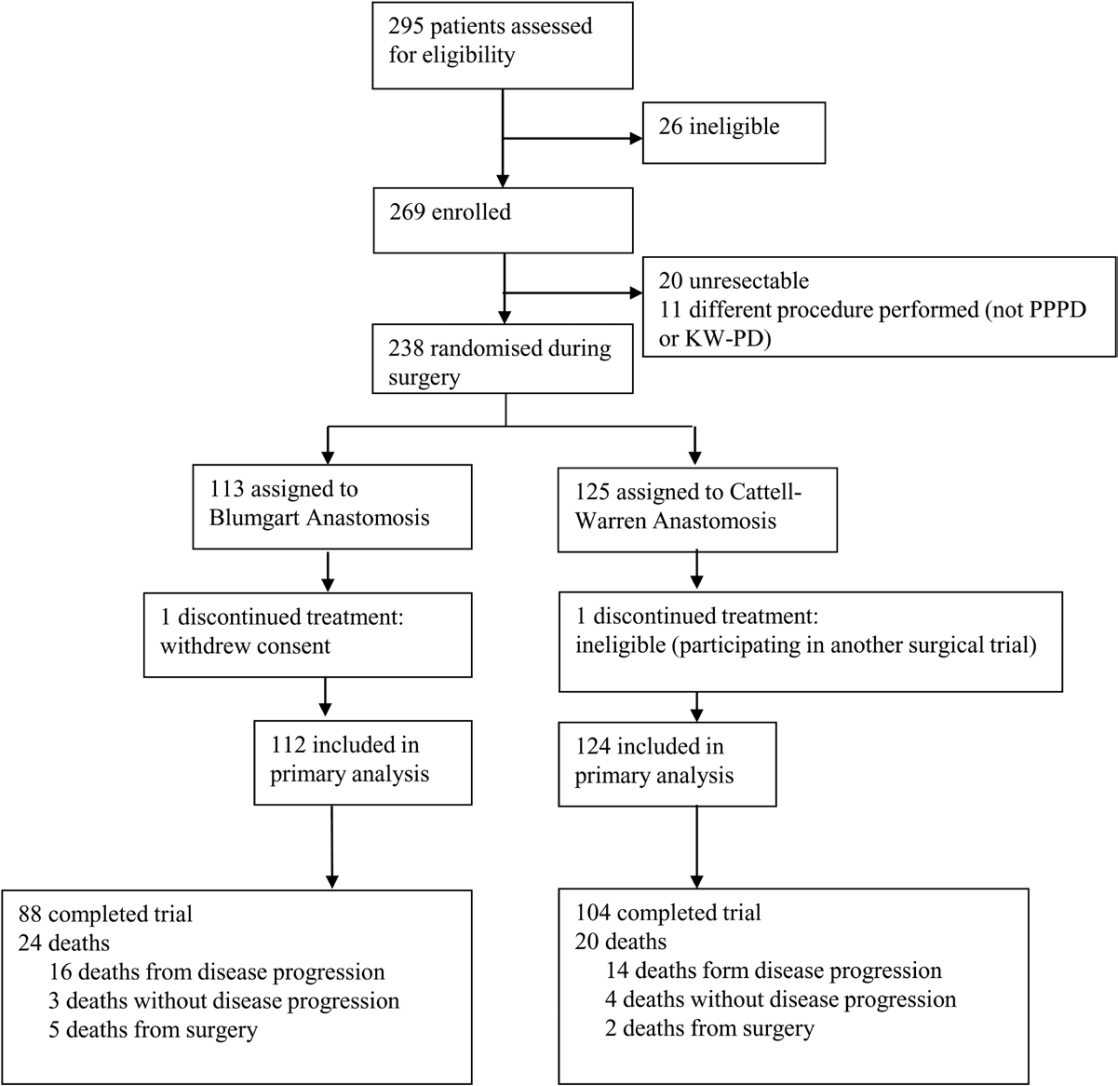
Consort diagram.

Two hundred thirty-eight patients who were successfully randomized at operation (Fig. 1). Two patients who had been randomized were removed from the study analysis in line with the ITT definition specified in the protocol, leaving 124 were allocated to undergo a CWA and 112 were allocated to undergo a BA. The baseline of these and other patient demographics by randomized allocation are shown in Supplemental Table 1, http://links.lww.com/AOSO/A162. Histology revealed a malignant lesion in 194 (82.5%) of 235 patients (missing value for 1 patient allocated to the CWA group), and a nonmalignant lesion in the remaining 41 (17.5%) patients.

### Postoperative Pancreatic Fistulae and Complications

Postoperatively, 60 (25%) patients developed a pancreatic fistula (any POPF), in 28 (25%) of 112 patients following a BA, and in 32 (26%) of 124 patients following a CWA. Thirty-three (14%) were type A POPFs, 22 (9%) were type B, and 5 (2%) were type C with no significant differences between allocated groups (Supplemental Table 2, http://links.lww.com/AOSO/A162). Any POPF occurred in 13 (10%) of 134 patients with a hard pancreas compared to 47 (46%) of 102 patients with a soft or normal pancreas (odds ratio = 0.13, 95% CI = 0.06–0.26; *P* < 0.001). Clinically relevant fistulae, types B and C (CR-POPF) occurred in 6 (4%) of 134 patients with a hard pancreas compared to 16 (16%) of 102 patients with a soft or normal pancreas (odds ratio = 0·25, 95% CI = 0.08–0.72; *P* = 0.253). Any POPF occurred in 47 (32%) of 147 patients with a main pancreatic diameter ≤3 mm compared to 13 (15%) of 89 patients with a main pancreatic diameter >3 mm (odds ratio = 2.74, 95% CI = 1.34–5.92; *P* = 0.03). CR-POPF occurred in 19 (13%) out of 147 patients with a main pancreatic diameter ≤3 mm compared to 2 (2%) of 89 patients a main pancreatic diameter >3 mm (odds ratio = 6.42, 95% CI = 1.49–58.17; *P* = 0.004), Supplemental Table 2, http://links.lww.com/AOSO/A162. There were no significant differences between the allocated groups with regards to operation time, length of hospital stay nor with regards to any of the other specified postoperative complications.

Postoperative complications associated with the presence of any POPF and CR-POPF are shown in Table 1. Patients with any POPF when compared to patients with no POPF were more likely to have delayed gastric emptying (29 [48%] of 60 vs 45 [26%] of 174 patients; *P* = 0·002), larger fistula volumes (median volume [IQR] ml = 2526 [1418, 13,128] vs 111 [40, 343]; *P* < 0.001) and a prolonged hospital stay (median [IQR] days = 16 [11, 28] vs 12 [9, 17]; *P* < 0.001). Both fistula volume and prolonged hospital stay were also associated with higher grades of fistula (Table 1). The FRS based on pancreatic texture, main pancreatic duct diameter, and body mass index when applied to the whole data set had an AUC (95% CI), of 0.61 (0.53–0.68) for any POPF and 0.54 (0.44–1.64) for CR-POPF (Supplemental Material A, http://links.lww.com/AOSO/A163).

**TABLE 1.** Association Between Postoperative Pancreatic Fistula and Other Postoperative Complications

Secondary Outcome		No POPF (n = 176)	Any POPF (n = 60)	*P* No POPF vs Any POPF	Type A POPF (n = 33)	CR-POPF (n = 27)	*P* Type A POPF vs Type B/CPOPF
Intraoperative hemorrhage median (IQR), mL		600 (400, 900)	800 (425, 1000)	0·365	500 (400, 931.25)	825 (500, 1000)	0.119
Missing Data	N = 2						
Postoperative hemorrhage, N (%)	No *N* = 191	146 (84)	45 (75)		25 (76)	20 (74)	
	Yes N = 43	28 (16)	15 (25)	0.179	8 (24)	7 (26)	1
Missing data	*N* = 2	n=2	··	··	··	··	
Delayed gastric emptying, N (%)	No N = 160	129 (74)	31 (52)		20 (6)	11 (41)	
	Yes N = 74	45 (26)	29 (48)	**0.002**	13 (39%)	16 (59)	0.203
Missing data	N = 2	n = 2	··		··	··	
Wound infection, N (%)	No N = 191	146 (84)	45 (75)		25 (76)	20 (74)	
	Yes N =43	28 (16)	15 (25)	0.179	8 (24)	7 (26)	1
Missing data	N = 2	n = 2	··		··	··	
Pulmonary infection, N (%)	No N = 212	162 (93)	50 (83)		28 (85)	22 (81)	
	Yes N = 22	12 (7)	10 (17)	0.048	5 (15)	5 (19)	1
Missing data	N = 2	n = 2	··		··	··	
Postoperative surgical drain fluid collection (fistula volume), median (IQR), mL		111 (40, 342·5)	2526 (1418, 13,128)	**<0.001**	1860 (1279, 3691)	8774 (1612, 35,139)	**0.014**
Missing data	N = 2						
Reoperation rate, N (%)	No N = 200	148 (94)	52 (88)		32 (97)	20 (77)	
	Yes N = 17	10 (6)	7 (12)	0·286	1 (3)	6 (23)	0.037
Missing data	N = 19	n=18	n=1				
Venous thromboembolism, N (%)	No N = 224	167 (96)	57 (95)		33 (100)	24 (89)	
	Yes N = 10	7 (4)	3 (5)	1	0 (0)	3 (11)	0.085
Missing data	N = 2						
Postoperative hospital stay, median (IQR), days		12 (9, 17)	16 (11, 28)	**<0.001**	12 (9, 16)	27.5 (21, 41)	**<0.001**
Missing data	N = 2						
Bold indicates significant results.							

### Quality and Safety

No concerns regarding the technical quality of the anastomosis construction were revealed. However, 6 (2.5%) anastomoses were identified that differed from the randomized allocation but were retained on an ITT basis: 4 patients randomized to CWA, underwent BA, and in 2 patients randomized to BA, underwent a CWA instead.

There were 39 SAEs reported in 31 (13%) of the 236 patients (Supplemental Table 3, http://links.lww.com/AOSO/A162). There were 21 SAEs observed from 16 (13%) of 124 patients undergoing a CWA and 18 events from 15 (13%) of 112 patients undergoing a BA. There were 10 grade V SAEs, leading to surgically related deaths in 7 patients (3%), 1 further patient developed liver metastases on restaging before adjuvant chemotherapy.

### Adjuvant Treatment and Survival

**FIGURE 2.**
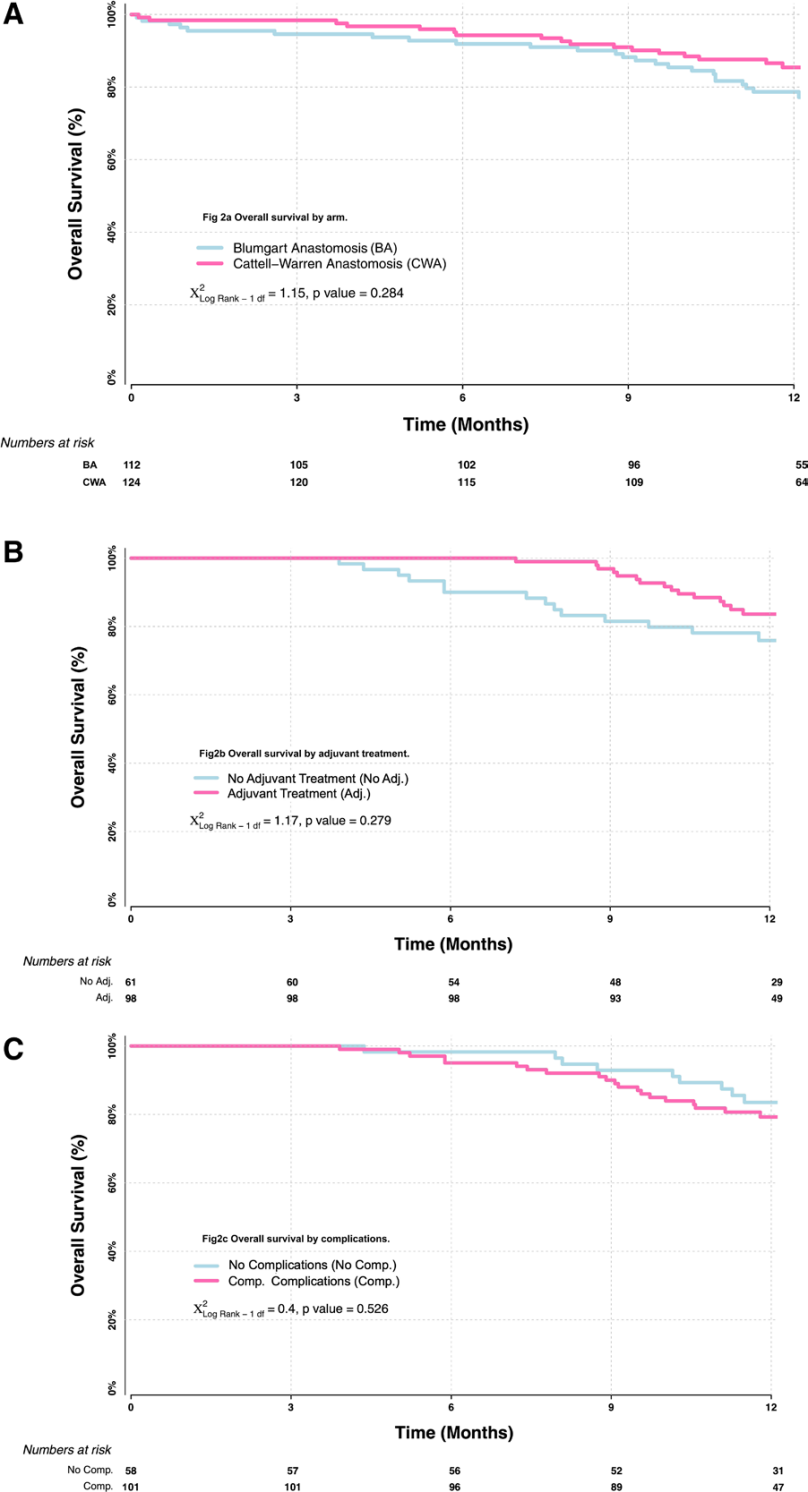
Survival. A, Overall survival by arm. B, Overall survival by adjuvant treatment. C, Overall survival by complications.

Overall survival by randomization arm showed no statistically significant differences between the BA and CWA groups, with a hazard ratio of 0.72 (95% CI = 0.40–1.31; *P* = 0.266) (Supplemental Table 2, http://links.lww.com/AOSO/A162 and Fig. 2A). There were 174 patients randomized who could potentially be considered for adjuvant therapy, including those with a definite indication, PDAC (75), and those with relative indications cholangiocarcinoma (50), ampullary adenocarcinoma (40), and duodenal adenocarcinoma (9) (Table 2). There were 15 patients with missing data leaving 159 patients; adjuvant therapy was delivered to 98 (62%), and in those with PDAC, this was 51 (78%) of 65 patients (Table 2). Overall survival by adjuvant therapy for these 159 patients demonstrated a hazard ratio of 0.51 (95% CI = 0.24–1.10; *P* = 0.176) in favor of those that had adjuvant therapy (Fig. 2B). There was no difference in overall survival by the development or absence of postoperative complications (Table 3 and Fig. 2C).

**TABLE 2.** Adjuvant Therapy in Relation to Complications and Survival

Patient Groups	Levels	BA	CWA	*P*	Missing Data: Adjuvant Therapy Yes	Missing Data: Adjuvant Therapy No	No Adjuvant Chemotherapy	Adjuvant Chemotherapy	*P*	Three Month Adjuvant Chemotherapy Rate	12-month Overall Survival Rate			Hazard Ratio (95% CI)	*P*
											No Adjuvant Chemotherapy	Adjuvant Chemotherapy	Total		
All Patients N = 236^*^	No complication	41	43	··	11	0	23	42	··	0.58 (0.48–0.7)	0.8 (0.6–0.94)	0.85 (0.75–0.97)	0.83 (0.75–0.91)	0.74 (0.319–1.695)	0.471
	Complication	70	80	··	6	0	49	62	··	0.7 (0.63–0.78)	0.8 (0.72–0.89)	0.84 (0.76–0.94)	0.82 (0.76–0.89)	0.84 (0.416–1.705)	0.633
	Total	111	123	0.863	17	1	72/176 (41%)	104/176 (59%)	0.326	0.65 (0.6–0.72)	0.8 (0.73–0.87)	0.85 (0.78–0.92)	0.82 (0.77–0.87)	0.8 (0.437–1.455)	0.46
PDAC N = 75	No complication	18	17	··	8	0	5	22	··	0.38 (0.25–0.59)	0.76 (0.56–1)	0.9 (0.79–1)	0.85 (0.74–0.98)	**0.18** **(0.039**–**0.84**)	**0.029**
	Complication	20	20	··	2	0	9	29	··	0.37 (0.24–0.56)	0.44 (0.22–0.87)	0.78 (0.64–0.95)	0.68 (0.54–0.85)	0.49 (0.175–1.386)	0.18
	Total	38	37	0.399	10	0	14/65 (22%)	51/65 (78%)	0.850	0.38 (0.28–0.5)	0.6 (0.43–0.85)	0.83 (0.73–0.95)	0.76 (0.67–0.87)	**0.35(0.133**–**0.895**)	0.029
Cholangiocarcinoma N = 50	No Complication	11	4	··	2	0	9	4	··	0.69 (0.48–0.99)	0.62 (0.39–1)	1 (1–1)	0.73 (0.53–1)	0 (0, Inf)	0.998
	Complication	18	17	··	2	0	17	16	··	0.7 (0.56–0.87)	0.63 (0.45–0.89)	0.94 (0.83–1)	0.77 (0.64–0.92)	0.28 (0.061–1.25)	0.095
	Total	29	21	0.867	4	0	26/46 (57%)	20/46 (43%)	0.447	0.7 (0.57–0.84)	0.63 (0.48–0.83)	0.95 (0.86–1)	0.76 (0.65–0.89)	0.22 (0.049–1)	0.05
Ampullary carcinoma N = 40	No complication	8	11	··	1	0	5	13	··	0.67 (0.49–0·93)	0.8 (0.52–1)	0.69 (0.48–0.99)	0.72 (0.54–0.96)	3.61 (0.698–18.618)	0.126
	Complication	9	12	··	0	0	13	8	…	0.76 (0.6–0.97)	0.92 (0.79–1)	0.75 (0.5–1)	0.86 (0.72–1)	2.23 (0.314–15.872)	0.422
	Total	17	23	1·00	1	0	18/39 (46%)	21/39 (54%)	0.070	0.72 (0.59–0.87)	0.89 (0.75–1)	0.71 (0.54–0.94)	0.79 (0.68–0.93)	3.07 (0.636–14.78)	0.163
Duodenal carcinoma N = 9	No complication	0	0	··	0	0	0	0	··	··	··	··	··	··	··
	Complication	1	8	··	0	0	3	6	··	0.78 (0.55–1)	1 (1–1)	1 (1–1)	1 (1–1)	1 (1–1)	Unobt.
	Total	1	8	1	0	0	3/9 (33%)	6/9 (67%)	1.00	0.78 (0.55–1)	1 (1–1)	1 (1–1)	1 (1–1)	1 (1–1)	Unobt.
Other**—**malignant N = 20^*^	No Complication	1	6	··	0	0	4	3	··	0.57 (0.3–1)	1 (1–1)	1 (1–1)	1 (1–1)	0 (0–Inf)	0.999
	Complication	5	7	··	2	0	7	3	··	0.8 (0.59–1)	0.78 (0.55–1)	1 (1–1)	0.83 (0.65–1)	1.04 (0.106–10.217)	0.973
	Total	6	13	0.381	2	1	11/17 (65%)	6/17 (35%)	0·644	0.71 (0.52–0·96)	0.85 (0.67–1)	1 (1–1)	0.89 (0.77–1)	0.63 (0.065–6.083)	0.69

^*^One patient does not have complications data (1 other patient who had no adjuvant therapy). Bold indicates significant results.

inf indicates infinity; Unobt., unobtainable.

**TABLE 3.** The Association Between Survival, Complications, and Adjuvant Therapy

		No Complications N = 58	Any Complication N = 101	OR/HR (95%CI)	*P*	No POPF N = 128	Any POPF N = 31	OR/HR (95%CI)	No vs Any POPF *P*	Grade A POPF N = 17	CR-POPF N = 14	OR/HR (95%CI)	Grade A vs CR-POPF *P*
Adjuvant treatment received	No N (%)	19 (33%)	42 (42%)	··	··	47 (37%)	14 (45%)	··	··	7 (41%)	7 (50%)	··	··
	Yes N (%)	39 (67%)	59 (58%)	0.69 (0.33–1.41)	0.351	81 (63%)	17 (55%)	0.71 (0.30–1.70)	0.508	10 (59%)	7 (50%)	0.71 (0.13, 3.62)	0.897
Time to start adjuvant treatment in months (95% CI)		2.185 (1.815–2.62)	2.546 (2.135–3.08)	0.68 (0.45–1.01)	0.060	2.3 (1.955–2.907)	2.628 (2.004–3.909)	0.71 (0.42–1.20)	0.197	2.628 (2.267–3.367)	3.679 (1.889–4.484)	0.77 (0.29–2.04)	0.883
Overall survival at 12 months, Rate (95% CI)		0.83 (0.74–0.91)	0.82 (0.76–0.89)	1.28 (0.60–2.71)	0.527	0.81 (0.75–0.87)	0.86 (0.78–0.96)	0.96 (0.39–2.34)	0.926	0.90 (0.89–1.00)	0.81 (0.67–0.98)	0.66 (0.12–3.62)	0.633


In patients specifically with PDAC, complications did not affect the rate of adjuvant treatment given; 29 (73%) of 40 patients with complications compared with 22 (65%) of 35 patients without complications. Those without complications who had adjuvant therapy survival might be more compared to those who did not have adjuvant therapy, survival was not significantly different between those who had adjuvant therapy and no adjuvant therapy if complications occurred (Table 2). Overall, there were no significant associations between survival, complications, and adjuvant therapy in the 159 eligible patients for adjuvant therapy (Table 3). There were no statistically significant differences, between the groups, in terms of quality of life using either the EQ5D or EORTC-QCQ30 instruments (Supplemental Material A, http://links.lww.com/AOSO/A163).

## DISCUSSION

No significant difference between the 2 types of pancreatic anastomosis in terms of the primary endpoint, postoperative pancreatic fistula, were shown. The overall rate of any POPF was 28 (25%) for the BA group and 32 (26%) for CWA group, while clinically relevant POPF (types B and C) occurred in 13 (12%) and 14 (11%) patients, respectively, which compares favorably with other series.^14,15,20,39^ The clinically relevant POPF rate in this series was 27 (11.4%) of 236 cases which is similar when compared to 752 (13.6%) of 5533 cases collected by the Pancreatic Fistula Study Group.^20^ There were also no significant differences between the 2 types of anastomoses with respect to other specific complications. Moreover, PANasta is consistent with International Study Groups in terms of all POPF and CR-POPF occurrence in soft glands and narrow (<3 mm) pancreatic ducts.^20^ The importance of POPF as the initiator of other postoperative complications was demonstrated by the significant association between any POPF and delayed gastric emptying, and any POPF or clinically relevant POPF with POPF volume, and prolonged postoperative hospital stay. Postoperative hemorrhage was recorded as 18% overall; 21% in BA versus 15% in CWA (mean difference [95% CI] 0.66 [0.34–1.29]), *P* = 0.225. There were 6 type B hemorrhages (BA = 4 and CWA = 2) and one further patient in the CWA arm who had a type C hemorrhage associated with a type C POPF and died. We could not show predictability of the FRS, when applied to the current series. The systematic employment of the 2 POPF mitigation procedures (internal pancreatic duct stent and octreotide) may account for this. PANasta recruitment coincided with that of ESPAC-5 (both CRUK funded), therefore a decision was taken that the straight to surgery group in ESPAC-5 could enroll into PANasta, but those undergoing systemic neo-adjuvant treatment would not, primarily to maintain ESPAC-5 recruitment. Another important consideration was that the healing properties of reconstructed pancreata following either neo-adjuvant chemotherapy and or radiotherapy will be completely different. Thus, the assessment of POPF in such cases cannot be purely attributed to the anastomosis technique.

Attention to standardization, (non-neo-adjuvant treatment, pancreatic duct stents, and octreotide use) means that bias is minimized, when measuring the primary end point of POPF, and as a consequence it should be possible to compare the two different anastomoses without confounders. Of the 235 patients randomized with known histology 194 (82.5%) had malignancy and 41 (17.5%) had a benign histology, including 15 (6%) patients with intraductal papillary mucinous neoplasm at the risk of developing invasive cancer in whom surgery was recommended. The remaining 26 (11%) patients had suspicious lesions, but were found to be benign on histology, which is in line with documented series.^26^

To place this study in context, before PANasta; meta-analysis (Supplemental Material A, http://links.lww.com/AOSO/A163) of available evidence (n = 975)^22–24,39–41^ with 40 of 489 (8%) CR-POPF for BA and 80 of 486 (16.5%) for CWA shows superiority for BA (relative risk [RR] 0.49, 95% CI 0.26–0.94). Only one of these studies, Hirono et al^39^ is a randomized study, but is single center, unmasked and has fewer malignant cases. It reports a CR-POPF rate of 6.8% in the CWA arm and 10.3% in the BA arm, which is consistent with PANasta. Further meta-analysis restricted to randomized evidence, PANasta and Hirono^39^ (n = 446) with 24 of 219 (11%) CR-POPF for BA and 21 of 227 (9%) for CWA confirms no advantage (relative risk [RR] 1.19, 95% CI 0.68–2.08).

The main driver of our trial was the attempt to demonstrate that by reducing postoperative complications the proportion of patients with malignancy eligible for adjuvant therapy could be increased and thereby an increase in overall survival. The start point for survival was based on randomization at the time of surgery with no statistically significant evidence that postoperative complications impacted on the delivery of adjuvant therapy, nor on overall survival. The 78% of patients with PDAC that received adjuvant chemotherapy, compares to the 77% reported by the American College of Surgeons study.^13^ ESPAC-1, -3, and -4 adjuvant trials also show no significant association between postoperative complications on overall survival, although in these trials the start point for survival was based on randomization at 2–12 weeks after surgery.^5,6,12^ However, it is suggested in patients with PDAC who had complications, overall survival was decreased.

### Limitations

The multiple malignant pathologies and their distinct prognoses with follow-up restricted to 12 months following randomization limits the generalizability of the long-term survival analysis presented here. Although all patients that were discharged with a POPF or a POPF-driven complication had completely resolved by month 3 of follow-up, it is unclear whether these initial complications had an effect on longer-term survival after 12 months. This is particularly pertinent in those with a malignant diagnosis. Survival of these patients must also be taken in context with 15 patients (8.5%) having missing data on adjuvant chemotherapy.

## CONCLUSIONS

The BA technique did not contribute to a reduction in complications compared to the CWA and there was no statistical association between the development of complications and the delivery of adjuvant therapy as well as overall survival.

## ACKNOWLEDGMENTS

This multicenter trial was funded by a grant from Cancer Research UK (CRUK/13/019). We thank the staff of all participating centers for their outstanding engagement and support of the trial. We also thank the nursing staff and clinical partners who were not directly involved in the conduct of this trial but without whom successful completion of the trial would not have been possible. C.M.H., J.P.N., and P.G. conceived and designed the trial. C.M.H., P.G., R.J., and K.P. supervised trial conduct, participated in data analysis and interpretation, and prepared and wrote the report. K.P. managed the trial and contributed to writing of the manuscript. C.M.H., J.P.N., S.R., D.G., D.O.R., A.S., A.P., B.D., and P.G. participated in patient recruitment and trial conduct. K.P. was responsible for onsite monitoring. R.J. and E.P. participated in trial design, data analysis, and data interpretation. T.M.P. provided independent critical review of the data and drafts. All authors have proof-read the final manuscript.

## Supplementary Material



## References

[1] Kleeff J Korc M Apte M. Pancreatic cancer. Nat Rev Dis Primers. 2016;2:16022. 27158978 10.1038/nrdp.2016.22

[2] [online] G. Cancer incidence and mortality worldwide: International Agency for Research on Cancer Available at: http://globocan.iarc.fr. Accessed January 26, 2022.

[3] Siegel RL Miller KD Fuchs HE. Cancer statistics, 2021. CA Cancer J Clin. 2021;71:7–33. 33433946 10.3322/caac.21654

[4] Conroy T Hammel P Hebbar M; Canadian Cancer Trials Group and the Unicancer-GI–PRODIGE Group. FOLFIRINOX or gemcitabine as adjuvant therapy for pancreatic cancer. N Engl J Med. 2018;379:2395–2406. 30575490 10.1056/NEJMoa1809775

[5] Neoptolemos JP Stocken DD Friess H; European Study Group for Pancreatic Cancer. A randomized trial of chemoradiotherapy and chemotherapy after resection of pancreatic cancer. N Engl J Med. 2004;350:1200–1210. 15028824 10.1056/NEJMoa032295

[6] Neoptolemos JP Palmer DH Ghaneh P; European Study Group for Pancreatic Cancer. Comparison of adjuvant gemcitabine and capecitabine with gemcitabine monotherapy in patients with resected pancreatic cancer (ESPAC-4): a multicentre, open-label, randomised, phase 3 trial. Lancet. 2017;389:1011–1024. 28129987 10.1016/S0140-6736(16)32409-6

[7] Tempero MA Malafa MP Al-Hawary M. Pancreatic adenocarcinoma, Version 2.2021, NCCN Clinical Practice Guidelines in Oncology. J Natl Compr Canc Netw. 2021;19:439–457. 33845462 10.6004/jnccn.2021.0017

[8] Hackert T Sachsenmaier M Hinz U. Locally advanced pancreatic cancer: neoadjuvant therapy with folfirinox results in resectability in 60% of the patients. Ann Surg. 2016;264:457–463. 27355262 10.1097/SLA.0000000000001850

[9] Versteijne E Suker M Groothuis K; Dutch Pancreatic Cancer Group. Preoperative chemoradiotherapy versus immediate surgery for resectable and borderline resectable pancreatic cancer: results of the dutch randomized phase III PREOPANC trial. J Clin Oncol. 2020;38:1763–1773. 32105518 10.1200/JCO.19.02274 PMC8265386

[10] Ghaneh P Palmer DH Cicconi S. ESPAC-5F: Four-arm, prospective, multicenter, international randomized phase II trial of immediate surgery compared with neoadjuvant gemcitabine plus capecitabine (GEMCAP) or FOLFIRINOX or chemoradiotherapy (CRT) in patients with borderline resectable pancreatic cancer. J Clin Oncol. 2020;38:4505. 38.

[11] Katz MHG Shi Q Meyers JP. Preoperative mFOLFIRINOX or mFOLFIRINOX plus hypofractionated radiation therapy (RT) for borderline resectable (BR) adenocarcinoma of the pancreas. J Clin Oncol. 2021;2021:377.

[12] Neoptolemos JP Stocken DD Bassi C; European Study Group for Pancreatic Cancer. Adjuvant chemotherapy with fluorouracil plus folinic acid vs gemcitabine following pancreatic cancer resection: a randomized controlled trial. JAMA. 2010;304:1073–1081. 20823433 10.1001/jama.2010.1275

[13] Merkow RP Bilimoria KY Tomlinson JS. Postoperative complications reduce adjuvant chemotherapy use in resectable pancreatic cancer. Ann Surg. 2014;260:372–377. 24374509 10.1097/SLA.0000000000000378

[14] Bassi C Dervenis C Butturini G; International Study Group on Pancreatic Fistula Definition. Postoperative pancreatic fistula: an international study group (ISGPF) definition. Surgery. 2005;138:8–13. 16003309 10.1016/j.surg.2005.05.001

[15] Bassi C Marchegiani G Dervenis C; International Study Group on Pancreatic Surgery (ISGPS). The 2016 update of the International Study Group (ISGPS) definition and grading of postoperative pancreatic fistula: 11 years after. Surgery. 2017;161:584–591. 28040257 10.1016/j.surg.2016.11.014

[16] Wente MN Bassi C Dervenis C. Delayed gastric emptying (DGE) after pancreatic surgery: a suggested definition by the International Study Group of Pancreatic Surgery (ISGPS). Surgery. 2007;142:761–768. 17981197 10.1016/j.surg.2007.05.005

[17] Wente MN Veit JA Bassi C. Postpancreatectomy hemorrhage (PPH): an International Study Group of Pancreatic Surgery (ISGPS) definition. Surgery. 2007;142:20–25. 17629996 10.1016/j.surg.2007.02.001

[18] Shrikhande SV Sivasanker M Vollmer CM; International Study Group of Pancreatic Surgery (ISGPS). Pancreatic anastomosis after pancreatoduodenectomy: A position statement by the International Study Group of Pancreatic Surgery (ISGPS). Surgery. 2017;161:1221–1234. 28027816 10.1016/j.surg.2016.11.021

[19] Warren KW Cattell RB. Basic techniques in pancreatic surgery. Surg Clin North Am. 1956;36:707–724. 13324659 10.1016/s0039-6109(16)34896-4

[20] Schuh F Mihaljevic AL Probst P. A simple classification of pancreatic duct size and texture predicts postoperative pancreatic fistula: a classification of the International Study Group of Pancreatic Surgery (ISGPS) [Online ahead of print]. Ann Surg. 2021. 10.1097/SLA.0000000000004855 PMC9891297 33914473

[21] Grobmyer SR Kooby D Blumgart LH. Novel pancreaticojejunostomy with a low rate of anastomotic failure-related complications. J Am Coll Surg. 2010;210:54–59. 20123332 10.1016/j.jamcollsurg.2009.09.020

[22] Kleespies A Rentsch M Seeliger H. Blumgart anastomosis for pancreaticojejunostomy minimizes severe complications after pancreatic head resection. Br J Surg. 2009;96:741–750. 19526614 10.1002/bjs.6634

[23] Menonna F Napoli N Kauffmann EF. Additional modifications to the Blumgart pancreaticojejunostomy: results of a propensity score-matched analysis versus Cattel-Warren pancreaticojejunostomy. Surgery. 2021;169:954–962. 32958267 10.1016/j.surg.2020.08.013

[24] Casadei R Ricci C Ingaldi C. Comparison of blumgart anastomosis with duct-to-mucosa anastomosis and invagination pancreaticojejunostomy after pancreaticoduodenectomy: a single-center propensity score matching analysis. J Gastrointest Surg. 2021;25:411–420. 31997074 10.1007/s11605-020-04528-3

[25] Halloran CM Platt K Gerard A. PANasta Trial; Cattell Warren versus Blumgart techniques of panreatico-jejunostomy following pancreato-duodenectomy: study protocol for a randomized controlled trial. Trials. 2016;17:30. 26772736 10.1186/s13063-015-1144-9 PMC4714471

[26] Asbun HJ Conlon K Fernandez-Cruz L; International Study Group of Pancreatic Surgery. When to perform a pancreatoduodenectomy in the absence of positive histology? A consensus statement by the International Study Group of Pancreatic Surgery. Surgery. 2014;155:887–892. 24661765 10.1016/j.surg.2013.12.032

[27] Neoptolemos JP Moore MJ Cox TF; European Study Group for Pancreatic Cancer. Effect of adjuvant chemotherapy with fluorouracil plus folinic acid or gemcitabine vs observation on survival in patients with resected periampullary adenocarcinoma: the ESPAC-3 periampullary cancer randomized trial. JAMA. 2012;308:147–156. 22782416 10.1001/jama.2012.7352

[28] Primrose JN Fox RP Palmer DH; BILCAP Study Group. Capecitabine compared with observation in resected biliary tract cancer (BILCAP): a randomised, controlled, multicentre, phase 3 study. Lancet Oncol. 2019;20:663–673. 30922733 10.1016/S1470-2045(18)30915-X

[29] Meijer LL Alberga AJ de Bakker JK. Outcomes and treatment options for duodenal adenocarcinoma: a systematic review and meta-analysis. Ann Surg Oncol. 2018;25:2681–2692. 29946997 10.1245/s10434-018-6567-6 PMC6097725

[30] Blencowe N Boddy A Harris A. Accounting for intervention complexity in rcts in surgery: new approaches for intervention definition and methods for monitoring fidelity. Trials. 2013;14:O86.

[31] Blencowe N Mills N Whiting P. Providing adequate and practical descriptions in surgical trials. BMJ. 2013;347:f6143. 24129377 10.1136/bmj.f6143

[32] Warren KW Cattell RB Blackburn JP. A long-term appraisal of pancreaticoduodenal resection for peri-ampullary carcinoma. Ann Surg. 1962;155:653–662. 14005049 10.1097/00000658-196205000-00004 PMC1466138

[33] Dindo D Demartines N Clavien PA. Classification of surgical complications: a new proposal with evaluation in a cohort of 6336 patients and results of a survey. Ann Surg. 2004;240:205–213. 15273542 10.1097/01.sla.0000133083.54934.ae PMC1360123

[34] O’Brien PC Fleming TR. A multiple testing procedure for clinical trials. Biometrics. 1979;35:549–556. 497341

[35] Kaplan EL Meier P. Nonparametric estimation from incomplete observations. J Am Stat Assoc. 1958;53:457–481.

[36] Mantel N. Evaluation of survival data and two new rank order statistics arising in its consideration. Cancer Chemother Rep. 1966;50:163–170. 5910392

[37] Mungroop TH van Rijssen LB van Klaveren D. Alternative fistula risk score for pancreatoduodenectomy (a-FRS). Ann Surg. 2017;269:937–943. 10.1097/SLA.0000000000002620 29240007

[38] Steyerberg EW Vickers AJ Cook NR. Assessing the performance of prediction models: a framework for traditional and novel measures. Epidemiology. 2010;21:128–138. 20010215 10.1097/EDE.0b013e3181c30fb2 PMC3575184

[39] Hirono S Kawai M Okada KI. Modified blumgart mattress suture versus conventional interrupted suture in pancreaticojejunostomy during pancreaticoduodenectomy: randomized controlled trial. Ann Surg. 2019;269:243–251. 29697455 10.1097/SLA.0000000000002802 PMC6325750

[40] Kojima T Niguma T Watanabe N. Modified Blumgart anastomosis with the “complete packing method” reduces the incidence of pancreatic fistula and complications after resection of the head of the pancreas. Am J Surg. 2018;216:941–948. 29606278 10.1016/j.amjsurg.2018.03.024

[41] Lee YN Kim WY. Comparison of Blumgart versus conventional duct-to-mucosa anastomosis for pancreaticojejunostomy after pancreaticoduodenectomy. Ann Hepatobiliary Pancreat Surg. 2018;22:253–260. 30215047 10.14701/ahbps.2018.22.3.253 PMC6125278

