# Supplementary material for: A Multicenter, Randomized, Double-Blinded, Clinical Trial Comparing Cattell-Warren and Blumgart Anastomoses Following Partial Pancreatoduodenectomy: PANasta Trial
Source: Ann Surg Open. 2022 Sep 15;3(3):e198. doi: 10.1097/AS9.0000000000000198 (PMC9508971; doi:10.1097/AS9.0000000000000198)
Supplement: Supplementary file 1 [file as9-3-e198-s001.pdf]

Supplementary Table 1. Patient demographics, surgical and pathology findings

	**Blumgart (BA) (n=112)**	**Cattell Warren (CWA) (n=124)**	**Total (n=236)**
**Age in years median (IQR)**	70 (61·5 to 74)	70 (62·5 to 76)	70 (62 to 75)
**Gender** *N* **(%)**			
Female	42 (38)	45 (36)	87 (37)
Male	70 (63)	79 (64)	149 (63)
**Days to surgery since enrolment** Median (IQR)	1 (0 to 2)	1 (0 to 1·5)	1 (0 to 2)
**Days to surgery since date of suspected cancer diagnosis** Median (IQR)	33 (20 to 49)	33 (21 to 48·5)	33 (20 to 49)
**Smoking status** *N* (%)			
Ever	73 (65)	74 (60)	147 (62)
Never	39 (35)	50 (40)	89 (38)
**Alcohol status** *N* (%)			
None	40 (36)	34 (27)	74 (31)
Sporadic	43 (38)	51 (41)	94 (40)
Regular	20 (18)	31 (25)	51 (22)
Excessive	9 (8)	6 (5)	15 (6)
Missing	0 (0)	2 (1)	2 (1)
**Diabetic status** *N* (%)			
None	93 (83)	98 (79)	191 (81)
Type 1	0 (0)	5 (4)	5 (2)
Type 2	14 (13)	14 (11)	28 (12)
Type 2 (on insulin)	4 (4)	7 (6)	11 (4·6)
Missing	1 (0)	0 (0)	1 (0·4)
**Pancreatic endocrine insufficiency** *N* (%)			
No (not requiring treatment)	2 (2)	1 (1)	3 (1)
Yes (requiring treatment)	63 (27)	46 (19)	109 (46)
Missing	47 (20)	77 (33)	124 (53)
**Surgical and pathology findings**			
**Resection Type** *N* (%)		(1 missing)	235

Pylorus preserving partial pancreato-duodenectomy	96 (86)	106 (85)	202 (86)
Kausch Whipple partial pancreato-duodenectomy	14 (12·5)	16 (13)	30 (12·8)
Pylorus preserving partial pancreato-duodenectomy + other viscera	2 (2)	1 (1)	3 (1·2)
**Histology** *N* **(%)**		(1 missing)	235
**Malignant**	**92 (82)**	**102 (82)**	**194 (82·5)**
Pancreatic ductal adenocarcinoma	38 (34)	37 (30)	75 (32)
Cholangiocarcinoma	29 (26)	21 (17)	50 (21)
Ampullary adenocarcinoma	17 (15)	23 (19)	40 (17)
Duodenal adenocarcinoma	1 (1)	8 (6)	9 (4)
Neuroendocrine carcinoma	2 (2)	5 (4)	7 (3)
Other[a]	5 (4)	8 (6·5)	13 (5·5)
**Non-malignant**	**20 (18)**	**21 (17)**	**41 (17·5)**
Intraductal papillary mucinous neoplasm (IPMN)	6 (5)	9 (7)	15 (6)
Adenoma	4 (3·5)[b]	5 (4)	9 (4)
Pancreatitis (chronic)	1 (1)	2 (1·5)	3 (1)
Pancreatitis[c] (other)	2 (2)	2 (1·5)	4 (2)
Other[d]	7 (6)	3 (2)	10 (4·5)

[a]Malignant other: BA arm; solid pseudo-papillary tumour (n=1), colloid carcinoma + biliary intraductal papillary mucinous neoplasm (n=1), metastatic colorectal cancer (n=1), metastatic renal cell carcinoma (n=1), and pancreatic neuroendocrine tumour + ampullary adenoma (n=1). CWA arm; adenosquamous carcinoma (n=3), pancreatic neuroendocrine tumour (n=2), solid pseudo-papillary tumour (n=1), gastric adenocarcinoma (n=1), and colloid carcinoma + mixed intraductal papillary mucinous neoplasm (n=1).

[b]1 duodenal adenoma with endocrine microadenoma.

[c]BA group; necrotising granulomatous pancreatitis (1) and Xanthogranulomatous pancreatitis (1). CWA group; autoimmune pancreatitis (2).

[d]Non-malignant other: BA arm; biliary intraductal papillary mucinous neoplasm (n=1), Brunner's gland hyperplasia (n=1), choledochal cyst(n=1), inflammation (n=1), borderline intraductal papillary mucinous neoplasm (n=1), lymphoepithelial cyst (n=1) and lipomatosis (n=1). CWA arm; intra-ampullary papillary-tubular neoplasm (n=1), duodenal leiomyoma (n=1) and ampullary ulcer (n=1).

Supplementary Table 2: Post-operative outcomes

	**Blumgart (n=112)**	**Cattell -Warren (n=124)**	**Total (n=236)**	**Odds Ratio[a] (95% CI)**	***P* value**
**Post-Operative Pancreatic Fistula (POPF)**					
No fistula. *N* (%)	84 (75)	92 (74)	176 (75)	··	··
Any POPF. *N* (%)	28 (25)	32 (26)	60 (25)	··	··
Overall Rate of POPF (95% CI)	0·25 (0·17, 0·38)	0·26 (0·18, 0·34)	0·25(0·20, 0·32)se	1·04 (0·58, 1·88)	0·887
Type A. *N* (%)	15 (6)	18 (8)	33 (14)		
Odds rate (95% CI)	*0·13 (0·07 - 0·2)*	*0·15 (0·08 - 0·21)*	*0·14 (0·10,0·19)*	*1·1 (0·52 - 2·3)*	*0·804*
Type B. *N* (%)	10 (4)	12 (5)	22 (9)		
Odds rate (95% CI)	*0·09 (0·04 - 0·14)*	*0·1 (0·04 - 0·15)*	*0·09 (0·06, 0·14)*	*1·09 (0·45 - 2·64)*	*0·843*
Type C. *N* (%)	3 (1·5)	2 (0·5)	5 (2)		
Odds rate (95% CI)	*0·03 (0 - 0·06)*	*0·02 (0·0 - 0·06)*	*0·02 (0·01, 0·05)*	*0·6 (0·1 - 3·63)*	*0·574*
**Pancreatic Consistency**					
Hard.	67 (60)	67 (54)	134 (57)	··	··
No Fistula. *N* (%)	59 (88)	62 (93)	121 (90)	··	··
Total number of POPF (%)	8 (12)	5 (7)	13 (10)	··	··
Overall POPF Rate. (95% CI)	*0·12 (0·04, 0·20)*	*0·07 (0·01, 0·14)*	*0·1 (0·5, 0·16)*	*0·59 (0·18 – 1·92)*	*0·385*
Type B/C POPF Rate. (95% CI)	*0·04 (0 - 0·09)*	*0·04 (0 - 0·09)*	*0·04 (0·01,0·09)*	*1 (0·19 - 5·14)*	*1*
Soft / Normal.	45 (40)	57 (46)	102 (43)	··	··
No Fistula. *N* (%)	25 (56)	30 (53)	55 (54)	··	··
Total number of POPF (%)	20 (44)	27 (47)	47 (46)	··	··
Overall POPF Rate. (95% CI)	*0·44 (0·30, 0·59)*	*0·47 (0·34, 0·6)*	*0·46 (0·36, 0·56)*	*1·12 (0·51 – 2·47)*	*0·769*
Type B/C POPF Rate. (95% CI)	*0·16 (0·05 - 0·26)*	*0·16 (0·06 - 0·25)*	*0·16 (0·1 – 0·24)*	*1·02 (0·35 - 2·98)*	*0·974*
**Pancreatic duct diameter**					
Dilated (>3mm)·	40 (36)	49 (40)	89 (38)	··	··
No Fistula. *N* (%)	34 (85)	42 (86)	76 (85)	··	··
Total number of POPF (%)	6 (15)	7 (14)	13 (15)	··	··
Overall POPF Rate. (95% CI)	*0·15 (0·04, 0·26)*	*0·14 (0·04, 0·24)*	*0·14 (0·08, 0·24)*	*0·94 (0·29 – 3·07)*	*0·924*
Type B/C POPF Rate. (95% CI)	*0·02 (0 - 0·15)*	*0·02 (0 - 0·12)*	*0·02 (0, 0·09)*	*0·81 (0·05 - 13·41)*	*0·885*
Non-Dilated (≤3mm).	72 (64)	75 (60)	147 (62)	··	··
No Fistula. *N* (%)	50 (69)	50 (67)	100 (68)	··	··
Total number of events	22 (31)	25 (33)	47 (32)	··	··
Overall POPF Rate. (95% CI)	*0·31 (0·2, 0·41)*	*0·33 (0·23, 0·44)*	*0·32 (0·25, 0·40)*	*1·14 (0·57 – 2·28)*	*0·718*
Type B/C POPF Rate. (95% CI)	*0·12 (0·05 - 0·2)*	*0·15 (0·07 - 0·23)*	*0·14 (0·10, 0·21)*	*1·2 (0·47 - 3·1)*	*0·702*
**Secondary outcome: specified complications**					
Intra-operative haemorrhage. median (IQR) ml	560<br>(400, 900)	600<br>(453·75, 903·75)	600<br>(400, 900)	Mean difference (95% CI)<br>-17 (-151, 118)	0·807
Postoperative haemorrhage. *N* (%)	24 (21)	19 (15)	43 (18)	0·66 (0·34 – 1·29)	0·225
Delayed gastric emptying. *N* (%)[b]	34 (30)	40 (32)	74 (31)	1·09 (0·63 – 1·9)	0·756
Wound infection. *N* (%)	24 (21)	19 (15)	43 (18)	0·66 (0·34 – 1·29)	0·25
Pulmonary infection. *N* (%)	14 (12)	8 (6)	22 (9)	0·48 (0·19 – 1·2)	0·116

Duration of Surgery<br>Median (IQR) hours.	6·96 (6, 7·75)	6·87 (6, 8·42)	6·92 (6, 8)	Mean difference log scale (95% CI)<br>-0·25 (-0·67, 0·18)	0·431
Intra-abdominal post-operative fluid collection maximum diameter. median (IQR) mm	238<br>(52, 774)	237<br>(45, 1130)	238<br>(48, 1006)	Mean difference log scale (95% CI)<br>0·02 (-0·60, 0·56)	0·954
Re-operation. *N* (%)	11 (10)	6 (5)	17 (7)	0·46 (0·16 – 1·28)	0·136
Venous thrombo-embolism. *N* (%)	7 (6)	3 (2)	10 (4)	0·37 (0·09 – 1·47)	0·159
Postoperative death. *N* (%)	5 (4)	2 (2)	7 (3)	0·35 (0·07 – 1·84)	0·216
**Post-Operative Hospital Stay**					
Mean stay in days. (SD)	18·49 (12·70)	21·11 (19·36)	16·8 (14·65)	Mean difference (95% CI)<br>2·62 (-1·69, 6·94)	0·232
Median stay in days. (IQR)	13 (10 – 24)	14·5 (10 – 22)	12 (9, 1975)		
**Overall Survival - All Patients**					
Total deaths on study.	24 (21)	20 (16)	44 (18·5)	0·71 (0·37 – 1·36)	0·298
Overall survival at 12-months. Hazard Ratio (95% CI)	0·787<br>(0·713, 0·868)	0·854<br>(0·792, 0·921)	0·822<br>(0·773, 0·874)	0 ·72 (0·4, 1·311)	0·266
Deaths from disease progression. *N (%)*	17 (71)	15 (75)	32 (73)	1·30 (0·53,3·20)	0·542
Deaths without disease progression. *N (%)*	7 (29)	5 (25)	12 (27)	1·56 (0·40,6·59)	0·552
**Adjuvant Therapy in Eligible Patients**					
Total eligible. *N*[c]<br>*[missing data]*	85 (76)<br>*[6]*	89 (72)<br>*[9]*	174 (74)<br>*[25]*	0·91 (0·52 - 1·59)	0·749
Total entering adjuvant chemotherapy. *N* (%)	49/79 (62)	49/80 (61)	98/159 (62)	1·00 (0·52, 2·06)	0·618
Median time to start adjuvant chemotherapy. (IQR)	2·27<br>(1·87 – 2·78)	2·46<br>(2·07 – 3·05)	2·33 (1·96, 3·01)	0·84 (0·57, 1·24)	0·379

[a]Unless otherwise specified·

[b]DGE by POPF type· Severity A vs No Fistula 1·86 (0·857, 4·05), p=0·116· Severity B/C vs No Fistula 4·17 (1·801, 9·652), p<0·001·

[c]Eligible here refers to patients with PDAC, Cholangiocarcinoma, Ampullary Adenocarcinoma, and Neuroendocrine Carcinoma· Please note that 6 patients outside of this group also received adjuvant therapy (Table 4a)

Deaths by Tumour type:

- BA: PDAC (n=10); Ampullary (n=3); Cholangiocarcinoma (n=9); Neuroendocrine Carcinoma (n=1); Other-Malignant (n=1)
- CWA: PDAC (n=7); Ampullary (n=6); Cholangiocarcinoma (n=4); Neuroendocrine Carcinoma (n=1); Other-Malignant (n=1); Other, non-malignant (n=1)

Supplementary Table 3. Serious adverse events.

		Blumgart Anastomosis (n=112)	Cattell-Warren Anastomosis (n=124)
**Patients with at least 1 SAE**		15	16
**Number of documented SAE's (events)**		18	21
**Grade**	II	2	1
	III	3	4
	IV	9	12
	V	4	4
**Related to surgery**	No	4	4
	Yes	14	17
**Outcome**	Ongoing	11	13
	Resolved	2	1
	Resolved with sequalae	··	2
	Death / Ongoing at Death	5[a]	5[b]
**Categorisation**		··	··
Acute Kidney Injury		··	1
Biliary Anastomotic Fistula		··	1
Colonic Fistula		··	1
Disease Reoccurrence		··	1
Encephalopathy		··	1
Haematoma		1	··
Injury, Poisoning and procedural Complications (other)		1	··
Interoperative Splenic Injury		1	··
Lung Infection		4	··
Myocardial Infarct		1	··
Nerve Injury		··	1
Post-Operative Pancreatic Fistula		··	2
Pancreatitis		1	··
Pneumothorax		1	··
Post-Operative Haemorrhage		4	3
Respiratory failure		1	4
Sepsis		1	3
Thromboembolic Event		··	1
Ventricular Fibrillation		1	··
Visceral Arterial Ischaemia		1	1
Would Dehiscence		··	1

[a]Five grade V events caused death in five patients in the Blumgart arm: Hospital acquired pneumonia (1 event), Respiratory Failure (1 event), Upper Gastrointestinal Bleed (1 event), Ischaemic Small Bowel (1 event) and Post-Operative bleed secondary to Type C POPF (1 event).

[b]Five events were causative or present at death in three patients in the Cattell-Warrant arm. 1 patient developed liver metastases (1 event). 1 patient developed **both** a Type C POPF, underwent a relaparotomy and then subsequently died (2 events). 1 patient developed both a Type C POPF and a hepato-jejunostomy stricture causing wound dehiscence and liver abscesses, both ongoing at death (2 events).
