# Supplementary material for: A Multicenter, Randomized, Double-Blinded, Clinical Trial Comparing Cattell-Warren and Blumgart Anastomoses Following Partial Pancreatoduodenectomy: PANasta Trial
Source: Ann Surg Open. 2022 Sep 15;3(3):e198. doi: 10.1097/AS9.0000000000000198 (PMC9508971; doi:10.1097/AS9.0000000000000198)
Supplement: Supplementary file 2 [file as9-3-e198-s002.pdf]

SUPPLEMANTARY MATERIAL A: Sequential steps of modified Blumgart and Cattell-Warren anastomoses.

Mandatory preparation of the pancreatic neck following pancreato-duodenectomy.

This stage is common to both anastomotic techniques.

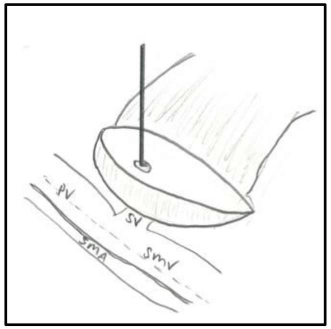

Blumgart Anastomosis (BA).

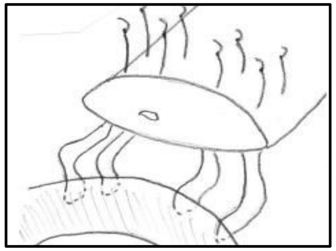

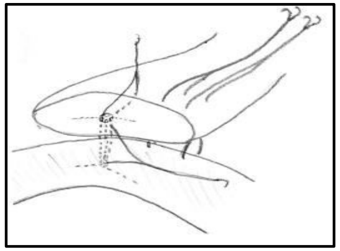

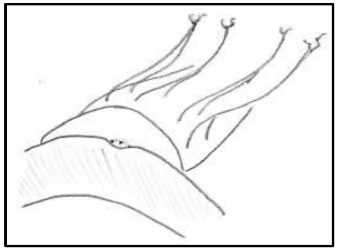

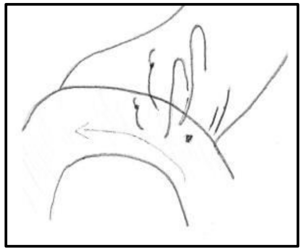

Sequential steps of each anastomosis

Posterior row of pancreatic parenchymal sutures

Pancreatic duct to jejunal mucosa sutures

Posterior parenchymal and duct-to-mucosa sutures have been tied

Completion of the anterior row of pancreatic parenchymal sutures

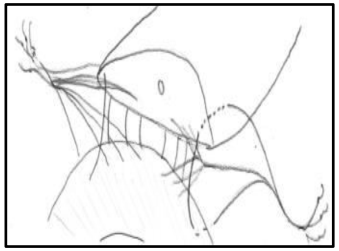

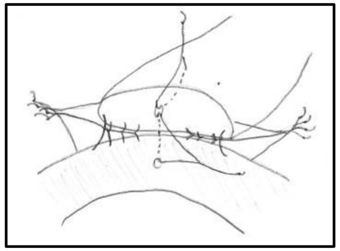

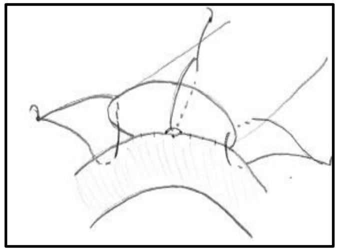

Cattell-Warren Anastomosis (CWA).

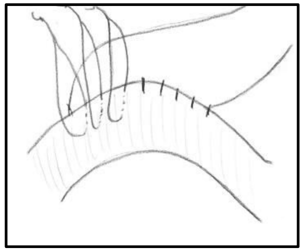

SUPPLEMANTARY MATERIAL A: Intra-Operative Photographs For Quality Assurance

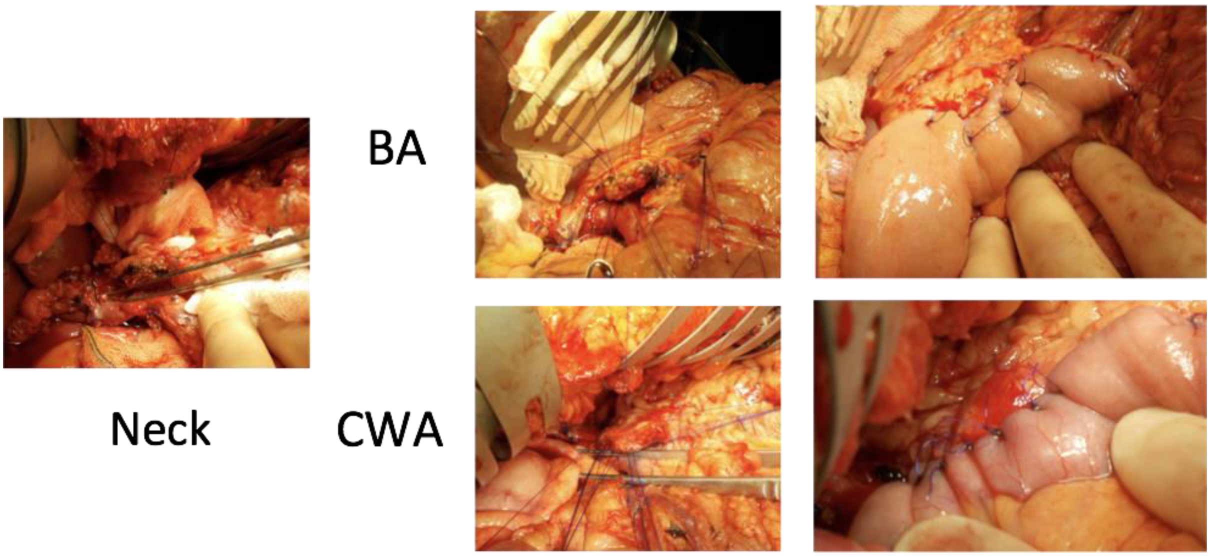

SUPPLEMANTARY MATERIAL A: Pancreatic Risk Score

The Fistula Risk Score based pancreatic texture, main pancreatic duct diameter, and body mass index, when applied to the current series to predict any POPF had an AUC (95% CI), of 0·61 (0·53, 0·68) for any POPF and 0·54 (0·44, 1·64) for CR-POPF

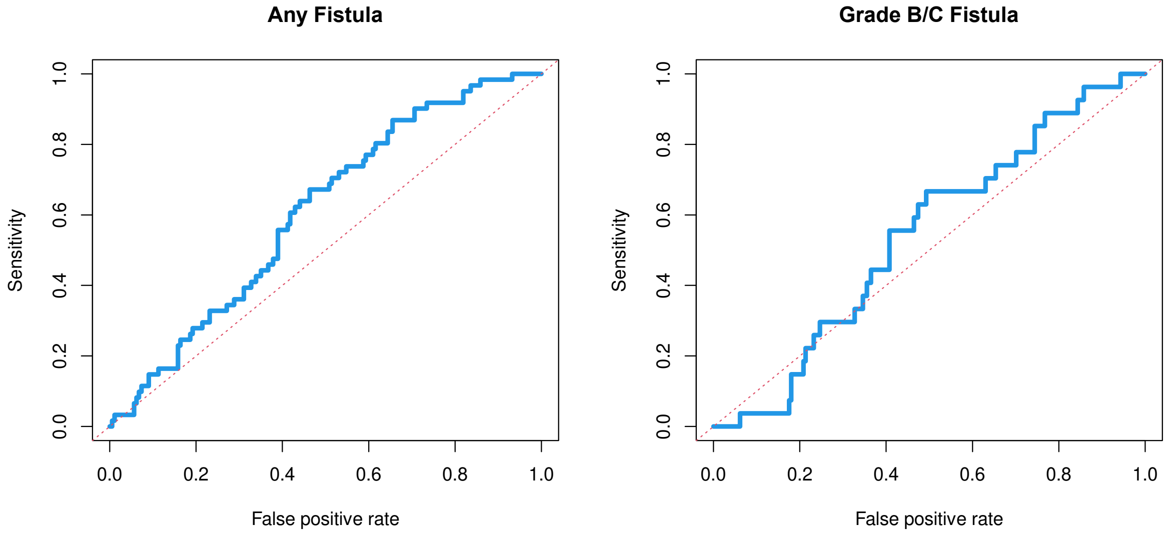

SUPPLEMANTARY MATERIAL A: Quality of Life
Quality of Life is measured using two instruments
- EORTC-QCQ30
- EQ5D

For each of these instruments, the median IQR global health scores are summarised across treatment arm. Summaries are given in terms of median (IQR). An Analysis of Covariance (ANCOVA) analysis is performed using the 12 months QoL scores as the dependent variable and using the registration QoL scores as a covariate

EORTC - QCQ30 Analysis

Table 1. EORTC Global Health Score by Treatment arm

Time Point. Median (IQR)	Blumgart Anastomosis	Cattell-Warren Anastomosis	Total
Registration	66·67 (41·67, 83·3)	66·67 (50, 83·33)	66·67 (50, 83·33)
Post-Op Review Discharge	50 (33·33, 66·67)	50 (33·33, 66·67)	50 (33·33, 66·67)
3 Month Follow Up	66·67 (50, 83·33)	66·67 (50, 83·33)	66·67 (50, 83·33)
6 Month Follow Up	75 (50, 83·33)	66·67 (50, 83·33)	66·67 (50, 83·33)
12 Month Follow Up	83·33 (66·67, 83·33)	75 (58·33, 83·33)	75 (58·33, 83·33)

Table 2 shows that there is no statistically significant difference in the 12-month EORTC-QCQ30 QoL score between the two treatment arms

Table 2. Results of ANCOVA for EORTC-QCQ30 Global Health Scores

Term	Estimate	Standard Error	T-Value	P-value
Intercept	69·06	5·62	12·29	<0·001
Registration Score	0·095	0·074	1·278	0·204
Treatment Arm (Cat. War Vs. Blumgart)	-4·809	3·51	-1·368	**0·174**

EQ5D Analysis

Table 3 shows the median (IQR) global health score by treatment arm as measured by the EQ5D instrument,

Time Point. Median (IQR)	Blumgart Anastomosis	Cattell-Warren Anastomosis	Total
Registration	71 (60, 90)	77 (60, 90)	75 (60, 90)
Post-Op Review Discharge	60 (50, 75)	62.5 (50, 80)	60 (50, 80)
3 Month Follow Up	70 (60, 85)	75 (60, 86)	75 (60, 85)
6 Month Follow Up	76 (70, 85·25)	71·5 (60, 81·25)	75 (6325, 85)
12 Month Follow Up	80 (70, 90)	80 (60·75, 90)	80 (70, 90)

Table 4 shows that there is no statistically significant difference in the 12-month EQ5D QoL score between the two treatment arms.

Table 4. Results of ANCOVA for EQ5D Global Health Scores

Term	Estimate	Standard Error	T-Value	P-value
Intercept	78·64	2·69	29·207	<0·001
Registration Score	-0·004	0·010	-4·20	0·675
Treatment Arm (Cat. War Vs. Blumgart)	-3·149	3·268	-0·964	0·337

SUPPLEMANTARY MATERIAL A: Meta-Analyses.

Studies reporting CR-POPF before PANasta.

Study	BA Events	BA Total	CWA Events	CWA Total	RR	95%-CI	Weight (fixed)	Weight (random)
Kleespies et al (2009)	4	92	12	90	0.33	[0.11; 0.97]	15.2%	15.9%
Kojima et al (2018)	3	101	20	103	0.15	[0.05; 0.50]	24.8%	14.8%
Lee et al (2018)	1	43	6	44	0.17	[0.02; 1.36]	7.4%	7.2%
Casadei et al (2020)	8	37	10	37	0.80	[0.36; 1.80]	12.5%	20.2%
Menonna et al (2020)	13	109	25	109	0.52	[0.28; 0.96]	31.2%	23.4%
Hirono et al (2019)	11	107	7	103	1.51	[0.61; 3.75]	8.9%	18.6%
**Fixed effect model**		**489**		**486**	**0.50**	**[0.35; 0.71]**	**100.0%**	**--**
**Random effects model**					**0.49**	**[0.26; 0.94]**	**--**	**100.0%**

Heterogeneity: $I^2 = 60\%$, $^2 = 0.3573$, $p = 0.03$

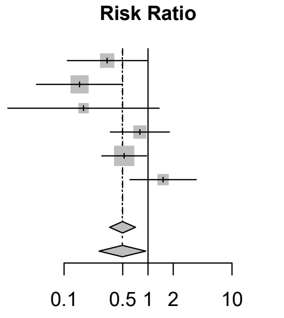

After PANasta, RCTs only. CR-POPF.

Study	BA Events	BA Total	CWA Events	CWA Total	RR	95%-CI	Weight (fixed)	Weight (random)
Hirono et al (2019)	11	107	7	103	1.51	[0.61; 3.75]	34.9%	38.0%
Halloran et al (2022)	13	112	14	124	1.03	[0.51; 2.09]	65.1%	62.0%
**Fixed effect model**		**219**		**227**	**1.20**	**[0.69; 2.09]**	**100.0%**	**--**
**Random effects model**					**1.19**	**[0.68; 2.08]**	**--**	**100.0%**

Heterogeneity: $I^2 = 0\%$, $^2 = 0$, $p = 0.51$

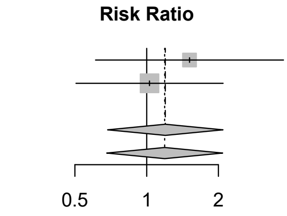

After PANasta, soft pancreas only. CR-POPF.

Study	BA Events	BA Total	CWA Events	CWA Total	RR	95%-CI	Weight (fixed)	Weight (random)
Kleespies et al (2009)	.	.	.	.			0.0%	0.0%
Kojima et al (2018)	3	60	20	45	0.11	[0.04; 0.36]	56.3%	31.0%
Lee et al (2018)	.	.	.	.			0.0%	0.0%
Casadei et al (2020)	.	.	.	.			0.0%	0.0%
Menonna et al (2020)	.	.	.	.			0.0%	0.0%
Hirono et al (2019)	11	61	7	58	1.49	[0.62; 3.59]	17.7%	33.8%
Halloran et al (2022)	11	45	12	57	1.16	[0.57; 2.38]	26.1%	35.3%
**Fixed effect model**		**166**		**160**	**0.63**	**[0.40; 0.98]**	**100.0%**	**--**
**Random effects model**					**0.61**	**[0.15; 2.56]**	**--**	**100.0%**

Heterogeneity: $I^2 = 87\%$, $^2 = 1.3738$, $p < 0.01$

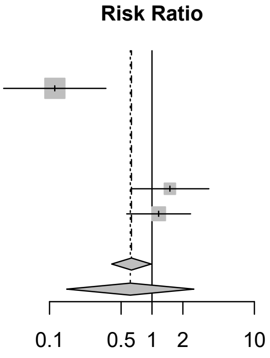

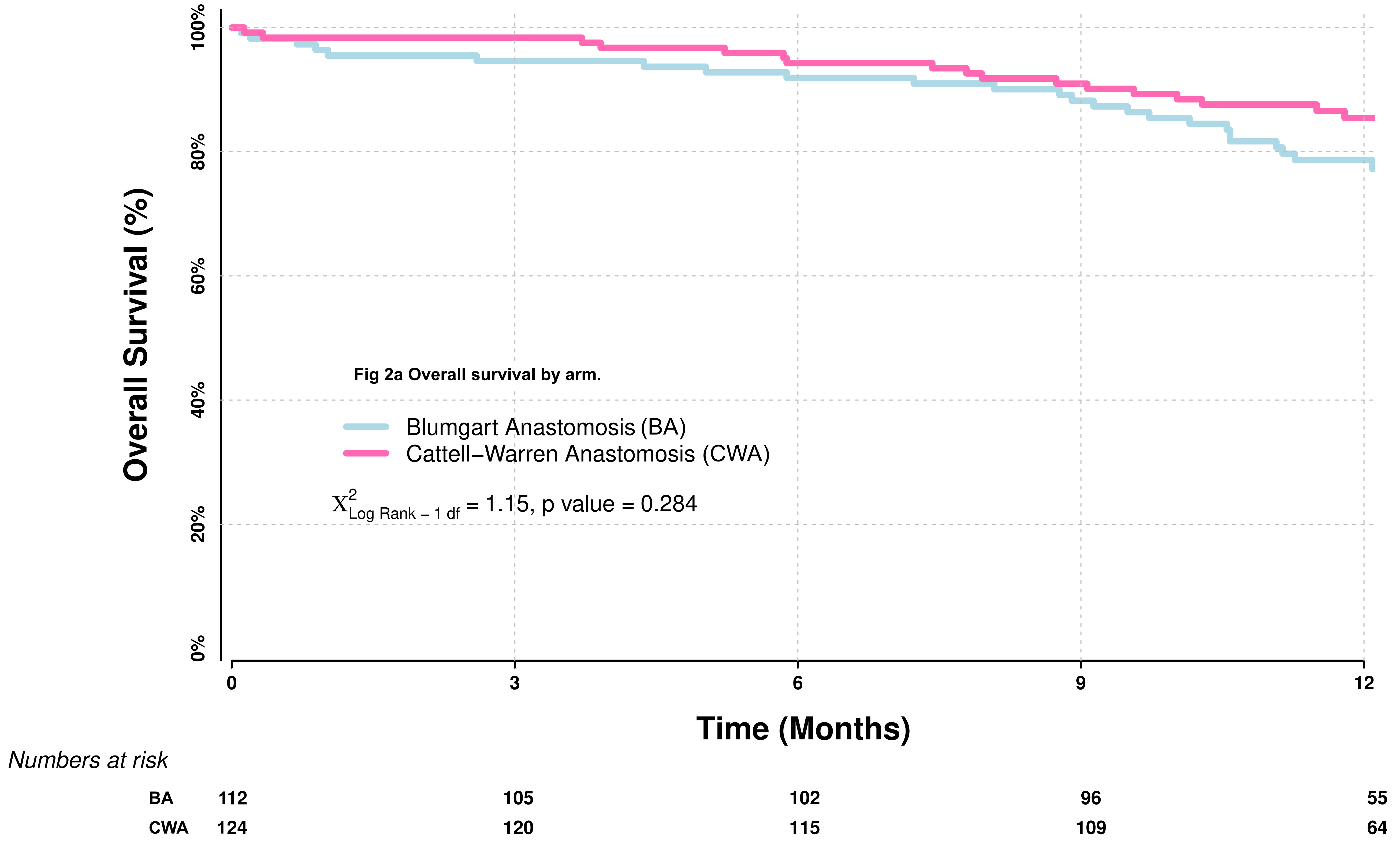
Overall Survival (%)
100%
80%
60%
40%
20%
0%
Fig 2a Overall survival by arm.
Blumgart Anastomosis (BA)
Cattell–Warren Anastomosis (CWA)
$X^2_{\text{Log Rank} - 1\text{ df}}$ = 1.15, p value = 0.284
0
3
6
9
12
Time (Months)
Numbers at risk
BA 112 105 102 96 55
CWA 124 120 115 109 64

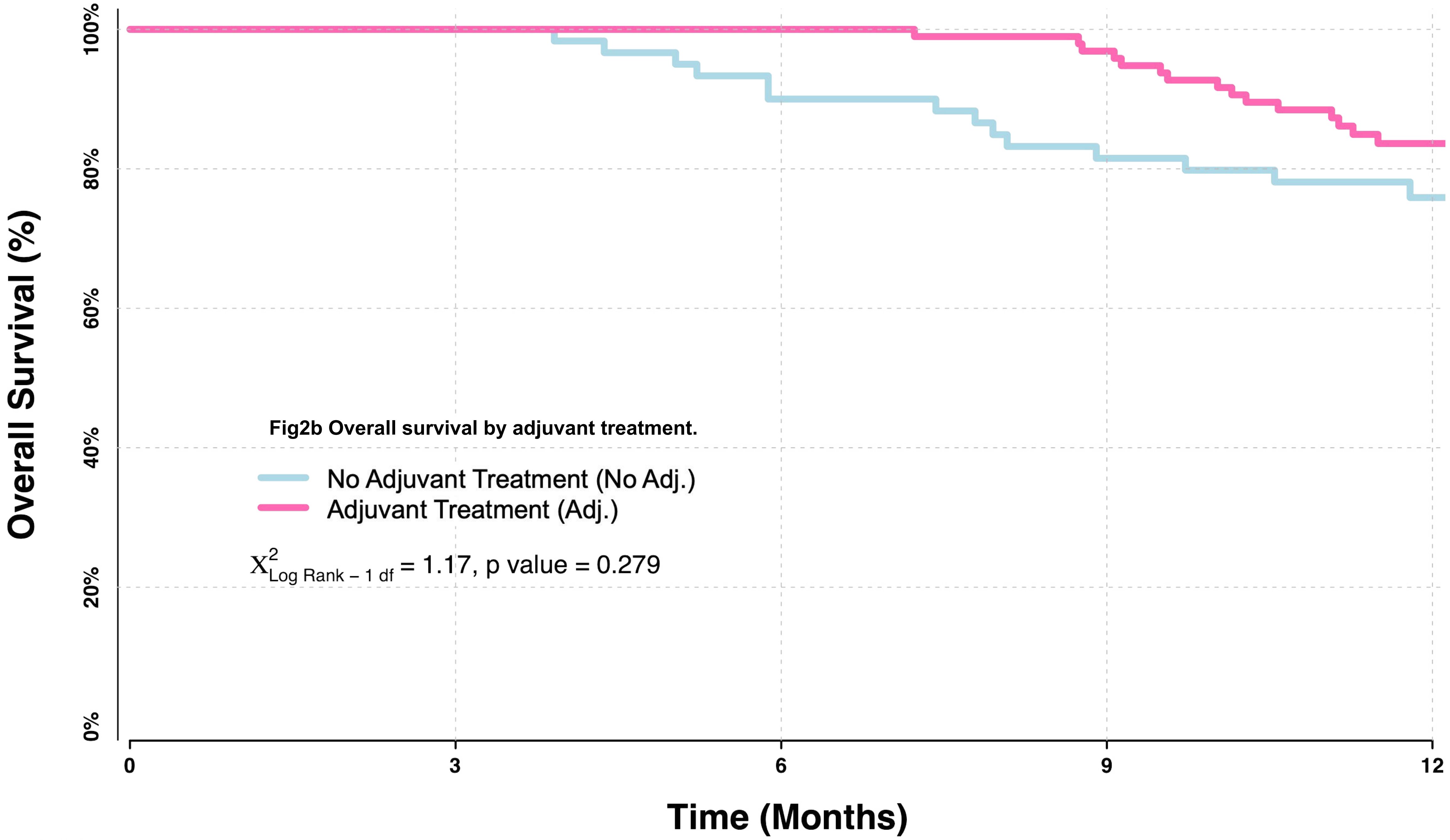

Numbers at risk

No Adj.	**61**	**60**	**54**	**48**	**29**
Adj.	**98**	**98**	**98**	**93**	**49**

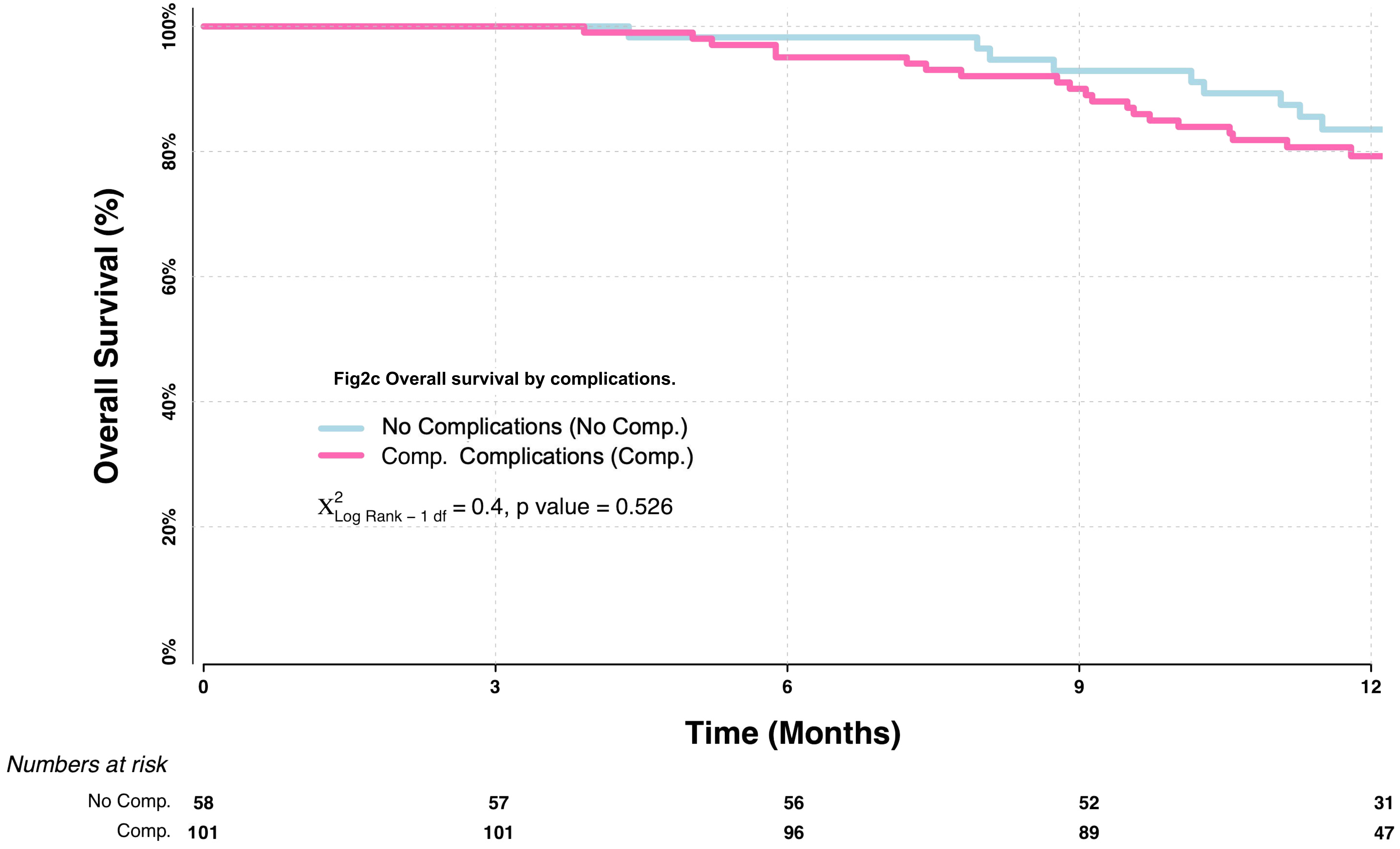
100%
80%
60%
40%
20%
0%
Overall Survival (%)
Fig2c Overall survival by complications.
No Complications (No Comp.)
Comp. Complications (Comp.)
$X^2_{\text{Log Rank} - 1\ \text{df}} = 0.4$, p value = 0.526
0
3
6
9
12
Time (Months)
Numbers at risk
No Comp. 58 57 56 52 31
Comp. 101 101 96 89 47

CONFIDENTIAL

PANasta Trial
Cattell Warren versus Blumgart techniques of pancreatico-jejunostomy following pancreato-duodenectomy – a double blinded multi centred trial

Study Sponsor:

University of Liverpool
Research Support Office
2nd Floor Block D Waterhouse Building
3 Brownlow Street
Liverpool
L69 3GL

**MREC reference**	14/NW/1393
**ISRCTN number:**	ISRCTN52263879
**IRAS reference**	162472
**Sponsor Protocol Code:**	UoL000732
**Protocol version:**	Version 6
**Date:**	12/05/2016

–

Study Protocol Approval

I, the undersigned, hereby approve this clinical study protocol:

Signature: ______________________ **Date:** 19/5/16.

Christopher Halloran
Chief Investigator
Department of Molecular and Clinical Cancer Medicine
Institute of Translational Medicine
University of Liverpool
L69 3GA.
Tel: 0151 706 4087
Fax: 0151 706 5826
E-mail: halloran@liverpool.ac.uk

Authorised on behalf of the Sponsor:

Signature: ______________________ **Date:** 23 MAY 2016

Alex Astor
Head of Research Support – Faculty of Health and Life Sciences
University of Liverpool
2nd Floor Block D Waterhouse Building
3 Brownlow Street
Liverpool
L69 3GL

General Information

This document describes the PANasta trial and provides information about procedures for entering patients into it. The protocol should not be used as an aide-memoir or guide for the treatment of other patients. Every care was taken in its drafting, but corrections or amendments may be necessary. These will be circulated to the registered investigators in the trial, but centres entering patients for the first time are advised to contact the coordinating centre (Cancer Research UK Liverpool Cancer Trials Unit (LCTU)) to confirm they have the most up to date version. Clinical problems relating to this trial should be referred to the relevant Chief Investigator via LCTU.

Statement of Compliance

This study is designed to comply with the guideline developed by the International Conference on Harmonisation (ICH) for Good Clinical Practice (GCP) and will be conducted in compliance with the protocol, LCTU Standard Operating Procedures and EU Directive 2001/20/EC, transposed into UK law as the UK Statutory Instrument 2004 No 1031: Medicines for Human Use (Clinical Trials) Regulations 2004

UK Registration

This study will have National Research Ethics Service (NRES) approval. Each centre must also undergo Site Specific Assessment by the relevant Trust Research and Development department (or Local Research Ethics Committee for Non-NHS Sites) and NHS sites must be granted Research and Development Approval from each Trust where the trial will be carried out.

Contact Details: Institutions

**Sponsor:**	**Trial Management and Monitoring:**	**Clinical Laboratory:**
**University of Liverpool**<br>Research Support Office<br>2nd Floor Block D<br>Waterhouse Building<br>3 Brownlow Street<br>Liverpool<br>L69 3GL<br><br>Tel:+44 (0) 151 794 8379<br>Email:sponsor@liv.ac.uk	**Kellie Platt**<br>Trial Co-ordinator<br>Cancer Research UK:<br>Liverpool Cancer Trials Unit<br>1st Floor, Block C<br>Waterhouse Building<br>1-3 Brownlow Street<br>Liverpool<br>L69 3GL<br><br>Tel: +44 (0) 151 795 5266<br>Fax: + 44 (0) 151 794 8930<br>Email:kellie.platt@liverpool.ac.uk	**Dr William Greenhalf**<br>Liverpool GCLP Facility<br>Department of Molecular and Clinical Medicine<br>5th Floor UCD Block<br>Daulby Street<br>Liverpool<br><br>Tel: +44 (0)151 706 4184<br>Fax: +44 (0) 151 706 5826<br>Email:<br>greenhaf@liverpool.ac.uk
**Pathology:**	**Lead Health Economist:**	
**Professor Fiona Campbell**<br>Consultant Gastrointestinal Pathologist<br>Royal Liverpool University Hospital<br>Prescot Street<br>Liverpool<br>L7 8XP<br><br>Tel: +44 (0) 0151 7065887<br>Fax: 0151 7065859<br>Email:<br>Fiona.Campbell@rlbuht.nhs.uk	**Alan Haycox**<br>Management School<br>University of Liverpool<br>Chatham Building<br>Chatham Street<br>University of Liverpool,<br>L69 7ZH<br><br>Tel: +44 (0)151 795 3612<br>Fax Number:<br>E-mail:<br>A.R.Haycox@liverpool.ac.uk	

Contact Details: Individuals

Individual Authorised to Sign the Protocol and Protocol Amendments on behalf of the Sponsor:	Chief Investigator (CI):	Medical Expert who will Advise on Protocol Related Clinical Queries (If other than CI):
**Mr Christopher Halloran**<br>Department of Molecular and Clinical Cancer Medicine<br>Institute of Translational Medicine<br>University of Liverpool<br>L69 3GA.<br>Tel: +44 (0)151 706 4087<br>Fax: +44 (0)151 706 5826<br>E-mail:<br>halloran@liverpool.ac.uk<br><br>**Dr Seema Chauhan,**<br>Operational Director or<br>**Miss Charlotte Rawcliffe**,<br>Deputy Operational Director<br>Cancer Research UK<br>Liverpool Cancer Trials Unit<br>University of Liverpool<br>1st Floor, Block C,<br>Waterhouse Building<br>3 Brownlow Street<br>Liverpool<br>L69 3GL<br>Tel: +44 (0)151 794 8938<br>Fax: +44 (0)151 794 8930<br>Email: chauhans@liv.ac.uk	**Mr Christopher Halloran**<br>Department of Molecular and Clinical Cancer Medicine<br>Institute of Translational Medicine<br>University of Liverpool<br>L69 3GA.<br>Tel: +44 (0)151 706 4087<br>Fax: +44 (0)151 706 5826<br>E-mail:<br>halloran@liverpool.ac.uk	**Professor Paula Ghaneh**<br>Department of Molecular and Clinical Cancer Medicine<br>5th Floor UCD Building<br>Daulby Street<br>Liverpool<br>L69 3GA<br><br>Tel: +44 (0) 151 706 4170<br>Fax: + 44 (0) 151 706 5826<br>Email:<br>p.ghaneh@liverpool.ac.uk
**Medical Expert who will Evaluate SAE Reports (If other than CI):**	**Independent Reviewer of Operative Photographs**	**Trial Coordinator:**
**Professor Paula Ghaneh**<br>Department of Molecular and Clinical Cancer Medicine<br>5th Floor UCD Building<br>Daulby Street<br>Liverpool<br>L69 3GA<br><br>Tel: +44 (0) 151 706 4170<br>Fax: + 44 (0) 151 706 5826<br>Email:<br>p.ghaneh@liverpool.ac.uk	**Mr Dhanny Gomez**<br>Queens Medical Centre<br>Derby Road<br>Nottingham<br>NG7 2UH<br><br>Tel: 0115 924 9924<br>Fax:<br>Email:<br>Dhanny.Gomez@nuh.nhs.uk	**Kellie Platt**<br>Cancer Research UK:<br>Liverpool Cancer Trials Unit<br>1st Floor, Block C<br>Waterhouse Building<br>1-3 Brownlow Street<br>Liverpool<br>L69 3GL<br>Tel: +44 (0)151 795 5266<br>Fax: +44 (0)151 794 8930<br>Email:<br>kellie.platt@liverpool.ac.uk
**Trial Statistician:**	**Data Manager:**	

Contact Details: Individuals

**Fotis Polydoros**<br>Cancer Research UK:<br>Liverpool Cancer Trials Unit<br>1st Floor, Block C<br>Waterhouse Building<br>1-3 Brownlow Street<br>Liverpool<br>L69 3GL<br>Tel: +44 (0)151 794 8335<br>Fax: + 44 (0)151 794 8930<br>Email:<br>f.polydoros@liverpool.ac.uk	**Abbie Gerard**<br>Cancer Research UK:<br>Liverpool Cancer Trials Unit<br>1st Floor, Block C<br>Waterhouse Building<br>1-3 Brownlow Street<br>Liverpool<br>L69 3GL<br>Tel: +44 (0)151 795 5268<br>Fax: +44 (0)151 794 8930<br>Email:<br>agerard@liverpool.ac.uk	

Contact Details: Independent Oversight Committees

Independent and Safety Monitoring Committee (ISDMC)		
**Chair**<br>Dr Adrian Bloor<br>The Christie NHS Foundation Trust<br>Wilmslow Road<br>Manchester<br>M20 4BX<br><br>Tel: +44 (0) 161 446 3869<br>Fax : +44 (0) 161 446 3940<br>Email :<br>adrian.bloor@christie.nhs.uk	**Independent Clinician / Vice Chair**<br>Professor Daniel Hochhauser<br>University College London<br>235 Euston Road<br>London<br>NW1 2BU<br><br>Tel: +44 (0)203 447 9093<br>Email:<br>D.Hochhauser@ucl.ac.uk	**Statistician**<br>Andre Lopes<br>Cancer Research UK & UCL Cancer Trials Centre<br>Cancer Institute<br>University College London<br>90 Tottenham Court Road (5th floor)<br>London W1T 4TJ<br><br>Tel: +44 (0)20 7679 9557<br>Fax:+ 44 (0)20 7679 9899<br>E-mail:<br>andre.lopes@ucl.ac.uk
**Independent Members of the Trial Steering Committee (TSC)**		
**Chair**<br><br>Mr Richard Charnley<br>Surgical Services<br>The Newcastle upon Tyne Hospitals NHS Foundation Trust<br>Freeman Hospital<br>Freeman Road<br>High Heaton<br>Newcastle upon Tyne<br>NE7 7DN<br><br>Tel:<br>Email:<br>Richard.Charnley@nuth.nhs.uk	**Independent Clinician / Vice Chair**<br>Professor Terry Jones<br>Royal Liverpool University Hospital Prescot Street<br>Liverpool L7 8XP<br><br>Tel: 07702 816 180<br>Email:<br>T.M.Jones@liverpool.ac.uk	**Independent Statistician**<br>Dr Jane Warwick<br>Warwick Clinical Trials Unit<br>Division of Health Sciences<br>Warwick Medical School<br>The University of Warwick<br>Coventry<br>CV4 7AL<br><br>Tel: + 44 (0)2476 522498<br>E:<br>j.warwick@warwick.ac.uk
**Patient Representative**<br>John Richardson<br>C/o Trial Co-ordinator		

Table of Contents

1 Protocol Summary 12
2 Background Information 14
2.1 Introduction 14
2.2 Rationale 14
2.3 Objectives 17
2.4 Potential Risks and Benefits 17
3 Selection of Centres/Clinicians 19
3.1 Centre/Clinician Inclusion Criteria 19
3.2 Centre/Clinician Exclusion Criteria 20
4 Trial design 21
4.1 Overall Design 21
4.2 Pilot 21
4.3 Primary Endpoint 21
4.4 Secondary Endpoint(s) 21
5 Study Population 23
5.1 Inclusion Criteria 23
5.2 Exclusion Criteria 23
5.3 Transfer and Withdrawal 23
6 Enrolment and Randomisation 25
6.1 Screening 25
6.2 Enrolment/ Baseline 25
6.3 Randomisation 26
7 Trial Treatment/s 27
7.1 Introduction 27
7.2 Arm A: Blumgart method of pancreatico-jejunostomy 27
7.3 Arm B: Cattell-Warren method of pancreatico-jejunostomy 27
7.4 Unblinding 27
7.5 Concomitant Medications/Treatments 27
7.6 Octreotide 28
7.7 Surgical Drains 28
7.8 Pancreatic Duct Stent 28
7.9 Co-enrolment Guidelines 29
8 Assessments and Procedures 30
8.1 Schedule of Trial Procedures 30
8.2 Procedures for assessing Efficacy 34
8.3 Procedures for Assessing Safety 37
8.4 Other Assessments 38
8.5 Substudies 39
8.6 Loss to Follow-up 40
8.7 Trial Closure 40
9 Quality Assurance 41
9.1 Blinding 41
9.2 Pilot 41
9.3 Standardisation of Surgical Procedures 41
9.4 Operative Photographs 42
10 Statistical Considerations 43

10.1 Method of Randomisation ... 43
10.2 Outcome Measures ... 43
10.3 Sample Size ... 43
10.4 Interim Monitoring and Analyses ... 44
10.5 Analysis Plan ... 44

11 Safety ... 46
11.1 Terms and Definitions ... 46
11.2 Notes on Adverse Event Inclusions and Exclusions ... 46
11.3 Notes on Severity / Grading of Adverse Events (Surgical Complications) 47
11.4 Relationship to Trial Procedure ... 48
11.5 Expectedness ... 49
11.6 Follow-up After Adverse Events (Surgical Complications) ... 50
11.7 Reporting Procedures ... 50
11.8 Responsibilities – Investigator ... 52
11.9 Responsibilities – CR:UK LCTU ... 53

12 Ethical Considerations ... 54
12.1 Ethical Considerations ... 54
12.2 Ethical Approval ... 54
12.3 Informed Consent Process ... 54
12.4 Study Discontinuation ... 55

13 Regulatory Approval ... 56

14 Trial Monitoring ... 57
14.1 Source Documents ... 57
14.2 Data Capture Methods ... 58
14.3 Monitoring at LCTU ... 59
14.4 Clinical Site Monitoring ... 59
14.5 Records Retention ... 60

15 Indemnity ... 61

16 Financial Arrangements ... 62

17 Trial OVERSIGHT Committees ... 63
17.1 Trial Management Group (TMG) ... 63
17.2 Trial Steering Committee (TSC) ... 63
17.3 Independent Data and Safety Monitoring Committee (IDSMC) ... 63

18 Publication ... 64

19 Protocol Amendments ... 65
19.1 Version 1 (22.09.2014) ... 65
19.2 Version 2 (06.10.2014) ... 65
19.3 Version 3 (10.10.2014) ... 65
19.4 Version 4 (05.10.2015) ... 65
19.5 Version 5 (11/05/2015) ... 67
19.6 Version 6 (22/03/2016) ... 68

20 References ... 72

Appendices ... 75

Appendix A: Tables ... 76

Appendix B: EORTC Quality of Life Questionnaire (QLQ-C30) ... 78

Appendix C: EQ-5D-3L.. 80
Appendix D: Grading Tables 83

Glossary

AE	Adverse Event
AR	Adverse Reaction
CI	Chief Investigator
CRF	Case Report Form
CTU	Clinical Trials Unit
GP	General Practitioner
IDSMC	Independent Data and Safety and Monitoring Committee
IEC	Independent Ethics Committee
REC	Research Ethics Committee
PG	Pancreatico – Gastrostomy
PI	Principal Investigator
PJ	Pancreato-Jejunostomy / Pancreatico-Jejunostomy
POPF	Post-Operative Pancreatic Fistula
PPPD	Pylorus Preserving Pancreatoduodenectomy
R&D	Research & Development
SAE	Serious Adverse Event
SAR	Serious Adverse Reaction
SPC	Summary of product characteristics
SSI	Surgical Site Infections
SUSAR	Suspected Unexpected Serious Adverse Reaction
TSC	Trial Steering Committee
UAR	Unexpected Adverse Reaction

1 PROTOCOL SUMMARY

**Title:**	Cattell Warren versus Blumgart techniques of pancreatico-jejunostomy following pancreato-duodenectomy – a double blinded multi-centre trial **(Acronym; PANasta Trial)**
**Phase:**	III
**Sample Size:**	506 (253 in each arm), powered to detect a 10% absolute difference in primary end point
**Main Inclusion Criteria:**	- Patients undergoing an elective pancreato-duodenectomy for presumed malignancy.<br>- Ability of the subject to understand the nature and consequences of the trial.<br>- Ability to provide written informed consent.<br>- Age 18 or greater
**Main Exclusion Criteria:**	- Patients undergoing extended pancreato-duodenectomy<br>- Left, central or total pancreatectomy<br>- Arterial resection or multi-visceral resection<br>- Previous pancreatic surgery<br>- Surgery for known chronic pancreatitis.<br>- Recruited to any other pancreatic resection trial (unless prior approval from CI).<br>- Pregnant women<br>- Women of childbearing potential, including women whose last menstrual period was less than one year prior to screening, unable or unwilling to use adequate contraception from time of consent up to the day of surgery.
**Number of Sites:**	7+ UK centres
**Study Duration:**	60 months
**Description of Agent/ Intervention:**	Technique of standardised anastomosis; Cattell Warren versus Blumgart methods.
**Objectives:**	
Primary:	Leak/fistula rate at the pancreatico-jejunostomy during in-patient hospital stay. Assessment by ‘Bassi criteria’. Measured by presence/content of drain exudate and amylase content analysis within fluid.
Secondary:	Entry into adjuvant therapy/clinical trials of adjuvant therapy; Mortality Rate; Overall Survival; Delayed Gastric Empting; Rate of wound infections; Rate of Pulmonary Infection; Rate of post-operative fluid collections; Operation time; Rate of intra and post-operative bleeding; Rate of re-operation; Rate of venous thrombo-embolism; Hospital stay; Quality of life and health economic assessments.

Protocol Summary - continued

Schematic of Study Design:

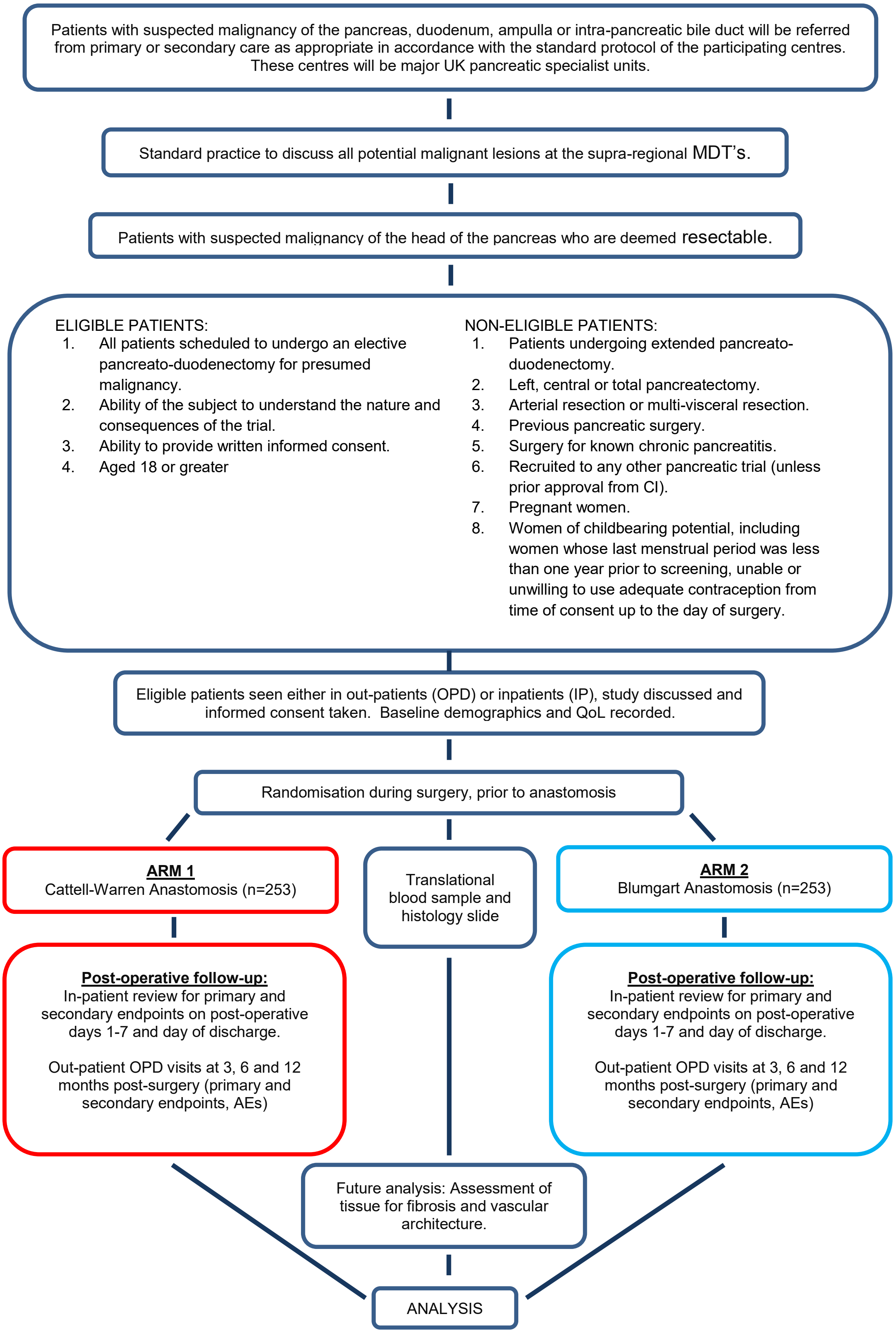

2 BACKGROUND INFORMATION

2.1 Introduction

Pancreato-duodenectomy as a procedure is nearly 100 years old. Between 1912 and 1922 the first ‘series of en-block pancreatic resections’ were reported by Herchel, Kausch and Tenai [1]. These were performed for treatment of periampullary tumours and as a two-stage procedure, with a purse string anastomosis between pancreatic remnant and the jejunum [2]. In 1935, a further modification was reported by Whipple, Parsons and Mullins [3] who successfully employed a two-stage procedure with closure of the duodenal and pancreatic stumps, this avoiding further anastomosis. The ‘Whipple’ procedure was refined and standardised during the 1940’s to treat head of pancreas cancer [4, 5] and evolved into a single operation including a pancreatojejunal anastomosis and gastric resection. As early as 1944 [6] preservation of the pylorus was suggested but this was viewed with scepticism until 1978 [7] when the pylorus preserving pancreatoduodenectomy (PPPD) became accepted. Large series have concluded that there is no compromise to long term survival between classical Kausch-Whipple or pylorus preserving pancreatoduodenectomy [8, 9], and confirmed in recent systematic reviews [10, 11] providing patients are appropriately selected. Hence the current ‘standard’ resection preserves the pylorus with en-block resection of the pancreatic head to the right of the portal vein, the extra-hepatic bile duct, the gall bladder, the duodenum and the proximal jejunum. This operation is used in the treatment of an increasing number of conditions in the head of the pancreas; namely adenocarcinoma and chronic pancreatitis. Due to centralisation of pancreatic service in high volume specialist centres, mortality is often lower than 5%. Despite this, morbidity still remains high often around 50% for all causes [12, 13]. In spite of technological advances a major cause for this morbidity and lengthy hospital stay is failure of the pancreatic anastomosis to heal. The literature quotes a pancreatic leak/fistula rate of between 2% and in excess of 20%, (reviewed in [14]). The wide range is due partly to a lack of a consensus definition [13], however recently this has been proposed [14] and is beginning to gain support and be quoted in literature. Health economic studies of pancreatic resection from the USA have reported an average length of stay of just over 20 days with an approximate cost of $1000 per day [15-18]. Edge et al [15], showed in a series of pancreatic resections at 26 American University hospitals a strong association between complications, length of stay of 13 days and cost $30,000. Stay and cost increases proportionally with complications, such that a minor complication increased stay to 18 days ($43,000) and a major complication to 32 days ($90,000). Data up to 2005 suggests length of hospital stay is similar but that cost has increased by 63% [19], if similar extrapolations are made, a major complication will cost ~ $146,000 in today’s money.

2.2 Rationale

Scientific Rationale and Systematic Review

The question of definition of post-operative pancreatic fistula (POPF) has been addressed; however, this question is sullied by the multitude of techniques of pancreatic remnant reconstruction. The sheer number of options available is testament to personal preference, difficulty of technique and poor evidence base. Broadly, techniques separate into pancreatic stump closure, anastomosis of

pancreas to stomach (PG) or anastomosis of pancreas to jejunum (PJ). Stump closure is a poor option as there are excessive rates of pancreatic fistula, pancreatitis and post-operative exocrine failure [20]. This technique has largely been abandoned. Anastomosis of pancreatic remnant to stomach (Pancreatico – Gastrostomy – PG) is a relatively uncommon reconstruction in the UK. Although it purports to ease of construction, endoscopic evaluation and separate pancreatic juice and bile drainage as advantageous; this anastomosis is prone to post-operative haemorrhage however. Advocates quote the superior nature of this technique over that of a jejunal loop from a poor literature base. Those studies which are comparable are from different cohorts, are retrospective [21, 22] and are compared to pancreatic-jejunal anastomosis with un-acceptable rates of fistula. Furthermore a recent meta-analysis [23] has confirmed that there is no confirmed superiority between either technique of PG or PJ.

Anastomosis between the pancreatic stump and the jejunum is regarded as the ‘standard’ technique by most UK pancreatic surgeons. There are 2 main ways to perform this (with multiple variations): Invagination of the pancreatic stump into the jejunum (pancrea*to*-jejunostomy) or anastomosis of the pancreatic duct to the jejunal mucosa (pancrea*tico*-jejunostomy), with covering parachymal sutures. To date there are 4 randomised controlled trials specifically looking at PJ (summarised in table 1, appendix A). Unfortunately none of these are comparable in a meta-analysis as there are serious differences in each study [24-27]. Bassi *et al* [24], Langrehr *et al*. [26] and Berger *et al*. [25] compare pancreato-jejunostomy to pancreatico-jejunostomy – the latter a 2 centre US study. Bassi and Langrehr showed no difference between the techniques. Beger *et al.* showed a higher pancreatic leak rate for pancreatico-jejunostomy. However this study has potential bias toward a softer (and more likely to leak) gland in that group; in addition the odds ratio of a leak between the two centres was 2.2 (p=0.04), suggesting one centre was unfamiliar with the type of reconstruction used. Peng *et al.* [27] compared two different methods of pancreato-jejunostomy. In addition there are major technical differences between the technique of pancreato-jejunostomy between these studies: Bassi; single layer interrupted, Langrehr; mattress, Peng: binding and Berger; double layer interrupted including the duct. There are also important differences between patients who underwent pancreatico-jejunostomy: Bassi; all had octreotide and some an external pancreatic stent, Langrehr; variable use of octreotide and no stent, Beger; no octreotide and an implied use of internal pancreatic duct stenting.

In addition there are a further 6 non-randomised trials – see table 2, appendix A. Batignani *et al.* [28] and You *et al.* [29]; compared pancreato-jejunostomy to pancreatico-jejunostomy and found no difference between groups. Satoi *et al.* [30] compared pancreato-jejunostomy to pancreatico-jejunostomy and found significantly fewer pancreatic fistula in the pancreatico-jejunostomy group, Bassi grade B/C (p=0.04); however pancreatic fistula rate remained similar 14% vs.27%, p=0.08. Lee *et al.* [31] compared an interrupted to a continuous method of pancreatico-jejunostomy. This study used discontinuous time frames and made claim to fewer major pancreatic fistula with the continuous technique, although in the light of recent classification of pancreatic fistula [14], this no longer holds true. Fragulidis *et al.* [32] compared pancreato-jejunostomy to pancreatico-jejunostomy on long and short roux limbs. Claim was made that a long limb and a duct-to-mucosa anastomosis is preferable – although there is no difference between both anastomosis on a short limb suggesting the effect is due to a long roux limb only.

Leslie Blumgart (Memorial-Sloan Kettering, NY) has devised a most intuitive anastomosis as it involves both duct-to-mucosa stitches and a full thickness pancreatic “U” stitch, in effect a mattress stitch (see FIGURE 1). Since its description and publication of initial results there have been several reports of its advantages. Grobmyer *et al.* [33] retrospectively analysed 187 consecutive patients undergoing panreatico-duodenectomy and pancreatic-enteric reconstruction with pancreatico-jejunostomy using the “Blumgart pancreaticojejunostomy” between 2000 and 2007. Pancreatic anastomotic failure occurred in 38 patients (20.3%) overall. Most events were grade A and did not alter clinical management. Clinically significant pancreatic anastomotic failure (grade B or C) occurred in only 6.9% of patients. Soft texture of the pancreas was significantly associated with pancreatic anastomotic failure (p=0.005). Of the 38 anastomotic failures, most occurred in association with soft glands (n = 28, 74%). Patients with pancreatic anastomotic failure (median duct size, 3mm; range 2 to 14mm) had significantly smaller pancreatic duct size compared with those who did not have anastomotic failure (median duct size, 4mm; range 1 to 13mm, p=0.008). Mishra *et al.* [34] undertook “BA” pancreatico-jejunostomy in 98 patients who had undergone a whipples pancreato-duodenectomy. Fifteen patients (15.3%) developed pancreatic leak. Eight of these were grade A, 4 were grade B and 3 were grade C as per ISGPF criteria. Five of these anastomotic leaks were in patients with firm pancreas (5/55) and 10 were in soft pancreas (10/43; p=0.064). Leaks were seen in 10/48 of pancreas with duct 3mm or less and in 5/50 of ducts more than 3mm (p=0.135). Kleespies *et al*. [35] compared a standard Cattell-Warren pancreatico-jejunostomy with a Blumgart technique (BA); this reduced pancreatic leak rate from 13% to 4% (p=0.03) and overall complications from 31% to 15% (p=0.015) in favour of BA. This study included 182 patients and was *not* randomised.

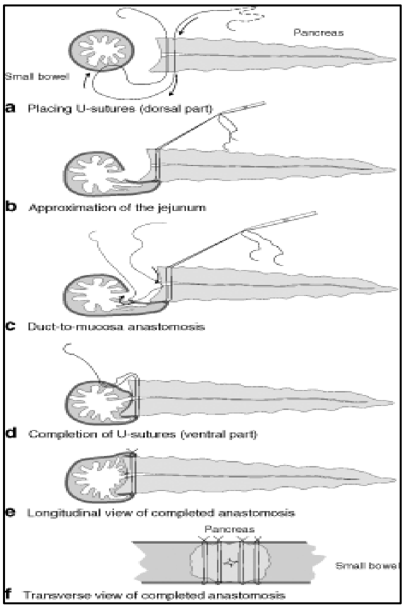

FIGURE 1
Blumgart anastomosis (BA): Demonstrating the full thickness pancreatic “U” stitch.

Kleespies et al.[35]

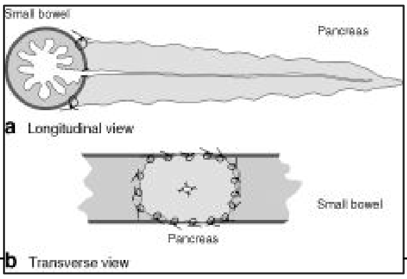

FIGURE 2
Cattell-Warren Anastomosis (CWA)

Kleespies et al. [35]

2.3 Objectives

<u>*Does a Blumgart anastomosis reduce pancreatic remnant leak?*</u> An improved method of pancreatic reconstruction should reduce pancreatic fistula, decrease all complications, hospital stay, cost and promote enhanced recovery programs. Moreover, fewer post-operative pancreatic fistula should enable more patients to take advantage of adjuvant therapy (main stream or within trials), as they will regain the required level of fitness faster. Ultimately this approach should increase survival from operated pancreatic cancer. In addition this study will attempt to identify reasons why pancreatic anastomoses fail by collecting histological slides and fresh material (see schema) for future analysis assessments. Hence this randomised, double blinded, multi-centred study is to directly compare 2 methods of pancreatico-jejunostomy construction; standard treatment = Cattell-Warren Anastomosis (CWA), trial treatment = “Blumgart Anastomosis” (BA). The proposed study (PANasta) would be the only RCT to date, which investigates the difference between two methods of duct-to-mucosa pancreatic anastomosis (PANCREATICO-JEJUNOSTOMY); CWA vs. BA.

2.4 Potential Risks and Benefits

2.4.1 Potential Risks

Both techniques are regularly used and are reported in the literature. There are no additional potential risks of subjecting patients to different types of anastomosis of the pancreatic remnant providing the operating surgeon is familiar with the proposed techniques. In order to assure this, sites and local Principle Investigators have been selected because they have familiarity with both techniques and the individual surgeons would be happy to randomise subjects to either technique. The standardisation of the operative techniques will be ensured by:

- Consensus meetings -.A pre-pilot study consensus will be arrived at regarding the essentials of each anastomosis and the likely key steps, the post-operative management of drains, pancreatic duct stents, the use of octreotide and the timing of operative photographs. This information will be developed into a pilot phase operative manual.
- Investigator Meeting - Prior to the start of the trial, workshops will be held for all participating sites to provide training. The pilot phase operative manual will be finalised and issued to the two sites taking part in the feasibility / pilot phase of the trial.
- Operative Manual – following the feasibility / pilot phase the operative manual will be reviewed and where applicable adapted in accordance with the information gained from the feasibility / pilot phase. A finalised operative manual for each anastomosis will be formulated and contain steps that are A) Mandatory to the construction of a safe anastomosis; B) Prohibited for the construction of a safe anastomosis and C) Equivocal steps where options between methods or techniques will be permitted. These manuals will be distributed to all participating sites and will form the quality assurance for the techniques beyond the initial feasibility/pilot phase. The attendance of surgeons at the meetings and their willingness to engage in the study and agree the mandatory and prohibited steps of the operation to be performed in the main trial will be important outcomes

of the feasibility/pilot phase.

- Operative photographs - Digital operative photographs will be taken at the key steps during anastomosis construction. These will be reviewed by the Chief Investigator and a second reviewer to confirm quality and consistency across sites.

2.4.2 Known Potential Benefits

An improved anastomosis should reduce the rate of troublesome pancreatic fistulae, decrease hospital stay, speed up recovery and drive the cost of hospital care down. Moreover, fewer post-surgery fistulae will increase the number of patients who can undertake adjuvant therapies, which is crucial to long-term survival. The *PANasta* study should not only demonstrate this but is also an opportunity for the pancreatic surgical community in the UK to work together in a unified fashion.

3 SELECTION OF CENTRES/CLINICIANS

Each participating centre (and investigator) has been identified on the basis of:

- Having at least one lead clinician with a specific interest in, and responsibility for supervising, treating and managing patients with advanced pancreatic cancer.
- Showing enthusiasm to participate in the study.
- Local expertise on pancreatico-jejenostomy methods of reconstructing the pancreatic remnant.
- Ensuring that sufficient time, staff and adequate facilities are available for the trial
- Providing information to all supporting staff members involved with the trial or with other elements of the patient's management.
- Acknowledging and agreeing to conform to the administrative and ethical requirements and responsibilities of the study, including signing up to the Good Clinical Practice (GCP) and other regulatory documentation.
- Suitable MDT meeting structure to identify patients and ensure the pathologists and surgeons are involved in patient screening to ensure the required samples are made available for the associated biomarker studies.

3.1 Centre/Clinician Inclusion Criteria

a. Positive Site Specific Assessment (SSA) by Research and Development (R&D) Department
b. Completed/signed Research Site Agreement
c. Receipt of evidence of completion of (a) & (b) by LCTU
d. Completion and return of 'Signature and Delegation Log' to LCTU
e. Curriculum Vitae (CV) including a record of International Conference for Harmonisation (ICH) of GCP training-Principal Investigator (PI)
f. CV including a record of ICH GCP training –Other personal on the delegation log
g. Clinical Study Protocol Receipt Form
h. Local Laboratory accreditation/Quality check
i. Local Laboratory reference ranges
j. Completion of test SAE reported via web
k. Completion of study questionnaire
l. Patient information sheet, consent form and a GP letter on trust paper

Tertiary pancreatic surgery referral units, hospital inpatients and outpatients will be included in order to ensure standard practice and similar case mix. The study will be co-ordinated from the Cancer Research UK Liverpool Cancer Trials Unit to ensure high standards of regulatory and quality control. The proposed co-ordinating unit (LCTU) is renowned, and treats over 400 patients with suspected pancreatic cancer each year. Applicants are all experts in their fields. The local cancer trials unit has an excellent track record in large-scale clinical trials in pancreatic cancer. Local statistical and health economic are integral. All regional centres were approached and invited to participate in this trial. Final selection was determined on willingness of centres to participate and local expertise on pancreatico-jejenostomy methods of reconstructing the pancreatic remnant.

3.2 Centre/Clinician Exclusion Criteria

Those centres that did not express a desire to take part were excluded. Centres where pancreatic remnant reconstruction was not routinely performed by the method of pancreatico-jejunostomy were also excluded. Centres/clinicians who did not feel they could randomise without clinical equipoise were also excluded.

4 TRIAL DESIGN

4.1 Overall Design

Cattell Warren versus Blumgart techniques of pancreatico-jejunostomy following pancreato-duodenectomy – a double blinded, multi-centred UK trial. (Acronym; *PAN*asta Trial). The total sample size of 506 patients (253 in each arm) has been calculated to detect a 10% difference in primary end point. This assumes an attrition rate of 5%. Non-compliance would occur in the event of unresectable disease, assumed at 10%. The primary end point is rate of post-operative pancreatic fistulae.

4.2 Pilot

Following the constructive feedback from CTAAC and after discussion with a surgeon with expertise in trial methodology (Jane Blazeby, a co-applicant); a decision to incorporate a feasibility/ pilot study within the first 6 months of this project has been adopted. This will run in 2 centres initially from the day of the trial opening and will evaluate in step wise fashion: 1) The number of patients eligible at each site; 2) the number of patients enrolled; 3) the number of patients randomised and finally 4) the number of patients who took up the randomisation to ensure that the local investigator is performing the surgery as per the randomisation result. This will inform us of the real figures for stopping this trial for futility in recruitment. In addition an interim analysis will be undertaken once 208 patients have been randomised. Hence, the first six months of the study will be run as a feasibility/pilot study with a decision, whether to continue or not dependent upon actual numbers undertaking enrolment as opposed to numbers of patients who would be eligible. After completion of the initial feasibility/pilot phase of the study, the researchers will meet and formalise the operative manual, which will include those steps, which are mandatory, those steps that are prohibited and the team will identify steps that are optional for each arm of the trial. This will ensure the standardisation of the technique following on from the pilot

The pilot phase concluded on 23rd October 2015. The TSC and DMC deemed the pilot phase a success following review of recruitment data and the trial continues.

4.3 Primary Endpoint

The presence or absence of a post-operative pancreatic fistula (POPF) as defined by Bassi *et al*. [14].

4.4 Secondary Endpoint(s)

- Entry into programs of adjuvant therapy/clinical trials of adjuvant therapy
- Mortality rate
- Overall Survival
- Rate of delayed gastric emptying
- Rate of wound infections

- Rate of pulmonary infection
- Rate of post-operative fluid collections
- Rate of intra and post-operative bleeding
- Operation time
- Rate of re-operation
- Rate of venous thrombo-embolism
- Length of hospital stay
- Quality of life
- Health economic evaluation

5 STUDY POPULATION

5.1 Inclusion Criteria

Subjects meeting all of the following criteria will be considered for this trial:

- Patients undergoing an elective pancreato-duodenectomy for presumed malignancy.
- Ability of the subject to understand the nature and consequences of the trial.
- Ability to provide written informed consent.
- Age 18 or greater

5.2 Exclusion Criteria

Subjects with the following criteria will not be entered into this trial:

- Patients undergoing extended pancreato-duodenectomy
- Left, central or total pancreatectomy
- Arterial resection or multi-visceral resection
- Previous pancreatic resection
- Surgery for known chronic pancreatitis
- Recruited to any other pancreatic resection trial.
- Pregnant women
- Women of childbearing potential, including women whose last menstrual period was less than one year prior to screening, unable or unwilling to use adequate contraception from time of consent up to the day of surgery.

5.3 Transfer and Withdrawal

In consenting to the trial, patients are consented to trial treatment, follow-up and data collection. If voluntary withdrawal occurs, the patient should be asked to allow continuation of scheduled evaluations, complete an end-of-study evaluation, and be given appropriate care under medical supervision until the symptoms of any surgical complications resolve or the subject's condition becomes stable.

5.3.1 Patient Transfers

For patients moving from the area, every effort should be made for the patient to be followed-up at another participating trial centre and for this trial centre to take over responsibility for the patient or for follow-up via GP.
A copy of the patient CRFs should be provided to the new site. The patient will have to sign a new consent form at the new site, and until this occurs, the patient remains the responsibility of the original centre. The LCTU should be notified in writing of patient transfers.

5.3.2 Withdrawal from Trial Intervention

Subjects are free to leave the trial at any point without the need to give reasons for their decisions. If voluntary withdrawal occurs prior to the surgical intervention the patient will not be randomised and no further trial data will be

collected on that patient. In addition the subject may be withdrawn from the trial for the following reasons:

- At their request or at the request of a legal representative
- If, in the investigators opinion, continued participation within the trial would be detrimental to the wellbeing of the subject.

In all cases the reason for the subject's withdrawal prior to randomisation *must* be documented on the PANasta screening log (accessed via the LCTU Portal) and in the medical records.

5.3.3 Withdrawal from Post-Intervention Follow Up

Patients may wish to withdraw from the trial following the surgical intervention. In such cases no further data should be collected but data up to this time can be included in the trial if anonymised. If the patient explicitly states their wish not to contribute further data to the study, an End of Study form should be completed recording the reason for withdrawal.

Patients may also wish to remove all data and/or any samples collected up to the point of withdrawal from the trial analysis. In such cases this must be clearly indicated on the End of Study form along with the reason for withdrawal. It must be noted that any safety data collected up to the point of withdrawal cannot be removed from the trial analysis.

6 ENROLMENT AND RANDOMISATION

6.1 Screening

Screening and identification of eligible patients will take place at the research site's pancreatic multidisciplinary team (MDT) meeting. All patients with suspected pancreatic malignancy will undergo standard evaluation: Contrast enhanced multi-detector CT scan +/- endoluminal ultrasound (EUS) which will be discussed at the MDT. `

Patients recommended for resection on the basis of a high likelihood of a malignant lesion will be contacted and provided with a participant information sheet. The best time to undertake this will be dependent upon each individual unit's preference. Informed consent will be obtained after the patient has had sufficient time (at least 24 hours) to make a decision and prior to any baseline assessments and study enrolment.

It is not necessary for patients to have a histological diagnosis of malignancy before surgery.

All potentially eligible patients identified must be documented on the LCTU web portal "Screening Log"; including individuals who decide not to participate in or are unsuitable for the study. Screening details should be entered into the portal and this will automatically generate a screening number and a confirmation email containing these details will be sent to site staff. The screening log can be printed from the portal at any time to allow for storage in the Investigator Site File.

6.2 Enrolment/ Baseline

All patients who are listed to undergo pancreato-duodenectomy for presumed/actual malignancy, who agree to participate will be consented and enrolled. This ideally will take place on the same visit that subjects are assessed anaesthetically and deemed suitable for major surgery. This strategy is the most feasible as the majority of tertiary centres will take patients beyond their immediate geographical area. Certain units may have well evolved local policies, which would be equally acceptable.

The following baseline assessments must be completed at enrolment after the patient has provided written informed consent and within four weeks of surgery. Some investigations are performed as part of routine pre-surgery assessment and these may also be used as part of baseline assessments provided they are performed within the appropriate time frame:

1. Demographics (height, weight, etc)
2. Diabetic status
3. Smoking and alcohol status
4. Pancreatic Endocrine Insufficiency Status
5. Suspected date of diagnosis
6. Medical History (including symptoms and relevant tests)
7. Family Medical History
8. Pregnancy test (where appropriate)
9. Quality of Life Questionnaires (QLQ-C30 and EQ5D)

Patients will be enrolled onto the study by the LCTU. Enrolment can only occur following the completion and forwarding of the trial enrolment documents by the investigators:

1. Eligibility checklist
2. Enrolment forms
3. Copy of signed Patient Consent Form

Enrolment – Tel: 0151 795 5268 Fax: 0151 794 8930

If fax is unavailable, secure data transfer via Datanywhere is available. Arrangements to be made with LCTU to use this application.

(Note that the LCTU is open from 0900 – 1700, Monday – Friday, excluding public holidays)

When a patient has been enrolled, a confirmation email will be sent to the site detailing the patients MACRO ID, site, patient initials, DOB, screening number, proposed date of surgery, details of consent and a link to the patient in Treatment Allocation Randomisation System (TARDIS).

6.3 Randomisation

Randomisation will be undertaken on the day of surgery by the surgeon. This will be undertaken intra-operatively, following pancreatic head excision, just prior to pancreatic remnant reconstruction. THIS HAS BEEN EXPLICITLY REQUESTED BY THE FUNDER CR-UK, as it is felt that this will maximise the number of enrolled subjects who will take up the allocated randomisation.

Only after a patient has been enrolled into the trial and a confirmation email has been received can patients be randomised. Patients are randomised via TARDIS, which is a web based randomisation tool.

To randomise the surgeon should follow the link in the enrolment confirmation email. The surgeon will be prompted to confirm eligibility of the patient along with the stratification factors, which will enable randomisation to one of the two treatment arms.

Patients will be randomised to one of the treatment arms in a ratio 1:1, with the following stratification factors:

1. Pancreatic texture: soft vs normal/hard
2. Pancreatic duct diameter: normal(≤3mm) vs dilated (>3mm)
3. Research site

Randomisation 24 hours a day via web

Web site: www.lctu.org.uk/tardis

7 TRIAL TREATMENT/S

7.1 Introduction

Patients will be randomised 1:1 to receive one of the two treatment arms, which are different methods of constructing a pancreatico-jejunostomy in the reconstruction of the pancreatic remnant following pancreato-duodenectomy.

7.2 Arm A: Blumgart method of pancreatico-jejunostomy

Re-construction of the pancreatic remnant following pancreatico-duodenectomy (either Kausch-Whipple or pylorus preserving) using a “Blumgart” method of pancreatico-jejunostomy. ***(INTERVENTION)***

7.3 Arm B: Cattell-Warren method of pancreatico-jejunostomy

Re-construction of the pancreatic remnant following pancreato-duodenectomy (either Kausch-Whipple or pylorus preserving) using a “Cattell-Warren” method of pancreatico-jejunostomy. ***(CONTROL)***

7.4 Unblinding

It is unlikely that this trial will require unblinding as both types of anastomosis are in routine usage in the centres that are participating. There are no additional potential risks of subjecting patients to different types of anastomosis and the care and management of patients is the same regardless of the type of anastomosis performed. In case of anastomotic leakage the majority of such leaks are managed conservatively. The operation notes will not include which Anastomosis has been performed but rather – “Anastomosis as per PANasta Trial randomisation”.

In the rare instance of reoperation, where it is desirable to know which anastomosis has been undertaken, the local research team will be able to unblind the surgical technique performed via a web based tool called TARDIS. Users will be required to log into TARDIS and confirm that the patient requires reoperation before the system will unblind.

7.5 Concomitant Medications/Treatments

This is a surgical trial comparing two different methods of reconstructing the pancreatic remnant following pancreatic head resection. All usual medications and standard of medical care is accepted.

7.5.1 Medications Permitted

All medications and treatments that are the usual practice of the unit in question are acceptable.

7.5.2 Medications Not Permitted/ Precautions Required

This is a surgical trial assessing two different methods of surgical reconstruction of the pancreas. There are no medications that are not permitted and no specific precautions required.

7.5.3 Data on Concomitant Medication

There is no requirement to record any medications that are being taken by the patient.

7.6 Octreotide

An initial dose 100ug of octreotide should be administered to all patients subcutaneously on the evening before surgery. This should be administered in accordance with local practice; therefore it is permissible to omit this dose if the patient is admitted on the morning of surgery.

Octreotide should then be administered at 100ug three times a day subcutaneously on the day of surgery (day 0) and post-operative days 1 to 6.

If a patient is discharged prior to post-operative day 6 then it is permissible for octreotide to be discontinued in accordance with discharge in accordance with local practice.

It may be necessary for Octreotide to continue past post-operative day 6 in accordance with local practice. It is not necessary to record any doses of Octreotide that are administered past post-operative day 6 on the CRF.

7.7 Surgical Drains

Surgical drains (of any description) must be employed and should be left in place for a minimum of 3 days after surgery. If on post-operative day 3, the drain amylase is normal, the drain may be removed (under the direction of the operating surgeon). may be removed before post-operative day 7 at the discretion of the Principle Investigator.

7.8 Pancreatic Duct Stent

The use of a pancreatic duct stent is mandatory in all patients.

7.9 Co-enrolment Guidelines

As a rule patients should not be co-enrolled into other pancreatic surgery trials. Exceptions to this do apply and in some cases patients in the PANasta trial may be recruited into other pancreatic surgery trials but this is at the discretion of the Chief Investigator. Prior approval must be obtained before patient enrolment.

Patients recruited to any trial that measures the same primary end point should not be recruited into the PANasta trial.

Entry into trials of other surgical procedures or medicinal products will not be restricted.

Any queries should be addressed to the trial co-ordinator.

8 ASSESSMENTS AND PROCEDURES

8.1 Schedule of Trial Procedures

- **Pre-surgery**

**Visit**	**Screening**	**Enrolment**
	To assess eligibility only (no data to be collected until consent)	Within 4 weeks of surgery
**Assessments / procedures**		
Written Informed Consent[1]		X
Assessment of eligibility criteria	X	X
Suspected date of diagnosis		X
Demographics (height, weight, etc)		X
Smoking and alcohol status		X
Review of Medical History (including symptoms and relevant tests)		X
Family Medical History		X
Pregnancy Test		X
Pancreatic Endocrine Insufficiency status		X
Diabetic status		X
QoL		X

1. Patient consent does not need to be within 4 weeks of surgery.

- **Day of surgery**

**Visit**	**Randomisation**
	Day of surgery (Day 0)
**Assessments / procedures**	
Octreotide review[1]	X
CA19-9	X
Full blood count[2]	X
Serum Biochemistry[3]	X
Clotting screen[4]	X
Blood Sample for translational study[5]	X
Histological sample for translational study[6]	X
Randomisation	X
Surgical Intervention	X
Take Operative photographs	X
Upload Operative Photographs[7]	X
Details of surgery	X
Operation time	X
Intra operative bleeding assessment	X

1. Initial dose of Octreotide (100ug) to be administered on the evening before surgery (if applicable) then 100ug 3 times daily on the day of surgery.
2. FBC (haemoglobin, Hba1c, platelets, absolute neutrophil count, white blood cell count, eosinophils, basophils, lymphocytes, monocytes) to be done pre-operatively either the day before or morning of surgery.
3. Serum biochemistry (sodium, potassium, calcium, urea, creatinine, eGFR, random glucose, albumin, bilirubin, alk.phosphatase, total protein, AST or ALT, GGT and CRP) to be done pre-operatively either the day before or morning of surgery.
4. Clotting screen (PT and APTT) to be done pre-operatively either on the day before or morning of surgery.
5. Translational blood samples (10ml EDTA tube and 8.5ml SST tube) to be taken pre-operatively either on the day before or morning of surgery.
6. Diagnostic H&E slide of the pancreatic neck transection margin.
7. Operative photographs should be uploaded on to the LCTU portal immediately after surgery.

- **Post-operative In-patient Review**

Visit	Post-operative day 1	Post-operative day 2	Post-operative day 3	Post-operative day 4	Post-operative day 5	Post-operative day 6	Post-operative day 7	Discharge
	1 day following surgery	2 days following surgery	3 days following surgery	4 days following surgery	5 days following surgery	6 days following surgery	7 days following surgery	Day of discharge
**Assessments / procedures**								
Octreotide review[1]	X	X	X	X	X	X		
Survival status[2]	X	X	X	X	X	X	X	X
Review of other complications (not endpoints)	X	X	X	X	X	X	X	X
Post-operative bleeding assessment	X	X	X	X	X	X	X	X
Re-operation review	X	X	X	X	X	X	X	X
Post- operative fluid collection (internal)			X	X	X	X	X	X
Fluid collection review (drain amylase to assess POPF)			X	X	X	X	X	X
Delayed gastric emptying assessment			X	X	X	X	X	X
Full blood count[3]					X (±2 days)			X (-2 days)
Serum Biochemistry[4]					X (±2 days)			X (-2 days)
Clotting screen[5]					X (±2 days)			X (-2 days)
Blood Sample for translational study[6]					X (+2 days)			
Pancreatic Endocrine Insufficiency status			X		X		X	X
Surgical site infections assessment								X
Venous thrombo-embolism assessment								X
Pulmonary infection assessment								X
Length of initial hospital stay								X
Surgical drain review								X
Diabetic status								X
Weight								X
QoL[7]								X

1. 100ug to be administered 3 times daily on post-operative days 1 to 6.
2. Death due to any cause from time of randomisation up trial closure must be reported by completing an End of Study Form.
3. FBC (haemoglobin, Hba1c, platelets, absolute neutrophil count, white blood cell count, eosinophils, basophils, lymphocytes, monocytes) to be done post-operatively on day 5 and day of discharge. A window of **±2** days applies to day 5 FBC only and a -2 day window applies at day of discharge.
4. Serum biochemistry (sodium, potassium, calcium, urea, creatinine, eGFR, random glucose, albumin, bilirubin, alk.phosphatase, total protein, AST or ALT, GGT and CRP) to be done post-operatively on day 5 and day of discharge. A window of **±2** days applies to day 5 serum chemistry only and a **-2** day window applied at day of discharge,
5. Clotting screen (PT and APTT) to be done post-operatively on day 5 and day of discharge. A window of **±2** days applies to day 5 clotting screen only and a **-2** day window applies at day of discharge.
6. Translational blood samples (10ml EDTA tube and 8.5ml SST tube) to be taken on post-operative day 5, a **+2** day window applies.
7. EORTC QLQ-C30 and EQ-5D and EQ-VAS to be completed.

- Follow up and End of Study

Visit	Visit 4 (3m FU)	Visit 5 (6m FU)	Visit 6 (12m FU)	End of Study / Overall survival
	3 months post surgery (±4 weeks)	6 months post surgery (±4 weeks)	12 months post surgery (±4 weeks)	At any point patient ends trial
Weight	X	X	X	
Pancreatic Endocrine Insufficiency status	X	X	X	
Diabetic status	X	X	X	
Surgical drain review	X			
Post- operative fluid collection (internal)	X			
Survival status[1]	X	X	X	X
Re-operation review	X	X	X	
Pulmonary infection assessment	X			
Surgical site infections assessment	X			
Venous thrombo-embolism assessment	X			
Fluid collection review (drain amylase to assess POPF)	X			
Re-admission review	X	X	X	
Adjuvant therapy review	X	X	X	
Review of other complications	X	X	X	
QoL[2]	X	X	X	
Reason for end of study				X

1. Death due to any cause from time of randomisation up to trial closure must be reported by completing an End of Study Form.
2. EORTC QLQ-C30 and EQ-5D and EQ-VAS to be completed.

8.1.1 Screening for study eligibility

Patients will be identified from clinic assessment and assessed for their eligibility for entry into the study. No data collection is required at this point. Prior to inclusion, patients should be provided with information regarding the trial and surgical difference of each technique.

8.1.2 Enrolment assessments

After a patient has been assessed for their eligibility and has provided written informed consent, the enrolment assessments set out in the table above should be performed within four weeks of surgery.

Patients who fulfil entry criteria will be enrolled by the LCTU (see section 6.2).

8.1.3 Before Surgery / Randomisation

Please refer to the table above for assessments performed at randomisation. Randomisation will be undertaken on the day of surgery by the surgeon. This will be undertaken intra-operatively, following pancreatic head excision, just prior to pancreatic remnant reconstruction.

On the day before or morning of surgery, pre-operative blood samples should be taken, which consist of the routine pre-operative evaluation in accordance with the participating centre and the translational blood samples. In addition, a histological sample will be taken intra-operatively (for diagnostic H&E slide). Please refer to section 8.5 for translational sub-study details.

If the timing of the routine pre-operative blood sample does not fit in line with sites routine practice and it is not practical to take bloods the day before or morning of surgery, <u>it is permissible for the routine pre-operative blood sample to be done in accordance with local practice.</u>

<u>The translational blood sample MUST be taken on the day before or morning of surgery.</u>

During the surgery three operative photographs will be taken of the anastomosis as described in section 8.4.2.

8.1.4 Post-operative assessments

Please refer to the table above for post- operative assessments to be performed daily on days 1-7 and on day of discharge.

On post-operative **<u>day 5</u>**, in addition to the routine blood tests carried out for post-operative assessment, the translational blood samples will be taken, which should be sent directly to Liverpool for central review.

The routine blood tests (FBC, serum chemistry and clotting screen) may be taken within +/-2 days of day 5 and -2 days of day of discharge.

The translational blood samples, however, may only be taken within +2 days of day 5.

8.1.5 Assessments during the follow up phase

Planned study visits will take place 3, 6 and 12 months post-surgery and the assessments outlined in table above performed.

Patients will be followed up in the surgical outpatient clinic as part of their routine post-surgical assessment. The assessments in this study will be built into these appointments so as not to over burden patients with visits. If patients are unable to attend the visit in person then it is acceptable for the research nurse to conduct a telephone consultation, with the patients consent, in order to complete the CRF and ensure quality of life and health economics data is collected.

8.1.6 Overall Survival / End of Study

Patients will be followed up until the end of the trial for overall survival only.

Death and any other reasons for ending the trial must be documented on the End of Study CRF.

8.2 Procedures for assessing Efficacy

Patients will be assessed post operatively to determine their progress following surgery. Post-operative assessments will consist of the following:

8.2.1 Presence or absence of post-operative pancreatic fistula (POPF)

POPF are defined as any abnormal connection between the pancreatic duct epithelium and another epithelised surface, which contains pancreatic derived, enzyme rich fluid. POPF need to satisfy the following criteria:

- Output of a measurable volume of fluid via either a drain placed per-operatively or via subsequent placed interventional drain.
- On or after post-operative day 3
- Amylase content greater than 3 times the institutional upper limit of normal.

All classes of POPF will be recorded on post-operative days 3-7, day of discharge and at 3 month follow up. Following clinical recovery POPF will be graded according to Bassi criteria. Please refer to Appendix D, table 2.

8.2.2 Entry into programs of adjuvant therapy/clinical trials of adjuvant therapy

The following will be recorded during the follow up phase at 3, 6 and 12 months post-surgery:

- Entry into any regime or clinical trial involving adjuvant therapy (chemotherapy, radiotherapy, combination therapy or other therapy).
- Time from surgery to entry into a regime or clinical trial involving adjuvant therapy.
- The rate of completion of adjuvant therapy.

8.2.3 Mortality rate and Overall Survival

Death due to any cause during the study period (from date of randomisation to end of trial) will be recorded; 30 and 90 day mortality rates will be reported. Patients will be followed up for overall survival only past 12 month follow up until the end of the trial. Overall Survival will be calculated from the day of surgery until death due to any cause.

8.2.4 Rate of delayed gastric empting

Delayed gastric empting (DGE) is the inability to resume a normal diet by the end of post-operative day 7 and requires prolonged naso-gastric drainage.

Delayed gastric empting (DGE) will be assessed during post-operative days 1 to 7and at discharge. DGE will be graded as A, B or C [36]. Please refer to Appendix D, table 3.

8.2.5 Rate of wound infections

The presence of surgical site infections (SSI) will be assessed post operatively at hospital discharge and at 3 month follow up. These will be graded in accordance with Clavien Dindo Classification of Surgical Complications (see Appendix D, table 1) and divided into superficial and deep incisional SSIs according to the CDC definition [37].

SUPERFICIAL INCISIONAL SSI MUST MEET THE FOLLOWING TWO CRITERIA:

Occur within 30 days of procedure and involve only the skin or subcutaneous tissue around the incision. Plus at least one of the following criteria:

- Purulent drainage from the incision
- Organisms isolated from an aseptically obtained culture of fluid or tissue from the incision
- At least one of the following signs or symptoms of infection – pain or tenderness, localized swelling, redness or heat – and the incision is deliberately opened by a surgeon, unless the culture is negative
- Diagnosis of superficial incisional SSI by a surgeon or attending physician.

DEEP INCISIONAL SSI MUST MEET THE FOLLOWING THREE CRITERIA:

Occur within 30 days of procedure (or one year in the case of implants), are related to the procedure and involve deep soft tissues, such as the fascia and muscles. Plus at least one of the following criteria:

- Purulent drainage from the incision but not from the organ/space of the surgical site
- A deep incision spontaneously dehisces or is deliberately opened by a surgeon when the patient has at least one of the following signs or symptoms – fever (>38°C), localized pain or tenderness – unless the culture is negative
- An abscess or other evidence of infection involving the incision is found on
  direct examination or by histopathologic or radiological examination
- Diagnosis of a deep incisional SSI by a surgeon or attending physician.

A stitch abscess does not qualify an SSI. Infection that involves both superficial and deep incision sites is classified as deep incisional SSI.

8.2.6 Rate of pulmonary infection

Defined as: Infection of the lung with either evidence of increased infection parameters (CRP >2 mg/dl and/or Leukocytes > 10 000/ml) which are not caused by a different pathologic process or evidence of pulmonary infiltration in the chest x-ray, requiring antibiotic therapy.

The presence of post-operative pulmonary infection will be assessed post operatively at hospital discharge and in follow up at 3 months post-surgery. This should be graded in accordance with Clavien Dindo Classification of Surgical Complications (see Appendix D, table 1).

8.2.7 Rate of post-operative fluid collections

This will relate to any internal fluid collection observed post-operatively as an inpatient and in follow up at 3 months post-surgery.

8.2.8 Operation time

Time from incision to closing of the wound will be recorded on the day of surgery CRF.

8.2.9 Rate of intra and post-operative bleeding

Intra operative bleeding will be assessed by a surgeon and anaesthetist and recorded in millilitres during the surgery.

On post-operative days 1 to 7 any bleeding will be graded using the classification of post pancreatectomy haemorrhage [40] and any intervention will be recorded. Please refer to Appendix D, table 4.

The start date and cause of any further bleeding will be reported at discharge.

8.2.10 Rate of re-operation

Re-operation at any point post operatively will be recorded.

8.2.11 Rate of venous thrombo-embolism

The presence of venous thrombo-embolism will be assessed post operatively at hospital discharge and at 3 month follow up. This should be graded in accordance with Clavien Dindo Classification of Surgical Complications (see Appendix D, table 1).

8.2.12 Hospital stay

Total hospital stay in days following surgery will be recorded at discharge. Any readmission related to the surgery will be recorded at during the follow up phase.

8.2.13 Pancreatic endocrine insufficiency status

This will be assessed at enrolment prior to surgery and then post-operatively as an inpatient and throughout the follow up phase.

8.2.14 Diabetic status

Patients diabetic status following surgery will be assessed at discharge and then throughout the follow up phase.

8.3 Procedures for Assessing Safety

Safety will be assessed through the reporting of post-operative complications as described in section 11. Assessments of end point complications and all other complications will be performed at post-surgical study visits as described in section 8.1. All post-operative complications that occur from time of surgery up to 12 month follow up will be reported.

Post-operative complications that meet the definition of serious will be reported from time of surgery up to 6 months post-surgery. Please refer to section 11 for details of exceptions to SAE reporting. SAEs may be reported past 6 months post-surgery if deemed appropriate to do so by the local investigator, e.g. the complication is considered to be related to the trial surgery.

Post-operative complications will be described in accordance with the Medical Dictionary for Regulatory Activities (MedDRA) version 12.

Non serious complications will be graded using the Clavien-Dindo Classification of Surgical Complications [41] (see Appendix D, table 1). Non serious occurrences of POPF, delayed gastric emptying and bleeding however each will be graded using the corresponding grading systems described in sections 8.2.1, 8.2.4 and 8.2.9 (Please refer to Appendix D, table 2, 3 and 4).

Post-operative complications that meet the definition of serious including POPF, delayed gastric emptying and bleeding will all be graded using the Clavien Dindo Classification of Surgical Complications for consistency.

8.4 Other Assessments

8.4.1 Quality of Life and Health Economics

Quality of life will be assessed with the EORTC Quality of Life Questionnaire (QLQ-C30) version 3 (Appendix B) pre operatively, post operatively and in follow up at 3, 6 and 12 months post surgery. This is a generic cancer instrument composed of multi-item and single scales. These include five functional scales (physical, role, emotional, social and cognitive function), three symptom (fatigue, nausea and vomiting and pain) and a global health status/QL scale and six single items (dyspnoea, insomnia, appetite loss, constipation, diarrhoea and financial difficulties). All scales and single items meet the required standards for reliability and validity.

Patients are eligible for the QoL assessment in this study if they fulfil the eligibility criteria and complete the baseline QoL questionnaires before randomisation. Patients will be informed in the patient informed consent form that they will have their QoL assessment regularly while involved in this trial. Patients will be asked to fill out the questionnaires as completely and accurately as possible. The average time to complete the entire questionnaire is approximately 10-15 minutes. The clinical forms will include a question whether the QL forms have been filled in -and if not, the reason why. Data will be scored according to the algorithm described in the EORTC QLQ-C30 scoring manual. All scales and single items are scored on categorical scales and linearly transformed to 0-100 scales where: A high score for a symptom scale or item represents a high level of symptoms or problems. A high score for a functional scale represents a high or healthy level of functioning and a high score for the global health status/QL represents high QL.

In addition, health economic benefit will be assessed with a generic preference-based tool, the EQ-5D and a disease specific tool, the EORTC QLQ-C30, both pre-operatively and post-operatively and then at 3, 6 and 12 months when follow-up is completed. This will generate a QoL profile of each of the patient groups. The sensitivity of each of the tools to the quality of life changes experienced by the patients will be explored and combined with any variation in 12 months survival to generate quality adjusted life years, where appropriate. The variation in costs between the two techniques will be calculated from individual patient resource use information on the length of operation, the length of stay within each ward type, and any non-surgical events. These two elements can be combined and thus the Blumgart Technique can be evaluated for its cost-effectiveness in comparison to the Cattell-Warren Anastomosis method.

The economic outcomes (quality of life and costs) are secondary outcomes and the trial will not be powered to detect them, but powered according to the primary clinical outcome. The comparative outcomes of different sub-groups of patients will also be explored to assess the cost- effectiveness of the Blumgart Technique for these groups. Some elements of this analysis may have to be hypothesis generating, as the sample size within sub-groups is unlikely to be large enough to generate significant differences. However the benefit of doing such sub-group identification and analysis is that interesting groups, which warrant further analysis and focus, may be highlighted.

8.4.2 Special Assays or Procedures

Operative Photographs
Operative photographs will be taken at the 3 crucial steps of anastomosis formation: 1) Preparation of the pancreatic neck; 2) Insertion of the pancreatic parenchymal and pancreatic duct to jejunal mucosa stiches and 3) The finished anastomosis.

The crucial steps were discussed during the consensus meeting and formalised following the pilot phase of the study in the operative manual.

Photographs of the 3 crucial steps should be uploaded onto the LCTU portal immediately after the surgery has been completed. If photographs are not taken for whatever reason, the surgeon must log this on the photograph upload system on the LCTU portal and on the CRF.

The photographs will be reviewed by the Chief Investigator and a second reviewer for consistency. Both reviewers will remain blinded to the patients randomisation therefore it is essential that no information relating to the type of anastomosis performed or patient identifiers (including trial number) are included in the document name or on the photographs themselves when uploaded to the LCTU.

8.5 Substudies

The following translational samples will be collected for the PANasta trial. These samples will be in addition to the routine blood samples taken for local analysis. Detailed instructions on the collection, storage and transportation of samples will be distributed to each site that registers for the study.

Blood samples
One 10ml blood sample in an EDTA tube and one 8.5ml blood sample in an SST tube will be taken from all patients at the following time points:

1. Pre-operatively, either on the day before or morning of surgery dependent upon individual site preferences.
2. Post-operatively on post-operative day 5 (+2 days).

Blood samples will be sent to the University of Liverpool GCLP laboratory in the kits provided when requested.

These samples will be stored after the trial ends for future research.

Histological Slide
One histological slide of the pancreatic neck transection margin will be requested from each patient to assess the amount of fibrosis. An en face section will be required and the diagnostic H&E slide will be requested for central pathology review.

The LCTU will request the H&E stained diagnostic pancreatic neck transection margin slides for all patients at each site on a 6 monthly basis. An anonymised copy of the histopathology report should be sent with each patient's slide.

The central pathology review will be carried out by Professor Fiona Campbell, Consultant Gastrointestinal Pathologist, who will store all of the histology slides for the duration of the trial. At the end of the trial, each patient's histology slide will be returned to the original Pathology Department.

Histology slides (and the accompanying histopathology reports) requested by the LCTU for central review should be sent to:

Professor Fiona Campbell
Consultant Gastrointestinal Pathologist
Department of Pathology
5th Floor Duncan Building
Royal Liverpool University Hospital
Daulby Street
Liverpool
L69 3GA

The amount of fibrosis in the diagnostic H&E stained section of the pancreatic neck transaction margin will be assessed morphologically. The results will be compared with anastomosis outcome to determine if there is any correlation.

8.6 Loss to Follow-up

If any of the trial patients are lost to follow up, contact will initially be attempted through the PI at each centre. If the PI at the trial centre is not the patient’s usual clinician responsible for their speciality care then follow-up will also be attempted through this clinician. Where all of these attempts are unsuccessful, the patient’s GP will be asked to provide follow-up information to the recruiting centre.

8.7 Trial Closure

Investigators will be informed when patient recruitment is to cease.

Trial enrolment may be stopped at a site when the total number of patients for the trial has been obtained.

The trial will close once all the subjects that have been randomised have completed one year of post-surgical follow up and all centres have completed and returned all necessary CRF’s.

The Independent Safety and Data Monitoring Committee (ISDMC) may recommend to the Trial Steering Committee (TSC) that the trial be stopped prematurely. Such premature termination / suspension of the trial will be notified to the MREC as required.

The trial will be considered formally closed when the database is locked.

9 QUALITY ASSURANCE

9.1 Blinding

Randomisation to treatment arm will be double blinded. The patient and site staff will be blinded. The surgeon will be the only individual at site who will know what treatment a patient is randomised to. In order to maintain the blind for the patient and other site staff, the surgeon will not include what anastomosis has been performed in the surgical notes but rather will record in the notes “anastomosis constructed as per PANasta trial randomisation".

The Chief Investigator will be blinded and will assess SAEs in their blinded form as the expected events for both types of anastomosis are the same. The Chief Investigator and the second reviewer will also be blinded when assessing the operative photographs for quality assurance. This will be achieved by concealing the patient number and randomisation result.

The LCTU will not be blinded.

9.2 Pilot

This study is ambitious intending to randomise over 500 patients undergoing pancreatic surgery. These numbers are powered to detect a 10% difference in the end point of postoperative pancreatic fistula – which is a relevant difference in surgical trials. Included is a generous dropout rate and takes account of patients who are not randomised if unrespectable disease is encountered at laparotomy. Following the constructive feedback from CTAAC and after discussion with a surgeon with expertise in trial methodology; a decision to incorporate a feasibility pilot study within the first 6 months of this project has been adopted. This will run in 2 centres initially from the day of the trial opening and will evaluate in step wise fashion: 1) The number of patients eligible at each site; 2) the number of patients enrolled; 3) the number of patients randomised and finally 4) the number of patients who took up the randomization This will inform us of the real figures for stopping this trial for futility in recruitment. If the number enrolled at six months is less than 40 patients, then a decision made whether to continue the trial or not will be made. If rate of recruitment is acceptable then the other sites will be opened, with an expected number of patients at 12 months of 137. The design allows for an interim analysis with possible early stopping after results for 220 patients are available.

The pilot phase concluded on 23rd October 2015. The TSC and DMC deemed the pilot phase a success following review of recruitment data and the trial continues.

9.3 Standardisation of Surgical Procedures

An Operative Manual was formalised after the pilot phase. This is a guide for surgeons on the construction of a Blumgart and a Cattell Warren anastomosis. It is intended to standardise the construction of both types of

anastomoses and classifies key steps for the construction of each anastomosis as:

a. Mandatory to the construction of a safe anastomosis
b. Prohibited for the construction of a safe anastomosis
c. Equivocal steps where options between methods or techniques will be permitted.

9.4 Operative Photographs

During the surgery, operative photographs will be taken at critical stages of anastomosis construction as described in the Operative Manual. Photographs will be centrally reviewed by the CI and a second reviewer to assess quality and ensure consistency. Both reviewers will be blinded.

10 STATISTICAL CONSIDERATIONS

10.1 Method of Randomisation

Randomisation will be performed by surgeons at each centre via the TARDIS randomisation tool and data storage will be controlled by the LCTU. Block randomisation will be implemented for the allocation of treatment arm, with the following stratification factors:

1. Pancreatic texture: soft vs normal/hard
2. Pancreatic duct diameter: normal(≤3mm) vs dilated (>3mm)
3. Research site

Patients will be randomised to treatment arms in the ratio 1:1, just prior to surgical reconstruction of the pancreatic remnant.

10.2 Outcome Measures

10.2.1 Primary

This is the presence or absence of post-operative pancreatic fistula (POPF) as defined by Bassi *et al.* [34]. For the purpose of this study all classes of fistula will be recorded.

10.2.2 Secondary

- Entry into adjuvant therapy/clinical trials of adjuvant therapy
- Mortality Rate
- Overall Survival
- Delayed Gastric Empting
- Rate of wound infections
- Rate of Pulmonary Infection
- Rate of post-operative fluid collections
- Operation time
- Rate of intra and post-operative bleeding
- Rate of re-operation
- Rate of venous thrombo-embolism
- Hospital stay
- Quality of life and health economic assessments

10.3 Sample Size

The primary outcome is the binary measure of pancreatic leak or no leak. Current experience suggests the present rate of leak is 20% in the standard treatment; a reduction to a 10% rate in the trial treatment is of clinical importance. A sample size of 208 patients per arm would allow a two-sided, two sample test for binomial proportions to detect a difference in proportions of 10% (10% vs 20%) with power

80% and significance level 0.05. The sample size calculation was based on a O'Brien-Fleming 2 stage design with binding futility boundaries (SAS PROC SEQDESIGN version 9.3 was used for the design). Noncompliance would occur in the event of un-resectable disease at laparotomy (estimated at 15%) with a further assumed loss to follow up of 3%, giving an overall sample size of 253 patients to be enrolled per arm.

10.4 Interim Monitoring and Analyses

Formal interim statistical monitoring of the accumulating data will be performed at regular intervals (at least annually) for review by an Independent Data Monitoring and Safety Committee (ISDMC). In addition, the design allows for an interim analysis with possible early stopping after results for 208 patients are available. At this stage, based on a O'Brien-Fleming 2 stage design, if the standardized Z value lies in the interval (-0.698, 0.698) then the trial is stopped for "acceptance" of the null-hypothesis of equal proportions. If the standardized Z value is less than -2.736 or greater than 2.736 then the trial is stopped accepting one proportion is significantly less than the other. Otherwise the trial continues to the final stage where the test for the standardized Z value will have "acceptance" region for the null hypothesis of (-1.934, 1.934). Values outside this region will indicate significantly different proportions. (SAS PROC SEQDESIGN version 9.3 was used for the design.) These analyses will be performed at the Liverpool Clinical Trials Unit and reviewed by the ISDMC.

The ISDMC will be asked to give advice on whether the accumulated data from the trial, together with results from other relevant trials, justify continuing recruitment of further patients or further follow-up. A decision to discontinue recruitment, in all patients or in selected subgroups will be made only if the result is likely to convince a broad range of clinicians including participants in the trial and the general clinical community. If a decision is made to continue, the ISDMC will advise on the frequency of future reviews of the data on the basis of accrual and event rates. The ISDMC will make recommendations to the Trial Steering Committee (TSC, see section 16) as to the continuation of the trial.

Early Stopping Rule: The first six months of the study will be run as a feasibility/ pilot study with a decision, whether to continue or not made at that point. Two sites will be opened initially for this feasibility/ pilot phase. The decision whether to continue or not will be based on the number found to be eligible across the two sites, the number enrolled, the number randomised and the number who took up the randomisation. If the number enrolled at six months is less than 40 patients, then a decision will be made whether to continue the trial or not will be made. If rate of recruitment is acceptable then the other sites will be opened, with an expected number of patients at 12 months of 137 across all sites.

10.5 Analysis Plan

The trial will be analysed and reported following the 'CONSORT' guidelines [39]. All statistical analyses will be on an intention to treat basis. Missing data, which are anticipated to mainly affect the quality of life outcome measure, will be handled by considering the robustness of the complete case analysis to sensitivity analyses using different imputation assumptions informed by data collected on reasons for missing data. Continuous variables will be summarised by descriptive

statistics (mean, standard deviation, minimum, median and maximum) and frequency tables will be provided for categorical data.

The two-sided, two sample test for binomial proportions will be used to test for a difference in the probabilities of leak/fistula between the two arms. Sensitivity analysis will be performed using the continuity corrected Cochran-Mantel-Haenszel (CMH) test calculated over the recruiting centres. Logistic regression will be used to investigate the variation in these probabilities by centre. The secondary endpoints will be analysed using summary statistics, CMH tests, logistic regression, analysis of variance and multivariate methods as appropriate. Overall survival is defined as time from randomisation to death from any cause. Patients who do not experience the event of interest will be censored at the date last seen alive. Overall survival will be performed using cox proportional hazards regression. Hazard ratio, 95%Ci and p-value of the cox model will be reported. As the two procedures are standard practice, a formal analysis of the primary endpoint only will be undertaken when half the patients have been recruited. Quality of life measurements will be assessed over time and comparisons made between treatment groups using longitudinal analysis with appropriate recognition for informative dropout.

11 SAFETY

11.1 Terms and Definitions

Adverse Event (AE)
Any untoward medical occurrence [i.e. any unfavourable or unintended sign including abnormal laboratory results), symptom or disease} in a research participant to whom a medicinal / clinical investigation has been administered, including occurrences which are not necessarily caused by or related to that product / investigation.

Surgical complications will be the only events reported to assess safety. Due to the nature of the PANasta trial, all other non-serious and serious adverse events will not be routinely reported.

Generalised signs and symptoms of having undergone major abdominal surgery, such as lethargy, should not be reported.

Surgical Complication
Any deviation from the ideal postoperative course that is not inherent in the procedure and does not comprise a failure to cure (disease or condition that remains unchanged after surgery) [41].

Serious Adverse Event (SAE)
Any adverse event (surgical complication) is classified as serious if it:

a) results in death
b) is life-threatening* (subject at immediate risk of death)
c) requires in-patient hospitalisation or prolongation of existing hospitalisation**
d) results in persistent or significant disability or incapacity, or
e) consists of a congenital anomaly or birth defect
f) Important medical events that may not be immediately life-threatening or result in death or hospitalisation but may jeopardise the patient or may require intervention to prevent one of the other outcomes listed in the definition above should also be considered serious.

*'life-threatening' in the definition of 'serious' refers to an event in which the patient was at risk of death at the time of the event; it does not refer to an event which hypothetically might have caused death if it were more severe.
Hospitalisation is defined as an inpatient admission, regardless of length of stay, even if the hospitalisation is a precautionary measure for continued observation. **Hospitalisations for a pre-existing condition, including elective procedures that have not worsened, do not constitute an SAE.

11.2 Notes on Adverse Event Inclusions and Exclusions

There are trial specific inclusions and exclusions for reporting SAEs for PANasta. These are detailed below.

11.2.1 Include

- Associated symptoms and events that are related to the primary and secondary endpoints of the trial that are Clavien Dindo grade 4 or above (see Appendix D, table 1)
- An exacerbation of a pre-existing illness/condition that is deemed to be related to the trial surgery.
- An increase in frequency or intensity of a pre-existing episodic event/condition /condition that is deemed to be related to the trial surgery.
- A condition (even though it may have been present prior to the start of the trial) detected up to 6 months after surgery.

11.2.2 Do Not Include

- Events including signs, symptoms and disease that are not deemed a complication of the trial surgery as per the definition above.
- Generalised signs and symptoms of having undergone major abdominal surgery.
- Associated symptoms and events related to the primary and secondary end points of the trial that are Clavien Dindo grade 3b or below (see table 5 in section 11.7)
- Extended hospital stay due to a delay in planned surgery.
- In-patient hospitalisation or prolongation of existing hospitalisation due to post-operative complications that are grade 3b or below (unexpected complications).
- Medical or surgical procedures - the condition which leads to the procedure is the SAE.
- Pre-existing disease or conditions present before surgerythat do not worsen.
- An exacerbation of a pre-existing illness/condition that is not deemed to be related to the trial surgery.
- An increase in frequency or intensity of a pre-existing episodic event/condition that is not deemed to be related to the trial surgery.
- Situations where an untoward medical occurrence has occurred e.g. cosmetic elective surgery.
- The disease being treated or associated symptoms/signs unless more severe than expected for the patient's condition.
- Injury or accidents
- Abnormal laboratory results
- Side effects of any medication

11.2.3 Reporting of Pregnancy

Not required.

11.3 Notes on Severity / Grading of Adverse Events (Surgical Complications)

The assignment of the severity/grading should be made by the investigator responsible for the care of the participant using the definitions in the tables below.

Complications must be assessed according to the medical criteria alone using the applicable tables in Appendix D.

11.3.1 Non-serious Adverse Events (Surgical Complications)

In general all non-serious surgical complications should be assessed in accordance with the Clavien Dindo Classification of Surgical Complications. Please refer to Appendix D, table 1.

Non-serious Post-operative pancreatic fistula, delayed gastric emptying and bleeding should be graded using the corresponding grading tables in Appendix D (tables 2, 3 and 4).

11.3.2 Serious Adverse Events (Surgical Complications)

All surgical complications that meet the definition of serious should be assessed in accordance with the Clavien Dindo Classification of Surgical Complications on the SAE Report form for consistency. This includes POPF, delayed gastric emptying and bleeding. The below table describes what grading table should be used.

Table 1: Grading Table Guidance

Complication	Grading table to use
Non-serious Post-operative pancreatic fistula	Post-operative Pancreatic Fistula Grading Table
Serious Post-operative pancreatic fistula	Clavien Dindo Classification of Surgical Complications
Non-serious Delayed Gastric Emptying	Delayed Gastric Emptying Grading Table
Serious Delayed Gastric Emptying	Clavien Dindo Classification of Surgical Complications
Non-Serious Bleeding	classification of post pancreatectomy haemorrhage
Serious Bleeding	Clavien Dindo Classification of Surgical Complications
All other complications ((serious AND non-serious)	Clavien Dindo Classification of Surgical Complications

A distinction is drawn between serious and severe AEs (surgical complications). Severity is a measure of intensity (see above) whereas seriousness is defined using the criteria in section 11.1, hence, a severe AE (surgical complications) need not necessarily be a Serious Adverse Event.

11.4 Relationship to Trial Procedure

The assignment of the causality should be made by the investigator responsible for the care of the participant using the definitions in table 5.

Causality should be assigned to the following:

a. Anaesthetic
b. Generality of surgery
c. Whipples procedure
d. Anastomosis

If any doubt about the causality exists the local investigator should inform the study coordination centre who will notify the Chief Investigators. In the case of discrepant views on causality between the investigator and others, the MREC will be informed of both points of view.

Table 2: Definitions of Causality

Relationship	Description
**None**	There is no evidence of any causal relationship. N.B. An alternative cause for the AE (surgical complication) should be given
**Unlikely**	There is little evidence to suggest there is a causal relationship (e.g. the event did not occur within a reasonable time after the trial procedure). There is another reasonable explanation for the event (e.g. the participant's clinical condition, other concomitant treatment).
**Possibly**	There is some evidence to suggest a causal relationship (e.g. because the event occurs within a reasonable time after the trial procedure). However, the influence of other factors may have contributed to the event (e.g. chemotherapy or other concomitant treatments).
**Probably**	There is evidence to suggest a causal relationship and the influence of other factors is unlikely.
**Highly Probable**	There is clear evidence to suggest a causal relationship and other possible contributing factors can be ruled out.

11.5 Expectedness

Expectedness will be assessed against the following:

a. Whipples procedure
b. Anastomosis

Post-operative complications of pancreatic surgery (Whipples procedure and/or anastomosis) that are Clavien Dindo grade 3b or below are **expected** for the PANasta trial.

Post-operative complications of pancreatic surgery (Whipples procedure and/or anastomosis) that are Clavien Dindo grade 4 and above are **unexpected** for the PANasta trial.

An AE (surgical complication) where the causal relationship to the study procedure (Whipples procedure and/or anastomosis) is assessed by the investigator as “possible”, “probable”, or “highly probable”, is graded as serious and **unexpected** is subject to expedited reporting to the Research Ethics Committee (REC). This is the responsibility of the LCTU.

11.6 Follow-up After Adverse Events (Surgical Complications)

All adverse events (surgical complications) should be followed until satisfactory resolution or until the investigator responsible for the care of the participant deems the event to be chronic or the patient to be stable.

When reporting SAEs the investigator responsible for the care of the participant should apply the following criteria to provide information relating to event outcomes: resolved; resolved with sequelae (specifying with additional narrative); not resolved/ongoing; ongoing at final follow-up; fatal or unknown.

11.7 Reporting Procedures

Depending on the nature of the event the reporting procedures below should be followed. Any questions concerning adverse event reporting should be directed to the LCTU in the first instance.

11.7.1 Non serious AEs (Surgical Complications)

All non-serious expected and unexpected complications of surgery should be reported from the day of surgery at each post-operative study visit and throughout the follow up phase. All complications should be reported on the appropriate CRF and graded using the Clavien Dindo Classification of Surgical Complications unless otherwise specified (POPF, DGE and bleeding).

11.7.2 Serious AEs (Surgical Complications)

AEs (Surgical Complications) that meet the definition of serious must be reported as an SAE.

SAEs should be reported from the day of surgery up to 6 months post-surgery. SAEs may be reported past 6 months post-surgery if deemed appropriate to do so by the local investigator, e.g. the complication is considered to be related to the trial surgery.

Grade 4 and above primary and secondary endpoint complications and all other complications regardless of grade that meet the definition of serious MUST be reported as an SAE.

Grade 3b and below primary and secondary endpoint complications and associated symptoms that meet the definition of serious are exempt from SAE reporting. Such events should only be reported in the relevant section of the CRF.

Please refer to the table below for complications/events exempt from SAE reporting.

Table 5: Complications Exempt from SAE Reporting

**Primary and Secondary End Points including associated symptoms**	**Grade 3b and below**	**Grade 4 and above**
	**Report as SAE**	**Report as SAE**
Pancreatic fistula	No	Yes
Delayed gastric emptying	No	Yes
Wound infections	No	Yes
Pulmonary Infection	No	Yes
Post-operative fluid collection	No	Yes
Intra and post-operative bleeding	No	Yes
Venous thromboembolism	No	Yes
Interventional drainage procedures	No	Yes
Surgical complication related hospital stay	No	Yes

All SAEs must be reported within **24 hours** of the local site becoming aware of the event by recording the information directly onto a hard copy Serious Adverse Event form. The SAE form asks for the nature of event, date of onset, severity, corrective therapies given, outcome and causality. The responsible investigator should sign the causality of the event. Additional information should be sent within 5 days if the event has not resolved at the time of reporting.

The LCTU will notify the main REC of all SAEs that are confirmed as being **related** to the trial surgery(Whipples procedure and or anastomosis) and **unexpected** occurring during the study within 15 days of being notified of the event. All investigators will be informed of all expedited SAEs occurring throughout the study. Local investigators should report any SAEs as required by their Research & and Development Office.

Steps for reporting - PAPER SAE FORMS

i. The SAE form should be completed by the responsible investigator i.e. the consultant named on the 'signature list and delegation of responsibilities log' who is responsible for the patient's care. The investigator should assess the SAE for the likelihood that it is a response to the trial intervention. In the absence of the responsible investigator the form should be completed and signed by a designated member of the site trial team and faxed to the LCTU immediately. The responsible investigator should check the SAE form, make changes as appropriate, sign and then re-fax to the LCTU as soon as possible. The initial report shall be followed by detailed, written reports.
ii. Send the SAE form by fax (within 24 hours or next working day) to the LCTU

Fax Number: 0151 794 8930

iii. The responsible investigator must **notify** their R&D department of the event (as per standard local procedure).

iv. In the case of an SAE the subject must be followed-up until clinical recovery is complete and laboratory results have returned to normal, or until the event has stabilised. Follow-up may continue after completion of protocol treatment if necessary.
v. Follow-up information is noted on another SAE form by ticking the box marked 'follow-up' and faxing to the LCTU as information becomes available. Extra, annotated information and/or copies of test results may be provided separately.
vi. The patient **must** be identified by trial number, date of birth and initials only. The patient's name **should not** be used on any correspondence.

On reporting an SAE to the LCTU (by either method), research sites will receive an acknowledgement of receipt, either by email or fax. If a receipt has not been received within 2 hours of reporting the SAE, please telephone the PANasta team on 0151 795 5266.

The Investigator must institute appropriate therapeutic action and follow-up measures in accordance with Good Medical Practice but should notify the study co-ordinator of such actions.

The minimum dataset required for a preliminary report should include the following.

- Research subject trial number and initials.
- Date of onset of event.
- Brief description of event and CTCAE (v4) grade.
- Causality relationship.
- Dated signature of investigator/co-investigator and clearly printed name. Date of last administration of study drug.
- Causality relationship.
- Dated signature of investigator/co-investigator and clearly printed name.
- Definition of serious

11.7.3 Maintenance of Blinding

Systems for SAE reporting should, as far as possible, maintain blinding of individual clinicians and of trials staff involved in the day-to-day running of the trial. Unblinding clinicians may be unavoidable if the information is necessary for the medical management of particular patients. The safety of patients in the trial always takes priority. In each report, seriousness, causality and expectedness should be evaluated for each trial intervention unless criteria have been fulfilled (section 7.4) and unblinding has taken place.

As the expected events for both surgical interventions in this trial are the same, there is no requirement for SAEs to be unblinded by the clinical trials unit prior to the clinical co-ordinators evaluation of expectedness and reporting to MREC.

11.8 Responsibilities – Investigator

The Investigator is responsible for reporting all AEs that are observed or reported during the study in accordance with section 11.7

All SAEs must be reported immediately by the investigator to the LCTU on an SAE form unless the SAE is specified in the protocol as not requiring immediate reporting (please refer to sections 11.2 and 11.5). All other adverse events should be reported on the regular progress/follow-up reports.

11.9 Responsibilities – CR:UK LCTU

The LCTU is undertaking duties delegated by the trial sponsor, University of Liverpool, and is responsible for the reporting of SAEs to the main REC within 15 days of becoming aware of the event. A list of all SAEs that occur will also be reported annually.

It is recommended that the following safety issues should also be reported in an expedited fashion

- An increase in the rate of occurrence or a qualitative change of an expected serious adverse events, which is judged to be clinically important;
- Post-study SAEs that occur after the patient has completed a clinical trial and are notified by the investigator to the sponsor;
- New events related to the conduct of the trial and likely to affect the safety of the subjects, such as a serious adverse event which could be associated with the trial procedures and which could modify the conduct of the trial;
- Recommendations of the Data Monitoring Committee, if any, where relevant for the safety of the subjects.

Staff at the LCTU will liaise with the designated Clinical Co-ordinator who will evaluate all SAEs received for seriousness, expectedness and causality. The causality assessment given by the Local Investigator at the hospital cannot be overruled and in the case of disagreement, both opinions will be provided with the report.

The LCTU will also send an annual safety report containing a list of all SAEs to MREC.

Patient safety incidents that take place in the course of research should be reported to the National Patient Safety Agency (NPSA) by each participating NHS Trust in accordance with local reporting procedures.

12 ETHICAL CONSIDERATIONS

12.1 Ethical Considerations

Ethical review of the study is a legal requirement to safeguard the rights, dignity and welfare of people participating in research. Amendments made to the study after a favourable ethical and regulatory opinion will be submitted and approved prior to implementation. The requirement for ethical and regulatory authority approvals applies to all participating countries.

Each participating Principal Investigator (PI) will be named on the original ethics application form or on a subsequent substantial amendment.
Written evidence of NHS R&D approval must be made available to the CR-UK Liverpool Cancer Trials unit prior to randomisation of subjects at site.

12.2 Ethical Approval

The trial protocol has received the favourable opinion of the Multi-centre Research Ethics Committee (MREC) but all participating sites must undergo site specific assessment via the IRAS (Integrated Research Application System). A copy of all site approval documents and a copy of the PIS and ICF on local headed paper should be forwarded to LCTU before patients are entered. The LCTU should receive notification of positive SSA for each new centre via the site's R&D department

12.3 Informed Consent Process

Informed consent is a process initiated prior to an individual agreeing to participate in a trial and continues throughout the individual's participation. Informed consent is required for all patients participating in LCTU coordinated trials. The consent process must be carried out by a medically qualified member of the research team. In obtaining and documenting informed consent, the investigator should comply with applicable regulatory requirements and should adhere to GCP and to the ethical principles that have their origin in the Declaration of Helsinki.

Discussion of objectives, risks and inconveniences of the trial and the conditions under which it is to be conducted are to be provided to patients by staff with appropriate experience. Appropriate Patient Information and Consent forms, describing in detail the trial interventions, trial procedures and risks will be approved by an independent ethics committee (IEC) and the patient will be asked to read and review the document. Upon reviewing the document, the investigator will explain the research study to the patient and answer any questions that may arise. A contact point where further information about the trial may be obtained will be provided.

After being given adequate time to consider the information, the patient will be asked to sign the informed consent document. A copy of the informed consent document will be given to the patient representative for their records and a copy placed in the medical records, with the original retained in the Investigator Site File.

The patient may withdraw from the trial at any time by revoking the informed consent. The rights and welfare of the patients will be protected by emphasising to them that the quality of medical care will not be adversely affected if they decline to participate in this study.

12.4 Study Discontinuation

The chief investigator can prematurely close this trial after consultation with the steering committee. If termination occurs the steering committee will discuss this issue with the independent Data Safety Monitoring Board. The local ethics committee's will be informed. Reasons for trial termination include:

- The incidence or severity of SAE's/morbidity in this trial indicates a potential health hazard caused by the study treatment.
- It appears that patient's enrolment is unsatisfactory with respect to quality and/or quantity or if data recording is severely inaccurate and/or incomplete.
- External evidence demanding trial termination.

13 REGULATORY APPROVAL

This trial has not been registered with the MHRA. No CTA is required for surgical trials.

14 TRIAL MONITORING

Site monitoring is conducted to ensure protection of patients participating in the trial, trial procedures, laboratory, trial intervention administration, and data collection processes are of high quality and meet sponsor and, when appropriate, regulatory requirements. A risk assessment will be carried out to determine the level of monitoring required, and a subsequent monitoring plan will be developed to document who will conduct the site monitoring, at what frequency monitoring will be carried out and the level of detail at which monitoring will be conducted.

Risk Assessment

In accordance with LCTU Standard Operating Procedures and the requirements of the sponsor organisation a study risk assessment has been completed in partnership with:

- Representatives of the trial sponsors (University of Liverpool)
- Chief Investigator
- Trial Co-ordinator
- Trial Statistician
- LCTU Operational Director
- LCTU Senior Management Team

In conducting this risk assessment, the contributors considered potential patient, organisational and study hazards, the likelihood of their occurrence and resulting impact should they occur.

The outcome of the risk assessment is categorised based upon the potential risk associated with the trial intervention in accordance with MRC/DH/MHRA Project on Risk-adapted Approaches to the Management of Clinic Trials of Investigational medicinal Products
http://www.mhra.gov.uk/home/groups/l-ctu/documents/websiteresources/con111784.pdf.
based on the following categories:

- Type A: No higher than that of standard medical care
- Type B: Somewhat higher than that of standard medical care
- Type C: Markedly higher than that of standard medical care
- Non-CTIMP

This trial is a Non-CTIMP and the risk categories described above for CTIMPs (type A, B or C) have been applied to the PANasta trial.

As this is a surgical intervention trial of two standard techniques of reconstructing the pancreatic remnant the risk assessment resulted in a trial category of Type A and thus the trial is considered to be low risk.

14.1 Source Documents

Source data is all information, original records of clinical findings, observations, or other activities in a clinical trial necessary for the reconstruction and evaluation of

the trial. Source data are contained in source documents (original records or certified copies) (ICH E6, 1.51).

Original documents, and data records include: hospital records, clinical and office charts, laboratory notes, memoranda, subjects' diaries or evaluation checklists, pharmacy dispensing records, recorded data from automated instruments, copies or transcriptions certified after verification as being accurate and complete, microfiches, photographic negatives, microfilm or magnetic media, x-rays, subject files, and records kept at the pharmacy and laboratory departments involved in the clinical trial (ICH E6, 1.52):62.

In order to resolve possible discrepancies between information appearing in the CRF and any other patient related documents, it is important to know what constitutes the source document and therefore the source data for all information in the CRF. Data recorded in the CRF should be consistent and verifiable with source data in source documents *other* than the CRF (e.g. medical record, laboratory reports and nurses" notes). Each participating site should maintain appropriate medical and research records for this trial, in compliance with ICH E6 GCP, section 4.9 and regulatory and institutional requirements for the protection of confidentiality of subjects.
For data where no prior record exists and which are recorded directly in the CRF (e.g inclusion/exclusion criteria, adverse events and Quality of life questionnaires), the CRF will be considered the **source document**, unless otherwise indicated by the investigator.
In addition to the above, date (s) of conducting informed consent including date of provision of patient information, trial screening number, trial number, study treatment and the fact that the patient is participating in a clinical trial should be added to the patients' medical record contemporaneously.

14.2 Data Capture Methods

Trial data will be captured using paper case report forms with the exception of adverse and serious adverse events, which will be captured electronically.

14.2.1 Case Report Forms

The study case report form (CRF) is the primary data collection instrument for the study. All data requested on the CRF must be recorded. All missing data must be explained. If a space on the CRF is left blank because the procedure was not done or the question was not asked, write "N/D". If the item is not applicable to the individual case, write "N/A". All entries should be printed legibly in black ink. If any entry error has been made, to correct such an error, draw a single straight line through the incorrect entry and enter the correct data above it. All such changes must be initialled and dated. DO NOT ERASE OR WHITE OUT ERRORS. For clarification of illegible or uncertain entries, print the clarification above the item, then initial and date it.

CRF pages will be available for sites to download from the LCTU portal:

http://www.lctu.org.uk

14.3 Monitoring at LCTU

Data stored at LCTU will be checked for missing or unusual values (range checks) and checked for consistency within participants over time. If any such problems are identified, a photocopy of the problematic CRF(s) will be returned to the local site by post or fax for checking and confirmation or correction, as appropriate – any data which are changed should be crossed through with a single line and initialled (see section 13.3.1). The amended version should be returned to LCTU and the site's copy should also be amended. LCTU will send reminders for any overdue and missing data.

14.4 Clinical Site Monitoring

14.4.1 Direct access to data

In order to perform their role effectively, monitors and persons involved in Quality Assurance and Inspection will need direct access to primary subject data, eg patient records, laboratory reports, appointment books, etc. Because this affects the patient's confidentiality, this fact is included on the Patient Information Sheet and Informed Consent Form.

14.4.2 Confidentiality

Individual participant medical information obtained as a result of this study is considered confidential and disclosure to third parties is prohibited. Case report forms will be labelled with patient initials and unique trial screening and/or trial number. Intra-operative photographs will be transferred to the LCTU via the LCTU portal and reviewed by Mr Christopher Halloran of the Institute for Translational Medicine at the University of Liverpool and a second reviewer; Mr Dhanny Gomez of Queens Medical Centre, Nottingham University Hospitals NHS Trust. Photographs will be identifiable by unique trial number only. Histological slides will be transferred to Professor Fiona Campbell in the department of pathology at the Royal Liverpool University Hospital and will be identifiable by unique trial number only. Blood samples will be transferred to the University of Liverpool GCLP laboratory and will be identifiable by unique trial number only.

Consent forms sent to the LCTU as part of the randomisation process may contain patient identifiers for the purpose of monitoring as described in the trial risk assessment. Such information will be stored in secure, locked cabinets.

14.4.3 Quality Assurance and Quality Control of Data

Systems of quality assurance, including all elements described in this protocol have been/will be implemented within relevant institutions with responsibility for this trial. Standard Operating Procedures (SOPs) are implemented to ensure that clinical trials are conducted in compliance with regulatory requirements and Good Clinical Practice. Quality control is applied to each stage of data handling to ensure that data are accurate, reliable and processed correctly.

The PANasta trial investigational sites, facilities, laboratories and all data (including sources) and documentation must be available for GCP audit and inspection by competent authorities (national and foreign) or IEC. Such audits/inspections may take place at any site where trial related activity is taking place) the Sponsor's site(s), CR-UK Liverpool Cancer Trials Unit or at any investigator's site including laboratories, pharmacies, etc.).

The site staff should assist in all aspects of audit/inspection and be fully cognisant of the LCTU communication strategy for multicentre trials. This includes management system for the Green light process, conforming to the total Quality Management System currently operating within the LCTU.

14.5 Records Retention

The investigator at each investigational site must make arrangements to store the essential trial documents, (as defined in Essential Documents for the Conduct of a Clinical Trial (ICH E6, Guideline for Good Clinical Practice)) including the Investigator Trial File, until the Sponsor or the LCTU informs the investigator that the documents are no longer to be retained. In addition, the investigator is responsible for archiving of all relevant source documents so that the trial data can be compared against source data after completion of the trial (eg in case of inspection from authorities).

The investigator is required to ensure the continued storage of the documents, even if the investigator, for example, leaves the clinic/practice or retires before the end of required storage period. Delegation must be documented in writing.
The LCTU undertakes to store originally completed CRFs and separate copies of the above documents for the same period, except for source documents pertaining to the individual investigational site, which are kept by the investigator only.

Essential documents should be retained for at least 5 years after the completion of the trial. These documents should be retained for a longer period however if required by applicable regulatory requirements or by an agreement with the Sponsor. It is the responsibility of the Sponsor to inform the investigator/institution as to when these documents no longer need to be retained.

At the point where it is decided that the trial documentation is no longer required; the Investigator will be responsible for the destruction of all site trial specific documentation and the Sponsor/LCTU will be responsible for the destruction of all trial related materials retained by the Sponsor/LCTU.

Verification of appropriate informed consent will be enabled by the provision of copies of participants' signed informed consent/assent forms being supplied to the LCTU by recruiting centres. This requires that name data will be transferred to the LCTU, which is explained in the PISC. The LCTU will preserve the confidentiality of participants taking part in the study and the University of Liverpool is a Data Controller registered with the Information Commissioners Office.

15 INDEMNITY

PANasta is sponsored by the University of Liverpool and co-ordinated by the LCTU in the University of Liverpool. The University of Liverpool does not hold insurance against claims for compensation for injury caused by participation in a clinical trial and they cannot offer any indemnity. As this is an investigator-initiated study, The Association of the British Pharmaceutical Industry (ABPI) guidelines for patient compensation by the pharmaceutical industry do not apply. However, in terms of liability, NHS Trust and Non-Trust Hospitals have a duty of care to patients treated, whether or not the patient is taking part in a clinical trial, and they are legally liable for the negligent acts and omission of their employees. Compensation is therefore available in the event of clinical negligence being proven.

Clinical negligence is defined as:
"A breach of duty of care by members of the health care professions employed by NHS bodies or by others consequent on decisions or judgments made by members of those professions acting in their professional capacity in the course of their employment, and which are admitted as negligent by the employer or are determined as such through the legal process".

16 FINANCIAL ARRANGEMENTS

This is a non-commercial trial, and no direct payments are available to cover the costs associated with patient recruitment, treatment administration, follow-up visits, data collection or reasonable travel expenses. The trial is funded by Cancer Research UK, consequently having automatic endorsement from the National Cancer Research Network (NCRN) and UK Clinical Research Network (UKCRN). These organisations will be responsible for providing local investigators with the necessary research infrastructure.

17 TRIAL OVERSIGHT COMMITTEES

17.1 Trial Management Group (TMG)

A Trial Management Group (TMG) will be formed comprising the Chief Investigator, other lead investigators (clinical and non-clinical) and members of the LCTU. The TMG will be responsible for the day-to-day running and management of the trial and will meet approximately 3 times a year.

17.2 Trial Steering Committee (TSC)

The Trial Steering Committee will consist of the TMG plus the following members:

Mr Richard Charnley	Independent Chairperson
Professor Terry Jones	Vice Chairperson / Independent Clinician
Jane Warwick	Independent Statistician
Mr Derek O'Reilly	Co-Investigator (Manchester)
Mr Dhanny Gomez	Co-Investigator (Nottingham)
John Richardson	Patient Representative

The role of the TSC is to provide overall supervision for the trial and provide advice through its independent Chairman. The ultimate decision for the continuation of the trial lies with the TSC.

17.3 Independent Data and Safety Monitoring Committee (IDSMC)

The independent Data and Safety Monitoring Committee (IDSMC) will consist of the following independent members:

Dr Adrian Bloor	Chairperson / Expert Clinician
Professor Daniel Hochhauser	Vice Chairperson / Expert Clinician
Andre Lopes	Independent Statistician

The ISDMC will be responsible for reviewing and assessing recruitment, interim monitoring of safety and effectiveness, trial conduct and external data. The ISDMC will first convene prior to trial opening and will then define frequency of subsequent meetings (at least annually). Details of the interim analysis and monitoring are provided in section 9.

The ISDMC will provide a recommendation to the Trial Steering Committee concerning the continuation of the study.

18 PUBLICATION

The results from different centres will be analysed together and published as soon as possible. Individual Clinicians must undertake not to submit any part of their individual data for publication without the prior consent of the Trial Management Group.

The Trial Management Group will form the basis of the Writing Committee and advise on the nature of publications. The Uniform Requirements for Manuscripts Submitted to Biomedical Journals (http://www.icmje.org/) will be respected. All publications shall include a list of participants, and if there are named authors, these should include the trial's Chief Investigator(s), Statistician(s) and Trial Manager(s) involved at least. If there are no named authors (i.e. group authorship) then a writing committee will be identified that would usually include these people, at least. The ISRCTN allocated to this trial should be attached to any publications resulting from this trial.

The members of the TSC and IDSMC should be listed with their affiliations in the Acknowledgements/Appendix of the main publication.

19 PROTOCOL AMENDMENTS

19.1 Version 1 (22.09.2014)

Original submitted version

19.2 Version 2 (06.10.2014)

Typos amended on page 4.

19.3 Version 3 (10.10.2014)

Clarification on who is blinded and unblinded in section 7.4 and 9.1
Clarification on AE and SAE reporting in section 8.3 and 11.
Additional guidance added re conmeds in section 7.6
Guidance re the removal of surgical drains added to section 7.7.
Guidance re the use o stents added to section 7.8.

19.4 Version 4 (05.10.2015)

General administrative changes

- Cattal-Warren corrected to Cattel-Warren in title on first page
- Contact details of the TSC Chair added
- Data Manager contact details updated
- Reference to MHRA removed from section 9.3.
- Wording of eligibility criteria point 1 altered.
- In protocol summary (section 1) wording of sample size amended from 'powered to detect a 10% difference in primary endpoint' to 'powered to detect a 10% absolute difference in primary endpoint'.

Schematic of Study Design

- Flow diagram updated to remove fresh pancreatic tissue

Pilot Study

- Number of patients required to continue the trial after the pilot phase clarified and changed to 40 (section 9.2 and 10.4).

Secondary endpoints

- Table of delayed gastric emptying added to section 4.3.3.1
- Time points pulmonary infection is assessed updated in section 4.4.4.2 and 8.2.6.
- Time points post-operative fluid collections are assessed updated in section 4.4.4.3 and 8.2.7.
- Details of how intra and post-operative bleeding will be assessed and time points updated in section 4.4.4.5 and 8.2.9
- Time points added for rate of reoperation 4.4.4.6.
- Time points added for rate of venous thrombo-embolism in section 4.4.4.7.

Withdrawal from Trial Intervention

- Clarification regarding withdrawal of consent and all trial data added to section 5.3.3.

Enrolment and Baseline

- Smoking and alcohol status and family history added to the list of assessments to be completed at baseline in section 6.2.
- Reference to consultee ICF removed from section 6.2.

Trial Treatments

- New section added for use of octreotide, which stipulates it must be administered after surgery at 100ug (section 7.6).
- Additional information added around the use of surgical drains in section 7.7.
- Section added on the use of pancreatic duct stents (section 7.8).

Assessments and procedures

- Schedule of trial procedures table updated to include smoking and alcohol status, family medical history, pancreatic endocrine insufficiency status, diabetic status, CA19-9, octreotide review, surgical drain review, upload of operative photographs, End of study, removal of sequelae of POPF and time points of some assessments updated.
- Surgical drain fluid assessment in schedule of trial procedures table changed to post-operative fluid collection.
- Clarification added that at discharge and throughout follow up weight alone needs to be recorded added to table footer in section 8.1.
- Details of blood tests to be carried out for FBC, serum chemistry and clotting screen added to table footer in section 8.1.
- Guidance on when operative photographs should be uploaded added to to table footer in section 8.1.
- Guidance on how death should be reported added to table footer in section 81.
- Clarification added that no data collection is required at screening in section 8.1.1.
- Reference to surgical tissue sample to be taken from Liverpool patients removed from section 8.1.3.
- Details of pancreatic endocrine insufficiency status and diabetic status added to section 8.2.

Adverse Events

- Reporting period for AEs amended in section 8.3 and 11.7.
- Reporting of AEs in accordance with medDRA v.12 changed from CTCAE v.4 in section 8.3.
- Table of the Clavien-Dindo Classification of Surgical Complications added to section 8.3.
- Clarification added that abnormal laboratory results do not require AE reporting in section 11.2.2.
- Definitions of severity updated and table added to section 11.3.

Operative Photographs / Blinding

- Clarification added that both the CI and independent reviewer will be blinded when reviewing operative photographs (section 8.4.2, 9.1 and 9.4)

Sub studies

- Removal of surgical tissue sample to be taken from Liverpool cohort in section 8.5 and 14.4.2.

- Removal of suggested tests to be performed in section 8.5.

References

- References for the Clavien-Dindo Classification of Surgical Complications and Classification of Post Pancreatectomy Haemorrhage added.

19.5 Version 5 (11/05/2015)

Study flow chart updated

Eligibility Criteria

- Inclusion criteria updated to include 18 years and above.
- Women unable or unwilling to use contraception from time of consent up to the day of surgery added as exclusion criteria.

Co-enrolment Guidelines

- Wording amended to allow patients to be co-enrolled on other pancreatic surgery trials at the discretion of the CI.
-

Blinding

- Clarification of who is blinded, CI is blinded.

Octreotide

- Clarification added that the initial dose of octreotide may be omitted if patient is admitted on the day of surgery in accordance with local practice.
- Clarification added about the days on which octreotide should be administered.

Assessments

- Clarification added that informed consent does not need to be within 4 weeks of surgery.
- Removal of INR from FBC.
- Diabetic status at baseline added to the table of assessments (previously omitted in error)
- Addition of ±2 day window for day 5 routine blood tests.
- Addition of +2 day window for day 5 translational blood sample.
- Addition of -2 day window for day of discharge blood sample.
- Addition of common symptoms and relevant tests to be recorded at baseline.
- Addition of details of surgery (previously omitted In error).
- Window for eligibility and enrolment assessments extended from 2 weeks to 4 weeks removing the requirement for a discretionary waiver from the CI if done over 2 weeks.Operative photo upload moved from discharge to day of surgery (should now be uploaded immediately after surgery).

Statistical Considerations

- Sample size amended in section 10.3 and 10.4 following clarification of the original sample size calculation.

Safety Reporting

- Removal of laboratory anomalies in adverse event reporting inclusions.
- AE inclusions updated to include events related to primary and secondary endpoints that are grade 4 and above.
- AE exclusions updated to clarify that grade 3 and below events related to primary and secondary endpoints should not be reported as an SAE.
- Table 3 in section 11.5 updated to include differences in reporting depending on the grade of an event.
- Clarification around the reporting timeframes added for both post-operative complications and all other adverse events in section 11.7.
- Removal of reference to the MACRO PV system as not being used for this trial.

Operative Photographs

- Change in second photograph reviewer from Rajesh Yagati Satchidanand to Dhanny Gomez.
- Removal of 'independent' as both reviewers are not independent of the trial.

19.6 Version 6 (22/03/2016)

Cover Page

- Addition of IRAS reference

UK Registration

- Removal of reference to CTA approval. This was left in the protocol in error.

Contact details

- TSC patient rep changed from Dennis Helsby to John Richardson

Section1: Protocol Summary

- Number of sites changed from 'approximately 7' to '7+' UK centres.
- Overall survival added as a secondary endpoint.
- Schematic of study design updated; post-operative follow up changed from 'days 3-7' to 'days 1-7'.

Section 2.2: Rationale

- Typographical errors amended; 'anastomatic' corrected to 'anastomotic'.

Section 4.2: Pilot

- New wording added to explain the pilot phase is complete.

Section 4.3: Primary Endpoint

- Description of primary end point condensed as was duplicated in section 8.

Section 4.4: Secondary Endpoints
- Description of secondary end point condensed as was duplicated in section 8.
- Overall survival added as a secondary endpoint.

Section 5.3: Transfer and Withdrawal
- Removed reference to 'adverse events' and replaced with 'curpical compilations'.

Section 5.3.2 Withdrawal from Trial Intervention
- Wording updated to clarify that withdrawal from the trial prior to randomisation should be reported on the screening log. An EOS form does not need to be completed.

Section 6.1: Screening
- In the third paragraph *"Screening and Enrolment Log"* has been amended to *"Screening Log"*

Section 6.2: Enrolment / Baseline
- New instruction added to allow baseline assessments that are done as part of routine pre-surgery assessment completed before patient has consented.
- New wording added to point 9 of baseline assessments - *(QLQ-C30 and EQ5D)*
- New wording added to explain what happens when patient has been enrolled. This has been adapted from the 'Randomisation' section.
- Statement regarding the transfer of data via Datanywhere added.

Section: 6.3 Randomisation
- Section re-worded to flow better.

Section 7.6: Octreotide
- New wording added to explain that if a patient id discharged before day 6, octreotide may be discontinued in line with hospital discharge.
- New wording added to explain that it may be necessary to continue with Octreotide past post-operative day 6 in accordance with local practice.

Section 8.1: Schedule of Trial Procedures
- Table of trial procedures split out into smaller tables; pre-surgery, day of surgery, post-operative in-patient review and follow up and end of study.
- Removal of AE assessments.
- 'Unexpected complications' changed to 'other complications (not endpoints).
- Hba1c added to the full blood count assessments.
- Post-operative day 1 and day 2 reviews added which include octreotide review, survival status, review of other complications, post-operative bleeding assessment and re-operative review.

- Post-operative day 4 and 6 reviews added which include octreotide review, survival status, review of other complications, post-operative bleeding assessment, re-operative review and delayed gastric emptying assessment.
- Assessment of surgical site infections and venous thromboembolism removed from post-operative days 3, 5 and 7. One review will be conducted at day of discharge.
- Assessment of surgical site infections and venous thromboembolism removed from 6 and 12 month follow up.
- Timing of bloods change from evening before or morning of surgery to day before or morning of surgery.

Section 8.1.2: Enrolment Assessments

- Updated to remove wording about obtaining permission before enrolment which was left in the protocol in error after the previous protocol amendment.

Section 8.1.3: Before Surgery / Randomisation

- Duplicated wording of where to send samples to has been removed and reference to section 8.5.
- Additional wording added to explain that where routine bloods are not taken in line with the protocol on the day before or morning of surgery, it is permissible to take pre-operative bloods in accordance with local practice.

Section 8.1.6 Overall Survival / End of Study

- This is a new section to explain patient will now be followed up past 12 month follow up for overall survival only.

Section 8.2 Procedures for assessing Efficacy

- Description of assessments updated.
- Grading tables removed and transferred to Appendix D.
-

Section 8.3 'Procedures for Assessing Safety'

- Only post-operative complications will now be reported.
- Guidance on how to grade complications added.
- Clavien Dindo Classification of Surgical Complications table transferred to Appendix D.

Section 8.4.2: Special Assays or Procedures

- Wording updated to explain that the crucial steps for performing the anastomosis were formalised following the pilot phase.

Section 8.5: Substudies

- Wording amended to clarify that samples will be sent to Liverpool when requested.

Section 9.2: Pilot

New wording added to explain the pilot phase is now complete

Section 9.3: Standardisation of surgical procedures

- Wording updated explain that the Operative Manual was formalised after the feasibility phase.

Section 9.4: Operative Photographs

- Wording amened to provide a clearer explanation of operative photographs.

Section 10: Statistical Considerations

- Overall survival added as a secondary endpoint.

Section 11: Safety

- Only surgical complications will be reported.
- Surgical complications that meet the definition of serious will continue to be reported as an SAE.
- Addition of a definition of surgical complications.
- Notes on serious complications inclusions and exclusions updated.
- Guidance on the grading of complications updated; Clavien Dindo Classification of surgical complications to be used for all serious complications. This should also be used for grading all non-serious complications except for post-operative pancreatic fistula, delayed gastric emptying and post-operative bleeding which each have an associated grading system.
- Relationship to the trial procedure updated and causality to be assigned to a) anaesthetic, b) generality of surgery, c) whipples procedure and d) anastomosis.
- Expectedness to be assessed against a) whipples procedure and b) anastomosis.
- Definition of expected and unexpected complications updated.
- Guidance on the reporting of serious complications updated.

20 REFERENCES

1. Kausch W, *Das carcinom der papilla duodeni und seine radikale Entfernung.* Beitr Z Klin Chir, 1912. **78**: p. 439-486.
2. Jones L, et al., *Standard Kausch-Whipple Pancreatoduodenectomy.* Dig Surg, 1999. **16**: p. 297-304.
3. Whipple AO, Parsons WB, and Mullens CR, *Treatment of carcinoma of the papilla of vater.* Ann Surg, 1935. **102**: p. 763-769.
4. Trimble IR, Parsons JW, and Sherman CP, *A one stage operation for cure of carcinoma of the ampulla of vater and of the head of the pancreas.* Surg Gynecol Obstet, 1941. **73**: p. 711-722.
5. Whipple AO, *Observations on radical surgery for lesions of pancreas.* Surg Gynecol Obstet, 1946. **82**: p. 623-631.
6. Watson K, *Carcinoma of the ampulla of Vater: Sucessful radical resection.* Br J Surg, 1944. **31**: p. 368-373.
7. Traverso LW and Longmire WP Jr, *Preservation of the pylorus in pancreatoduodenectomy.* Surg Gynecol Obstet, 1978. **146**: p. 959-962.
8. Pitt HA, *Curative treatment for pancreatic neoplasms: standard resection.* Surg Clin North Am, 1995. **75**: p. 891-904.
9. Seiler CA, et al., *Randomised clinical trial of pylorus-preserving duodenopancreatectomy versus classical Whipple resection-long term results.* Br J Surg, 2005. **92**: p. 547-556.
10. Diener MK, et al., *Pancreaticoduodenectomy (classic Whipple) versus pylorus-preserving pancreaticoduodenectomy (pp Whipple) for surgical treatment of periampullary and pancreatic carcinoma.* Cochrane Database Syst Rev, 2008. **16**: p. CD006053.
11. Diener MK, et al., *A systematic review and meta-analysis of pylorus-preserving versus classical pancreaticoduodenectomy for surgical treatment of periampullary and pancreatic carcinoma.* Ann Surg, 2007. **245**: p. 187-200.
12. Alexakis N, et al., *Current standards of surgery for pancreatic cancer.* Br J Surg, 2004. **91**: p. 1410-1427.
13. Halloran CM, et al., *Complications of pancreatic cancer resection.* Dig Surg., 2002. **19**: p. 138-146.
14. Bassi C, et al., *Postoperative pancreatic fistula: an international study group (ISGPF) definition.* Surgery, 2005. **138**: p. 8-13.
15. Edge SB, et al., *Pancreatic cancer resectional outcome in American University Centers in 1988-1990.* Cancer, 1993. **71**(11): p. 3502-3508.
16. Gordon TA, et al., *The effect of regionalization on cost and outcome for one general high-risk surgical procedure.* Ann Surg, 1995. **221**: p. 43-9.
17. Rosenberg I, MacNeil P, and Turcotte L, *Economic evaluation of the use of octreotide for prevention of complications following pancreatic resection.* J Gastrointest Surg, 1999. **3**: p. 225-32.
18. Schaefer CJ, *Cost and outcome of the Whipple procedure.* Ann Surg, 1995. **222**: p. 211-2.
19. Rosemurgy A, et al., *Frequency with which surgeons undertake pancreaticoduodenectomy continues to determine length of stay, hospital charges and in-hospital mortality.* J Gastrointest Surg, 2008. **12**: p. 442-449.
20. Tran K, et al., *Occulsion of the pancreatic duct versus pancreaticojejunostomy.* Ann Surg, 2002. **236**: p. 422-428.

21. Kleespies A, et al., *The challenge of pancreatic anastomosis.* Langenbecks Arch Surg, 2008. **393**: p. 459-471.
22. McKay A, et al., *Meta-analysis of pancreaticojejunostomy versus pancreaticogastrostomy reconstruction after pancreaticoduodenectomy.* Br J Surg, 2006. **93**: p. 929-936.
23. Wente MN, et al., *Pancreaticojejunostomy versus pancreaticogastrostomy: systematic review and meta-analysis.* Am J Surg, 2007. **193**: p. 171-183.
24. Bassi C, et al., *Duct-to-mucosa versus end-to-side pancreaticojejunostomy reconstruction after pancreaticoduodenectomy: results of a prospective randomised trial.* Surgery, 2003. **134**: p. 766-771.
25. Berger AC, et al., *Does type of pancreaticojejunostomy after pancreaticoduodenectomy decrease rate of pancreatic fistula? A randomised, prospective, dual-institution trial.* J Am Coll Surg, 2009. **208**: p. 738-747.
26. Langrehr JM, et al., *Prospective randomised comparison between a new mattress technique and Cattell (duct-to-mucosa) pancreaticojejunostomy for pancreatic resection.* World J Surg, 2005. **29**: p. 1111-1119.
27. Peng SY, et al., *Conventional versus binding pancreaticojejunostomy after pancreaticoduodenectomy: a prospective randomised trial.* Ann Surg, 2007(245): p. 692-698.
28. Batignani G, et al., *Comparison of Wirsung-jejunal duct-to-mucosa and dunking technique for pancreatojejunostomy after pancreatoduodenectomy.* Hepatobiliary Pancreat Dis Int, 2005. **4**: p. 450-455.
29. You D, et al., *Comparison of different pancreatic anastomosis techniques using the definitions of the International Study Group of Pancreatic Surgery: a single surgeon's experience.* Pancreas, 2009. **38**: p. 896-902.
30. Satoi S, et al., *A new guideline to reduce postoperative morbidity after pancreaticoduodenectomy.* Pancreas, 2008. **37**: p. 128-133.
31. Lee SE, et al., *Pancreatic fistula after pancreaticoduodenectomy: a comparison between the two pancreaticojejunostomy methods for approximating the pancreatic parenchyma to the jejunal seromuscular layer: interrupted vs continuous stitches.* World J Gastroenterol, 2007. **13**: p. 5351-5356.
32. Fragulidis GP, et al., *Pancreatic leakage after pancreaticoduodenectomy: the impact of the isolated jejunal loop length and anastomotic technique of the pancreatic stump.* Pancreas, 2009. **38**: p. 177-182.
33. Grobmyer SR, et al., *Novel pancreaticojejunostomy with a low rate of anastomotic failure-related complications.* J Am Coll Surg, 2010. **210**: p. 54-9.
34. Mishra PK, et al., *Blumgart's technique of pancreaticojejunostomy: an appraisal.* Dig Surg, 2011. **28**: p. 281-287.
35. Kleespies A, et al., *Blumgart anastomosis for pancreaticojejunostomy minimizes severe complications after pancreatic head resection.* Br J Surg, 2009. **96**: p. 741-750.
36. Wente MN et al.: Delayed gastric emptying (DGE) after pancreatic surgery: a suggested definition by the International Study Group of Pancreatic Surgery (ISGPS). Surgery 2007 Nov, 142 (5): 761-8
37. Horan TC et al. CDC definitions of nosocomial surgical site infections, 1992: a modification of CDC definitions of surgical wound infections. Infect Control Hosp Epidemiol 1992; 13(10): 606-8.
38. Shaw VE et al. Serum cytokine biomarker panels for discriminating pancreatic cancer from benign pancreatic disease. Mol Cancer. 2014;13:114.

39. Altman DG et al. The revised CONSORT statement for reporting randomised trials: explanation and elaboration. Ann Intern Med. 2001 Apr 17;134(8):663–94.
40. Wente MN, Veit JA, Bassi C, Dervenis C, Fingerhut A, Gouma DJ, Izbicki JR, Neoptolemos JP, Padbury RT, Sarr MG, Yeo CJ, Buchler MW. Postpancreatectomy haemorrhage (PPH): an International Study Group of Pancreatic Surgery (ISGPS) definition. Surgery. 2007 Jul; 142(1):20-5.
41. Dindo D, Demartines N, Clavien PA. Classification of surgical complications: a new proposal with evaluation in a cohort of 6336 patients and results of a survey. Ann Surg. 2004 Aug;240(2):205-13.

APPENDICES

APPENDIX A: Tables

Table 1 RCT Pancreatic Anastomosis

Study	Patients	Technique	Assignment	Octreotide	Stent	Outcome
Bassi et al 2003 [24]	144	Invagination (Single layer int.)	72	All	Some (external)	**No difference**
		Duct to Mucosa (Single layer int.)	72			
Langrehr Et al 2005 [26]	113	Invagination (mattress)	57	Some (NS)	No	**No difference**
		Duct to Mucosa (Cont (front/back)	56			
Peng et al 2007 [27]	217	Invagination (2 layer int.)	111	No	No	Leak = 7% Comps = 37%
		Binding	106			Leak = 0%* Comps + 25%** (*p=0.014, **p=0.048)
Berger et al 2009 [25]	197	Invaginating (2 layer int. including duct)	100	No	?All	Leak =24% (p=0.04)
		Duct to Mucosa (CWA.)	97			Leak =24% Potential bias to a softer gland. Centre bias

Table 2 Non-RCT Pancreatic Anastomosis

Study	Patients	Technique	Assignment	Octreotide	Stent	Outcome
Batignani et al 2005 [28]	23	Invagination	6	Not recorded	All	A non-significant increase in leaks from the non-dilated duct to mucosa anastomosis
		Duct to mucosa (dilated)	9			
		Duct to mucosa (not dilated)	8			
Lee et al 2007 [31]	303	Duct to mucosa (Int.)	133	Some (NS)	All	Discontinuous time periods – Int. 1997-2000, cont. 2001-2004, Significant bias from misclassification of fistula. **No difference**
		Duct to mucosa (Cont)	170			
Satoi et al 2008 [30]	128	Invagination	77	Some	Some	Discontinuous time periods: Invagination 2000-2004. Kakita from 2004. Kakita reduced fistula rate from 27% to 14% (ns), although less B and C (p=0.04). Reduced complications (p=0.01)
		Mod. Kakita	51			
Fragulidis et al 2009 [32]	132	Invagination – short limb	32	None	Not Stated	Concludes long limb (p=0.048) and duct to mucosa (p=0.041) is better. However no diff between invagination or duct/mucosa on short limb, but is on a long limb (p=0.04)? Effect all form long limb.
		Duct/mucosa – short limb	31			
		Invagination – long limb	30			
		Duct/mucosa – long limb	39			
You et al, 2009 [29]	119	Invagination	39	All	Most	**No difference**
		Duct/mucosa	40			
		Modified	40			
Kleespies et al. 2009 [35]	182	Duct/mucosa	90	Some (NS)	Not stated	**BA lower fistula rate** 13% vs. 4% (p=0.03). **Reduced Complications,** 31% vs. 15% (p=0.005)
		Duct/Mucosa (BA)	92			

Appendix B: EORTC Quality of Life Questionnaire (QLQ-C30)

ENGLISH

EORTC QLQ-C30 (version 3)

We are interested in some things about you and your health. Please answer all of the questions yourself by circling the number that best applies to you. There are no "right" or "wrong" answers. The information that you provide will remain strictly confidential.

Please fill in your initials: |_|_|_|_|
Your birthdate (Day, Month, Year): |_|_|_|_|_|_|_|_|
Today's date (Day, Month, Year): 31 |_|_|_|_|_|_|_|_|

	Not at All	A Little	Quite a Bit	Very Much
1. Do you have any trouble doing strenuous activities, like carrying a heavy shopping bag or a suitcase?	1	2	3	4
2. Do you have any trouble taking a long walk?	1	2	3	4
3. Do you have any trouble taking a short walk outside of the house?	1	2	3	4
4. Do you need to stay in bed or a chair during the day?	1	2	3	4
5. Do you need help with eating, dressing, washing yourself or using the toilet?	1	2	3	4

During the past week:	Not at All	A Little	Quite a Bit	Very Much
6. Were you limited in doing either your work or other daily activities?	1	2	3	4
7. Were you limited in pursuing your hobbies or other leisure time activities?	1	2	3	4
8. Were you short of breath?	1	2	3	4
9. Have you had pain?	1	2	3	4
10. Did you need to rest?	1	2	3	4
11. Have you had trouble sleeping?	1	2	3	4
12. Have you felt weak?	1	2	3	4
13. Have you lacked appetite?	1	2	3	4
14. Have you felt nauseated?	1	2	3	4
15. Have you vomited?	1	2	3	4
16. Have you been constipated?	1	2	3	4

Please go on to the next page

ENGLISH

**During the past week:**	**Not at All**	**A Little**	**Quite a Bit**	**Very Much**
17. Have you had diarrhea?	1	2	3	4
18. Were you tired?	1	2	3	4
19. Did pain interfere with your daily activities?	1	2	3	4
20. Have you had difficulty in concentrating on things, like reading a newspaper or watching television?	1	2	3	4
21. Did you feel tense?	1	2	3	4
22. Did you worry?	1	2	3	4
23. Did you feel irritable?	1	2	3	4
24. Did you feel depressed?	1	2	3	4
25. Have you had difficulty remembering things?	1	2	3	4
26. Has your physical condition or medical treatment interfered with your <u>family</u> life?	1	2	3	4
27. Has your physical condition or medical treatment interfered with your <u>social</u> activities?	1	2	3	4
28. Has your physical condition or medical treatment caused you financial difficulties?	1	2	3	4

For the following questions please circle the number between 1 and 7 that best applies to you

29. How would you rate your overall health during the past week?

1	2	3	4	5	6	7
Very poor						Excellent

30. How would you rate your overall quality of life during the past week?

1	2	3	4	5	6	7
Very poor						Excellent

© Copyright 1995 EORTC Quality of Life Group. All rights reserved. Version 3.0

Appendix C: EQ-5D-3L

EQ-5D-3L

Health Questionnaire

English version for the UK
(validated for Ireland)

UK (English) © 1990 EuroQol Group EQ-5D™ is a trade mark of the EuroQol Group

By placing a tick in one box in each group below, please indicate which statements best describe your own health state today.

Mobility

I have no problems in walking about ☐

I have some problems in walking about ☐

I am confined to bed ☐

Self-Care

I have no problems with self-care ☐

I have some problems washing or dressing myself ☐

I am unable to wash or dress myself ☐

Usual Activities *(e.g. work, study, housework, family or leisure activities)*

I have no problems with performing my usual activities ☐

I have some problems with performing my usual activities ☐

I am unable to perform my usual activities ☐

Pain/Discomfort

I have no pain or discomfort ☐

I have moderate pain or discomfort ☐

I have extreme pain or discomfort ☐

Anxiety/Depression

I am not anxious or depressed ☐

I am moderately anxious or depressed ☐

I am extremely anxious or depressed ☐

2

UK (English) © 1990 EuroQol Group EQ-5D™ is a trade mark of the EuroQol Group

To help people say how good or bad a health state is, we have drawn a scale (rather like a thermometer) on which the best state you can imagine is marked 100 and the worst state you can imagine is marked 0.

We would like you to indicate on this scale how good or bad your own health is today, in your opinion. Please do this by drawing a line from the box below to whichever point on the scale indicates how good or bad your health state is today.

Your own health state today

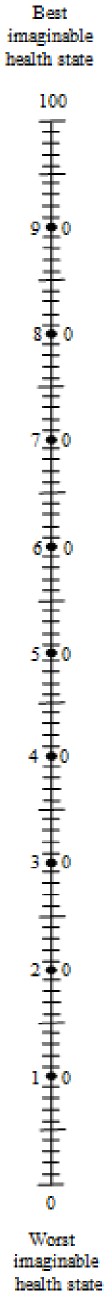

UK (English) © 1990 EuroQol Group EQ-5D™ is a trade mark of the EuroQol Group

Appendix D: Grading Tables

Table 1: Clavien Dindo Classification of Surgical Complications

TABLE 1. Classification of Surgical Complications

Grade	Definition
Grade I	Any deviation from the normal postoperative course without the need for pharmacological treatment or surgical, endoscopic, and radiological interventions<br>Allowed therapeutic regimens are: drugs as antiemetics, antipyretics, analgetics, diuretics, electrolytes, and physiotherapy. This grade also includes wound infections opened at the bedside
Grade II	Requiring pharmacological treatment with drugs other than such allowed for grade I complications<br>Blood transfusions and total parenteral nutrition are also included
Grade III	Requiring surgical, endoscopic or radiological intervention
Grade IIIa	Intervention not under general anesthesia
Grade IIIb	Intervention under general anesthesia
Grade IV	Life-threatening complication (including CNS complications)* requiring IC/ICU management
Grade IVa	Single organ dysfunction (including dialysis)
Grade IVb	Multiorgan dysfunction
Grade V	Death of a patient
Suffix "d"	If the patient suffers from a complication at the time of discharge (see examples in Table 2), the suffix "d" (for "disability") is added to the respective grade of complication. This label indicates the need for a follow-up to fully evaluate the complication.

*Brain hemorrhage, ischemic stroke, subarrachnoidal bleeding, but excluding transient ischemic attacks.
CNS, central nervous system; IC, intermediate care; ICU, intensive care unit.

Table 2: Post-Operative Pancreatic Fistula Grading Table

Grade A:	Grade B:	Grade C:
Without clinical impact	Clinically relevant	Clinical stability may be borderline
Oral nutrition	Partial/total parental/enteral nutrition	Treatment in an intensive care unit in many cases
No antibiotics	Peripancreatic collection possible	Total parental/enteral nutrition
No somatostatin analogues	Abdominal pain, fever, and/or leucocytosis possible	Intravenous antibiotics and somatostatin analogues necessary
No peripancreatic fluid collection	Antibiotics and somatostatin analogues may be necessary	Worrisome peripancreatic fluid collection that requires percutaneous drainage
No delay in hospital discharge	Delay in hospital discharge or readmission may be required.	Extended hospital stay
		Often associated complications and postoperative mortality possible

Table 3: Delayed Gastric Emptying Grading Table

DGE Grade	NGT required	Unable to tolerate solid food by POD	Vomitting / Gastric distension	Use of Prokinetics
A	4-7 days or reinsertion>POD	7	±	±
B	8-14 days or reinsertion>POD	14	+	+
C	>14 days or reinsertion	21	+	+

Table 4: Classification of post pancreatectomy haemorrhage

Grade	Time of onset, location, severity and clinical impact of bleeding		Clinical condition	Diagnostic consequence	Therapeutic consequence
A	Early, intra- or extraluminal, mild		Well	Observation, blood count, ultrasonography and, if necessary, computed tomography	No
B	Early, intra- or extraluminal, severe	Late, intra- or extraluminal, mild	Often well/ intermediate, very rarely life-threatening	Observation, blood count, ultrasonography, computed tomography, angiography, endoscopy‡	Transfusion of fluid/blood, intermediate care unit (or ICU), therapeutic endoscopy,‡embolization, relaparotomy for early PPH
C		Late, intra- or extraluminal, severe	Severely impaired, life-threatening	Angiography, computed tomography,	
